# Influence of gene modification in biological behaviors and responses of mouse lung telocytes to inflammation

**DOI:** 10.1186/s12967-019-1870-y

**Published:** 2019-05-15

**Authors:** Dongli Song, Menglin Xu, Ruixue Qi, Ruihua Ma, Yile Zhou, Duojiao Wu, Hao Fang, Xiangdong Wang

**Affiliations:** 1 Zhongshan Hospital Institute of Clinical Science, Shanghai Institute of Clinical Bioinformatics, Shanghai Engineering Research for AI Technology for Cardiopulmonary Diseases, Shanghai, China; 2 Center for Tumor Diagnosis and Therapy, Zhongshan Hospital Jinshan Hospital, Shanghai Medical College, Fudan University, Shanghai, China; 3 Department of Anesthesiology, Zhongshan Hospital, Shanghai, China; 4 Department of Anesthesiology, Minhang Branch, Zhongshan Hospital, Fudan University, Shanghai, China

**Keywords:** Telocytes, Gene modification, Lung, SV40, Transformation

## Abstract

**Background:**

Telocytes play key roles in maintenance of organ/tissue function and prevention of organ injury. However, there are great challenges to investigate telocytes functions using primary telocytes, due to the difficulties of isolation, identification, and stability. The present study aims at constructing continuous cell strain of mouse lung telocyte cell line with stable characters by gene modification and investigating biological behaviors and responses of gene-modified telocytes to inflammation.

**Methods:**

Mouse primary lung telocytes were isolated and identified using immune-labeling markers and immunoelectron microscopy. Primary telocytes were transformed with Simian vacuolating virus 40 small and large T antigen (SV40). Biological characters, behaviors morphology, and proliferation of those gene-modified telocytes were defined and monitored dynamically for 50 generations, as compared with primary lung telocytes. Cell cycle of mouse primary lung telocytes or gene-modified telocytes was detected by flow cytometry.

**Results:**

Gene modified telocytes of generations 5, 10, 30 and 50 were observed with telopodes and also showed CD34 and ckit positive. Multiple cellular morphology were also observed on telocyte cell-line under monitor of celliq and enhanced cell proliferation were showed. SV40 transduction was also reduced apoptosis and increased the ratio of S and G2 phases in telocyte cell-line.

**Conclusion:**

We successfully constructed mouse lung telocyte cell-line which maintained the biological properties and behaviors as primary telocytes and could responses to inflammation induced by LPS. Thus, gene-modified lung telocytes, Telocyte Line, would provide a cell tool for researchers exploring the roles and applications of telocytes involved in physiological and pathological states in future.

**Electronic supplementary material:**

The online version of this article (10.1186/s12967-019-1870-y) contains supplementary material, which is available to authorized users.

## Background

Telocytes (TCs) have been found widely spreaded in a large number of organs and tissues of mammals, including atrial and ventricular myocardium, bladder, lung skeletal muscle, gastrointestinal tract, eyes, and others. TCs communicate with neighboring cells by homo- and heterocellular contacts and transfer genetic information and signaling molecules to influence other cells [1, 2]. Shoshkes-Carme et al. [3] recently demonstrated that TCs can be the major source of Wnt signaling, and play dependent roles in proliferation of intestinal stem cells and epithelial renewal. This particular study provides solid evidence that forkhead box L1-positive TCs contribute to the formation of the subepithelial plexus of the intestine, support the entire epithelium, and provide niche signals to intestinal stem cells, by producing Wnt ligands and maintaining Wnt signal pathway. Although TCs are often allocated within the stem cell niche, pulmonary TCs have different genetic profiles and biological functions from stem cells, fibroblasts, alveolar type II cells, airway basal cells, proximal airway cells, and T cells from bronchial lymph nodes or from lungs, respectively [4–7]. TCs functionally and constructively support and connect tissue cells, function as signal massage carriers, and benefit gene and cell therapies [8–12].

Gene modification is considered as a potential therapy to genetically prevent and treat diseases, even though a large number of factors still need to furthermore clarified, e.g. safety, efficacy, and complexity. With rapid development of gene editing technology, the precision and efficacy of gene modification are improved significantly [13, 14]. Safety profiles of gene modification as part of limitations require more preclinical and clinical evidence. The present study investigates potential effects of gene modification on morphological phenomes, biological behaviors and functions, as well as responses to inflammation in lung TCs. An anti-aging gene from Simian vacuolating virus 40 was transferred as the target gene into primary TCs isolated from mouse lungs to improve the longevity of cells, since primary TCs hardly survive in the in vitro system for a few weeks or months [5–7].

## Materials and methods

### TCs isolation and primary cell culture

Mice were provided by Animal Facility in Biomedical Research Center of Zhongshan Hospital, Fudan University. This study was approved by the Fudan University Ethical Committee for animal experiments.

Mouse lung TCs were isolated and prepared as escribed previously [4]. In brief, female BABL/c mice aged 6–8 weeks were used and anaesthetized. The trachea and lung tissues were isolated and collected into sterile tubes, containing Dulbecco’s Modified Eagle’s Medium plus F12 (DMEM/F12) integration (Gibco, NY, USA), supplemented with 100 UI/ml penicillin, 0.1 mg/ml streptomycin (Sigma-Aldrich Shanghai Trading Co Ltd., Shanghai, China). Samples were placed on ice and transported to the cell culture laboratory within 30 min. The tissues were cut into about 1 × mm^3^ in sterile DMEM/F12 and incubated in 10 mg/ml collagenase type II (Sigma-Aldrich, St. Louis, MO, USA) and 2000 U/ml deoxyribonuclease I (Sigma-Aldrich) on an orbital shaker for 4 h, at 37 °C. Cell suspension was separated by 40-m-diameter cell strainer (BD Falcon, NJ, USA), and dispersed cells were collected by centrifugation. Cells were resuspended in DMEM/F12, supplemented with 10% fetal calf serum, 100 UI/ml penicillin, 0.1 mg/ml streptomycin (Sigma-Aldrich), and cultured in a incubator, with 5% CO_2_ in air, at 37 °C, for 30 min, to wipe off most fibroblasts. Cell suspension was transferred to other flakes and cultured for another 12 h before changing culture medium. Culture medium was changed every 48 h. Cells were examined by phase contrast microscope, under an inverted Olympus phase contrast microscope (1 × 51; Tokyo, Japan).

### Lentivirus construction and infection

Lentivirus particles containing the small and large T antigen of anti-aging gene from Simian vacuolating virus 40 (SV40) gene were constructed (HanyinCo., Shanghai, China) according to the former articles [15]. The oligo (listed 5′–3′) SV40-F: CCG GAATTCATGGATAAAGTTTTAAACAGAGAGGAATC and SV40-R: GCTCTAGATTACAAGTCCTCTTCAGAAATGAG. TCs were infected on 6-well plates for 12 h at 37 °C, replaced fresh medium, and harvested for qPCR analysis to determine the SV40 mRNA expression level after being cultured for 96 h at 37 °C.

### RNA extraction and PCR

Primary TCs and TCs transformed with SV40 (TCs^SV40^) were seeded in 24-well plates with a density of 10^4^ cells/well and cultured for 24 h at 37 °C. Cells were washed thrice with PBS, and total RNA was isolated and transcribed into single-stranded cDNA using the 1st Strand cDNA Synthesis Kit for RT-PCR (AMV, Roche) following the recommendations of the manufacturer.

cDNA was synthesized from 1 µg of total RNA using PrimeScript^®^ RT reagent Kit (Takara Bio Inc., Shiga, Japan). PCR was performed with 1 µl of cDNA using GoTaq polymerase (Promega) for 25 cycles with specific primers for SV40. PCR reaction products were resolved and stained with Gelred (Biotium Inc., Newark, USA).

### Detection of cell bio-behaviors

The bio-behaviors of TCs and TCs^SV40^ were recorded and analyzed using a Cell-IQ cell culturing platform (Chip-Man Technologies, Tampere, Finland), equipped with a phase-contrast microscope (Nikon CFI Achromat phase contrast objective with 10 magnification) and a camera20. The bio-behaviors, including cell proliferation, division, death, cell morphology, and cell movement, can be monitored and recorded as time-lapse data by this Cell-IQ system uses machine vision technology. Images were captured at about 30 min intervals for 48 h. Analysis was carried out with a freely distributed Image software (McMaster Biophotonics Facility, Hamilton, ON), using the Manual Tracking plugin created by Fabrice Cordelie´res (Institute Curie, Orsay, France).

### RNA microarrays and long non-coding RNA (lncRNA) classification pipeline

RNA microarrays and lncRNA classification pipeline were tested in primary TCs or TCs^SV40^. Briefly, total RNA was collected using NucleoSpin^®^ RNA Plus according the manufacturer’s protocol (Macherey–Nagel, Inc., Düren, Germany). Microarray and quality controls of gene expression profiling were performed after RNA and cDNA amplifications, using the GeneChip^®^ Human Transcriptome Array 2.0 gene chip (Affymetrix, Inc., UK) with 67,528 genes. Gene expression data from each group were analyzed using Expression Console and Transcriptome Analysis Console 3.0.0.466 (Affymetrix). The differentially expressed mRNA and lncRNAs were used for a hierarchical clustering analysis (HCA) in Cluster and TreeView (https://sourceforge.net/projects/jtreeview/files/).

### Immunofluorescent staining

Double immunofluorescent staining for CD34 and vimentin was performed as previously reported [21]. In brief, primary TCs or TCs^SV40^ in 1, 5, 10, 30, or 50 generations were load and cultured on glass bottom cell culture dishes with 20 mm diameter glass (NEST, Nanjing, China) and were fixed in 4% paraformaldehyde containing 0.05% Triton-X-100 for 20 min. The cells were washed thrice with PBS and blocked in 5% bovine serum albumin (BSA) for 1 h and incubated overnight at 4 °C with mouse anti-CD34 antibody and goat anti-vimentin antibody or rabbit anti-ckit antibody (1:200 dilution; Abcam, Cambridge, UK) diluted in 1% BSA in PBS. Cells were washed in PBS thrice and incubated with PE conjugated anti-goat secondary antibodies and FITC conjugated anti-rabbit secondary antibodies and/or FITC conjugated anti-mouse secondary antibodies (1:200 dilution; Jackson ImmunoResearch, USA). The nuclear were marked by DAPI staining, according to the manufacture’s instruction (KeyGEN BioTECH, Nanjing, China).

### Transmission electron microscopy

The ultrastructure of cells were observed under transmission electron microscopy (TEM) as previously reported (14). In brief, primary TCs or TCs^SV40^ in 1, 5, 10, 30, and 50 generations were cultured, collected, and fixed in 4% glutaraldehyde (pH 7.3, 4 °C) for 4 h. Cells were then washed with 0.1 M cacodylate buffer and post-fixed with 1% osmium tetroxide in 0.1 M cacodylate buffer (pH 7.3, 4 °C). After fixing, cells were dehydrated in a graded series of ethanol, impregnated in propylene oxide (immersed overnight in a mixture of propylene oxide and Epon 812 resin), and embedded in Epon 812. Ultrathin sections at 70 nm were cut on a Leica LKB-II (Nußloch, Germany), collected on Formvar-coated copper grids, stained with uranyl acetate and lead citrate, and observed at an acceleration voltage of 80 kV electron microscope (JEOL JEM-1230, Tokyo, Japan).

### Immunoelectron microscopy

Ultrathin sections were prepared and collected on nickel grids. Immunolabeling staining for CD34/Vimentin and ckit/platelet-derived growth factor receptor α (PDGFR-α) was used as previously reported [22]. In brief, sections were incubated in 50 mM Glycine for 30 min and washed in Ultra-pure Water thrice for 5 min. Sections were etched in 1% sodium periodate for 10 min following washing in Ultra-pure water. Sections were incubated in the blocking buffer for 20 min and labeled with rabbit anti-ckit antibody, mouse anti-CD34 antibody, goat anti-vimentin antibody and/or rat anti PDGFR-α antibody (1:200 dilution; Abcam) at 4 °C for 24 h. The nickel grids were washed in PBS for 5 min 12 times, blocked within 1% BSA for 20 min, and incubated with 10 nm gold conjugated anti-goat secondary antibodies, 18 nm gold conjugated anti-mouse secondary antibodies, 25 nm gold conjugated anti-rat secondary antibodies, and/or 40 nm gold conjugated anti-rabbit secondary antibodies (1:200 dilution; Abcam) for 2 h. Nickel grids were dried on filter paper and observed with transmission electronic microscopy (TEM). The staining controls included cells stained only with the second antibodies with gold labelling or the first antibodies.

### Cell cycle assay

Propidium iodide (PI) staining was used for cell cycle analysis of primary TCs and TCs^SV40^ as described in manufacturer. In brief, cells were collected and fixed in 75% ethanol at 4 °C for overnight. After centrifuging and washing, staining buffer (BD Pharmingen, NJ, USA) with 0.5 ml PI/RNase was added to each tube for 15 min at room temperature. Samples were examined with a fluorescence-activated cell sorting flow cytometer (FACS Aria II, Becton, Dickinson and Company, NJ, USA) and DNA histograms were analyzed with Flowjo 7.6.1 software. Each test was repeated thrice.

### Statistics

Data were expressed as mean ± SEM analyzed using SPSS Statistics 20 (IBM, Chicago, USA). Statistical differences between two groups were compared by t-test. Statistical differences among more than two groups were determined using ANOVA. p value less than 0.05 was considered significant.

## Results

**Fig. 1:**
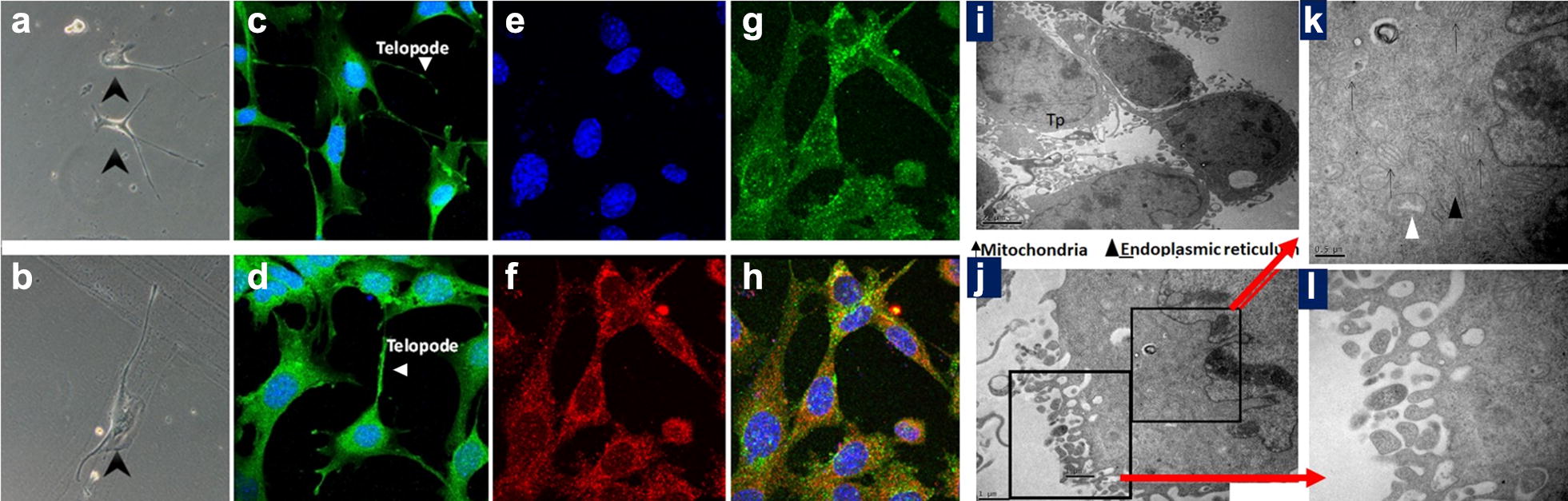
Characteristics of primary lung telocytes (TCs). **a**, **b** Telocytes with telopodes (black arrow heads), **c** positive staining of c-kit (green), and telopodes (white arrow heads), **d** positive staining CD34 (green), and telopodes (white arrow heads), **e** nuclear staining with DAPI, **f** positive staining of vimentin (red), **g** positive staining of CD34 (green), **h** merge of DAPI, vimentin, and CD34, **i** telopode ultrastructure (Tp), **j** organelle and cell membrane, **k** mitochondrial (black arrows), mitochondrial vacuoles (white arrow head) and endoplasmic reticulum (black arrow head) in the cytoplasm, and **l** microvilli under TEM

Telopodes (Tps) as one of characteristic structures of TCs were demonstrated in Fig. 1a, b. The c-kit/CD117, CD34 and vimentin in primary lung TCs were detected and shown in Fig. 1c, d, f–h. TEM tomography also showed that TCs have narrow and flat cellular prolongations surrounding other TCs in Fig. 1i, j. Mitochondria and endoplasmic reticulums in cytoplasm and nuclear of TCs were shown (Fig. 1k). Cytomembrane were also shown clearly under TEM (Fig. 1l).

**Fig. 2:**
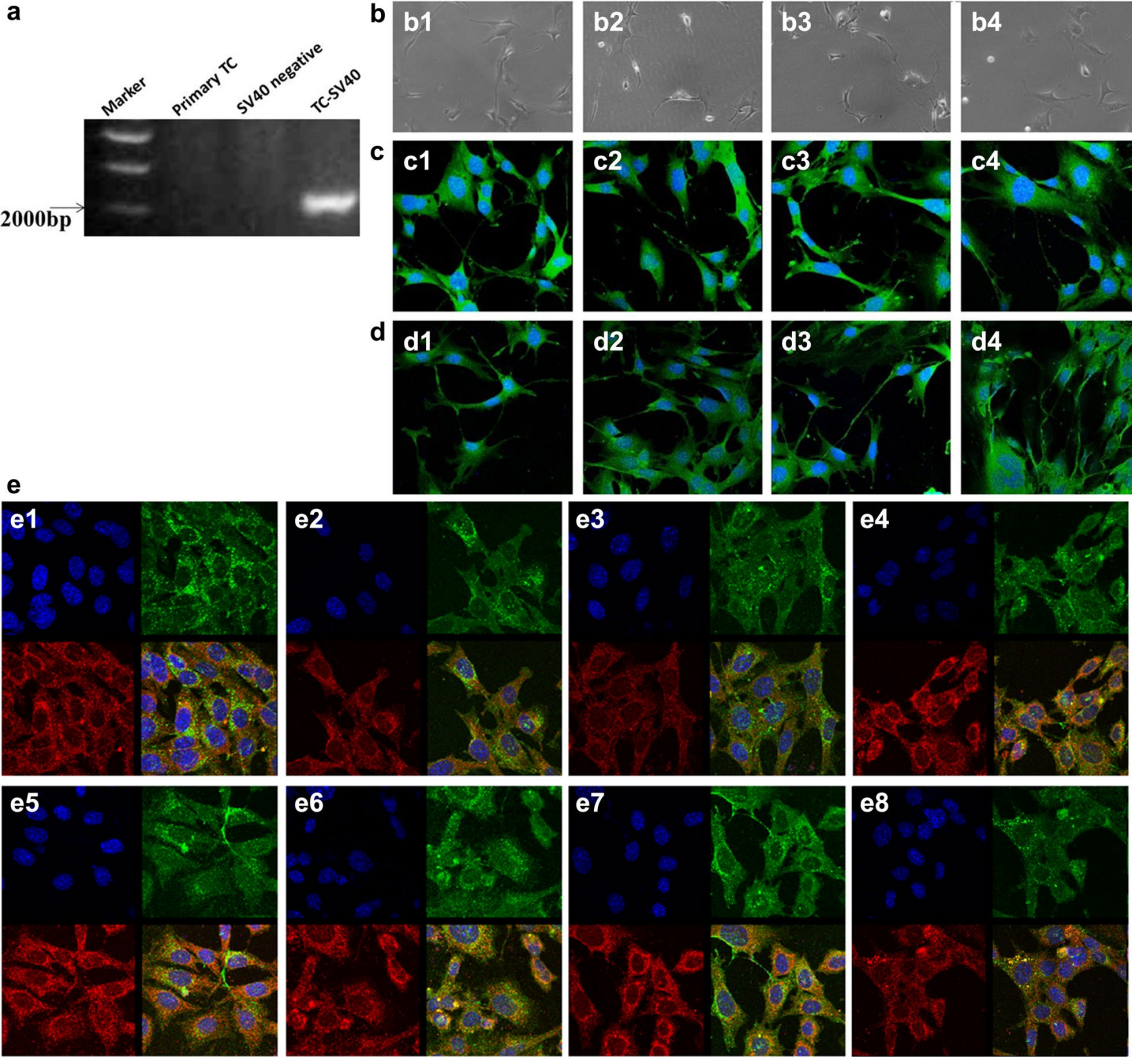
TCs characteristics of lung TCs transferred with SV40 (TCs^SV40^). **a** SV40 mRNA expression of TCs^SV40^ cells, as compared with primary lung TCs or SV40-negative cells, **b** telopodes of TCs^SV40^ at 2 (**b1**), 5 (**b2**), 10 (**b3**), or 20 generations (b4), **c** positive staining of c-kit (green) in TCs^SV40^ at 2 (**c1**), 5 (**c2**), 10 (**c3**), or 20 generations (**c4**), **d** positive staining of CD34 (green) in TCs^SV40^ at 2 (**d1**), 5 (**d2**), 10 (**d3**), or 20 generations (**d4**), and **e** positive staining of CD34 (green) and vimentin (red) in TCs^SV40^ at 2 (**e1**), 5 (**e2**), 10 (**e3**), 20 (**e4**), 30 (**e5**), 40 (**e6**), 50 (**e7**), or 60 generations (**e8**), where cell nuclei were stained with DAPI

The quality of SV40 gene insert in TCs^SV40^ were defined with SV40 mRNA expression and shown in Fig. 2a. Characteristics of telocytes in TCs^SV40^ were identified and telopodes of TCs^SV40^ were observed and recorded in Fig. 2b. The positive staining of vimentin and CD34 was detected in primary lung TCs and TCs^SV40^, as presented in Fig. 2c, d.

**Fig. 3:**
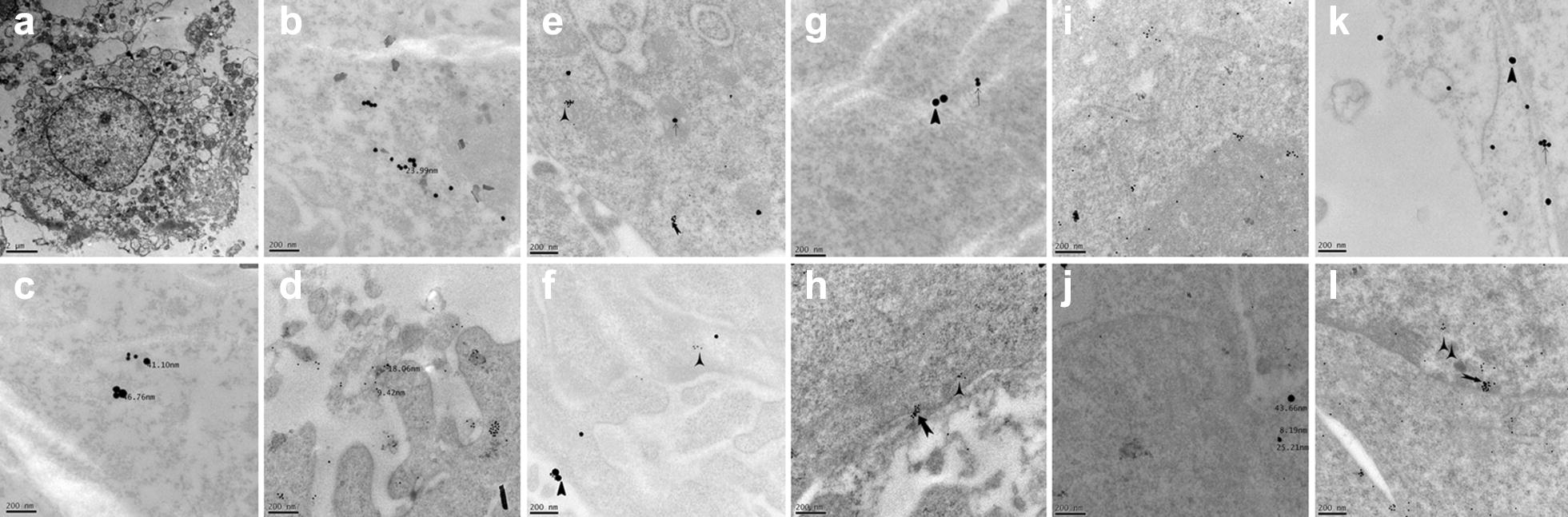
Immuno-staining transmission electronic microscopy tomography of vimentin, CD34, platelet-derived growth factor receptor α (PDGF), or ckit of lung TCs transferred with SV40 (TCs^SV40^). **a** TCsSV40 tomography, and positive staining of vimentin (  , 10 nm), CD34 (  , 18 nm), PDGF (  , 25 nm), or ckit (  , 40 nm) in TCs^SV40^ at 2 (**b**–**d**), 5 (**e**, **f**), 10 (**g**, **h**), 30 (**i**, **j**), or 50 generations (**k**, **l**)

In order to demonstrated the immortalization and stability of TCs^SV40^, telopodes were observed in TCs^SV40^ cultured for 1, 5, 10 and 20 generations SV40 mRNA positively expressed in TCs^SV40^ cells through generations, as compared with primary lung TCs or SV40-negative cells (Fig. 2a). Characteristics of TCs^SV40^ were furthermore evaluated at 2, 5, 10, or 20 generations, respectively, including telopodes (Fig. 2b1–b4), positive staining of c-kit (Fig. 2c1–c4), positive staining of CD34 (Fig. 2d1–d4), or positive staining of both CD34 and vimentin at 2 (e1), 5 (e2), 10 (e3), 20 (e4), 30 (e5), 40 (e6), 50 (e7), or 60 generations (e8), where cell nuclei were stained with DAPI. Dynamic alterations of TCs^SV40^ at 2, 5, 10, 20, 30, 50, or 60 generations were recorded automatically each 30 min for 48 h and cell morphological phenomes were presented each 12 h, respectively, in Additional file 1: Figure S1. Figure 3 demonstrated that immuno-positive staining of the vimentin labeled with particle diameter at 10 nm, CD34 at18 nm, platelet-derived growth factor receptor α (PDGFRα) at 25 nm, or ckit at 40 nm in lung TCs^SV40^ under transmission electronic microscopy. Tomography of TCs was taken immediately after transfer with SV40 tomography, to show mitochondria-rich cytoplasm and surrounded nucleus as well as telopodes with mitochondria (Fig. 3a). PDGFRα, ckit, or vimentin and CD34 were easily detected in TCs^SV40^ at 2 (b, c, d), 5 (e, f), 10 (g, h), 30 (i, j), or 50 generations (k, l), respectively. Those findings of transmission electronic microscopy tomography expression demonstrated that the characteristic structures and expressions of specific markers could be stable and consistent until generation 60.

**Fig. 4:**
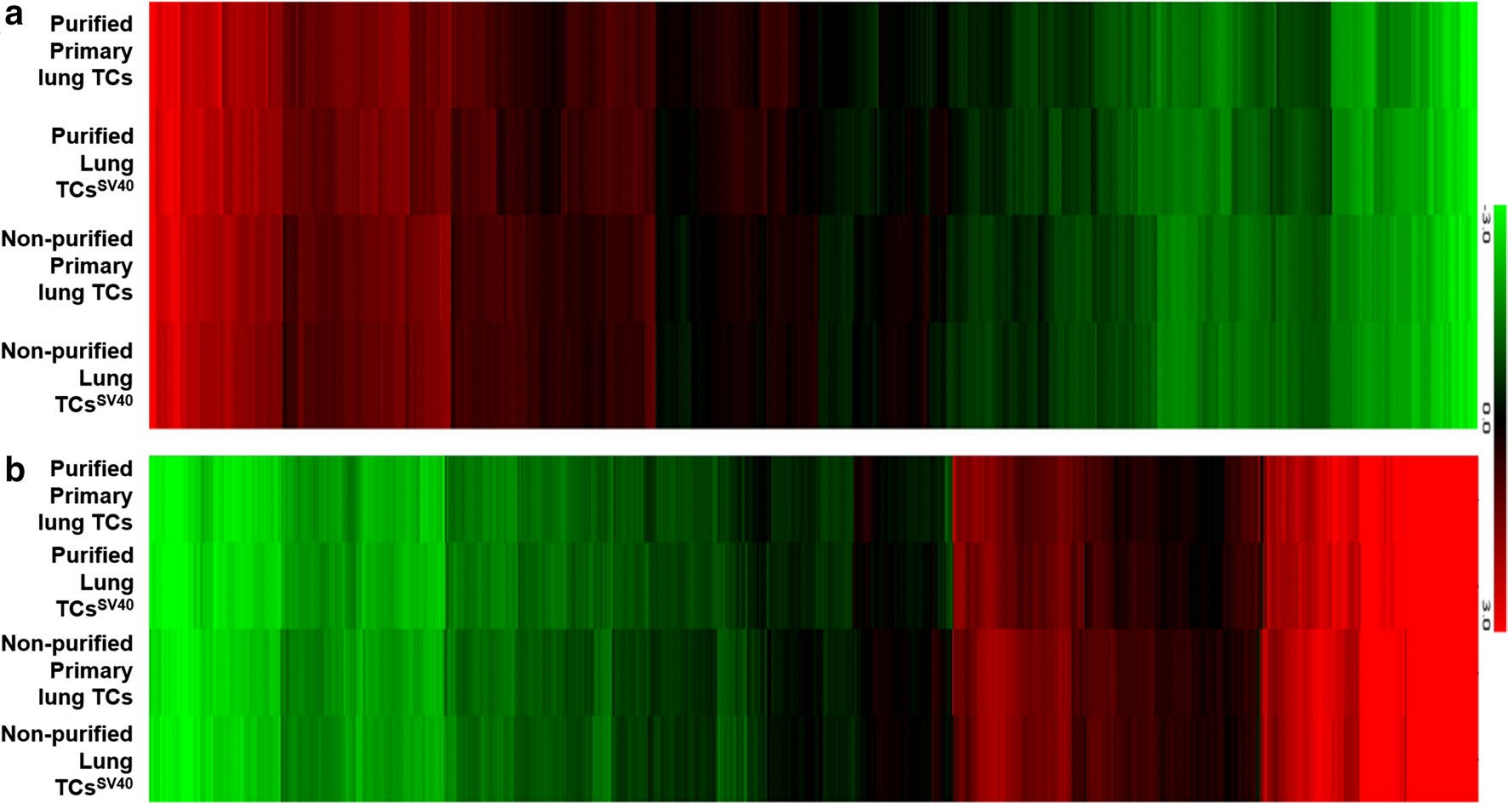
Hotmap of differential Genes of primary TCs, SV40-transformed TCs, primary lung cells, and SV40-transformed primary lung cells. **a** Hotmap of differential mRNAs. **b** Hotmap of LncRNAs of primary TCs, SV40-transformed TCs, primary lung cells, and SV40-transformed primary lung cells

The profiles of transcriptional factor and lncRNA genes between primary lung TCs and TCs^SV40^ were compared and listed in Tables 1 and 2, and the hotmap was shown in Fig. 4. As compared with purified primary lung TCs, 367 or 621 genes were up- or down-regulated in purified TCs^SV40^, 668 or 890 genes in non-purified lung TCs^SV40^, or 36 genes up-regulated in non-purified primary lung TCs. As compared with non-purified TCs^SV40^, 71 or 116 genes were up- or down-regulated in purified TCs^SV40^. Details of transcriptional factor and lncRNA gene profiles were listed in Additional file 2: Table S1. COL3A1 and SFRP2, which code alpha 1 type III collagen and secreted frizzled-related protein 2, respectively, significantly up-regulated in purified TCs^SV40^, as compared with purified primary lung TCs. The patterns of transcriptional factor and lncRNA gene profiles were shown in Fig. 4a, b, respectively). The top 5 transcriptional factor genes are COL3A1, SLIT3, FST, NNAT and PCDH17, respectively. The top 5 lncRNA genes are TC17000728.hg.1, TC06001978.hg.1, TC08000302.hg.1, TC07001784.hg.1 and TC03003114.hg.1, respectively.

**Table 1:** Summary of mRNA expressed preferentially in primary TCs, SV40-transformed TCs, primary lung cells, and SV40-transformed primary lung cells

Compared pairs	Up > 0	Up > 2	Down > 0	Down > 2
Purified TCs^SV40^ vs. non-purified primary lung TCs	158	0	124	0
Non-purified lung TCs^SV40^ vs. purified primary lung TCs	343	1	119	0
Non-purified lung TCs^SV40^ vs. non-purified primary lung TCs	274	1	151	0
Purified TCs^SV40^ vs. non-purified lung TCs^SV40^	64	0	75	0
Purified TCs^SV40^ vs. purified primary lung TCs	255	2	122	0
Purified primary lung TCs vs. non-purified primary lung TCs	0	0	23	0

Gene symbol or probe set ID	Fold change	p-value	Gene feature
(A) *Genes up/down-regulated in purified TCs*^*SV40*^ *compared with non-purified primary lung TCs*			
COL3A1	1.736972	5.20E−05	Up
SLIT3	1.428832	5.70E−05	Up
FST	1.757618	6.20E−05	Up
NNAT	1.428018	6.60E−05	Up
PCDH17	− 1.82768	7.10E−05	Down
KIAA1199	1.450952	7.60E−05	Up
BHLHE22	1.431489	8.10E−05	Up
KLHDC10	− 1.342675	8.60E−05	Down
CAPN6	− 1.502786	9.10E−05	Down
DBC1	1.42647	9.60E−05	Up
ANGPTL7	1.490275	0.000101	Up
FBN1	1.555173	0.000106	Up
IGFBP7	1.491186	0.000111	Up
COL5A2	1.521795	0.000116	Up
SPRR1B	− 1.653323	0.000121	Down
PCDH7	1.645697	0.000126	Up
MBD6	− 1.354523	0.000131	Down
PPARGC1A	− 1.46284	0.000135	Down
SFRP2	1.953208	0.00014	Up
UPRT	− 1.391618	0.000145	Down
BNC1	1.309024	0.00015	Up
TNC	1.396486	0.000155	Up
EGR1	− 1.562517	0.00016	Down
COL12A1	1.253525	0.000165	Up
ZFP36L1	1.413153	0.00017	Up
NFAT5	− 1.658072	0.000175	Down
GFPT2	1.261057	0.00018	Up
FOXP2	1.209035	0.000185	Up
TCF20	− 1.334035	0.00019	Down
STAT3	1.388462	0.000195	Up
GATA6	1.445955	0.000199	Up
CYP51A1	1.224699	0.000204	Up
IGF1	1.320664	0.000209	Up
COL1A1	1.467933	0.000214	Up
PCDH19	− 1.536647	0.000219	Down
VLDLR	− 1.263978	0.000224	Down
HOXA2	1.693458	0.000229	Up
OGT	− 1.620988	0.000234	Down
ANKH	− 1.252673	0.000239	Down
ZNF503	1.323818	0.000244	Up
PCDH10	1.55352	0.000249	Up
HIF1A	1.439987	0.000254	Up
TGFB1I1	1.228978	0.000259	Up
MED12	− 1.302994	0.000264	Down
FBLN2	1.27815	0.000268	Up
HMGCS1	1.267852	0.000273	Up
CPXM1	1.343796	0.000278	Up
ILDR2	1.398758	0.000283	Up
MID2	− 1.297559	0.000288	Down
SEMA3A	− 1.339824	0.000293	Down
PTGS2	− 1.251508	0.000298	Down
BICC1	1.416447	0.000303	Up
KMT2D	− 1.222378	0.000318	Down
NFIA	1.44573	0.000323	Up
SMO	− 1.262105	0.000328	Down
TMEM238	1.23793	0.000332	Up
DNAJC1	1.241613	0.000337	Up
H2AFY2	1.200548	0.000342	Up
ARF6	1.267706	0.000347	Up
ACLY	1.32049	0.000352	Up
SPRR2F	− 1.383183	0.000357	Down
SCD	1.264224	0.000362	Up
GPC4	− 1.211023	0.000367	Down
KLF9	1.351626	0.000372	Up
ITPR3	− 1.227564	0.000377	Down
CDH11	1.285389	0.000382	Up
NXN	1.291856	0.000392	Up
MMP2	1.261064	0.000397	Up
GPM6B	1.298864	0.000401	Up
GRIA3	1.443882	0.000406	Up
CCND2	− 1.243142	0.000411	Down
LOX	1.244763	0.000416	Up
MSI2	− 1.254073	0.000421	Down
FAM126A	− 1.296542	0.000426	Down
TC11000974.hg.1	− 1.348845	0.000431	Down
DOCK9	− 1.213445	0.000446	Down
TRBV21OR9-2	− 1.381268	0.000451	Down
THBS1	1.376867	0.000456	Up
PLK1	1.251123	0.000461	Up
DDX26B	− 1.339568	0.000465	Down
FRMD5	1.270501	0.00047	Up
ADAMTS15	1.347894	0.000475	Up
MBNL1	− 1.406503	0.00048	Down
AZIN1	− 1.31031	0.000485	Down
WT1	1.203106	0.00049	Up
COL1A2	1.333456	5.00E−04	Up
ISM1	1.372788	0.000505	Up
PRRX1	1.396349	0.00051	Up
IGIP	− 1.26922	0.000515	Down
TSHZ1	1.31218	0.00052	Up
TNPO3	− 1.26636	0.000525	Down
FAM98A	1.240276	0.00053	Up
HSPA5	1.505916	0.000534	Up
PRG4	1.279794	0.000539	Up
UGCG	1.205908	0.000554	Up
CDKL5	− 1.282448	0.000559	Down
HOXB8	1.214483	0.000564	Up
RDH10	1.230479	0.000569	Up
ARL6IP1	1.267388	0.000574	Up
RNF150	1.296804	0.000594	Up
TBPL1	− 1.345451	0.000598	Down
KLF7	1.240203	0.000603	Up
ID4	1.227502	0.000608	Up
MBTPS2	− 1.372487	0.000618	Down
AHCYL2	− 1.2362	0.000623	Down
C9orf172	1.232029	0.000628	Up
THBS2	1.234081	0.000633	Up
IER5L	1.31756	0.000638	Up
FZD8	1.478565	0.000643	Up
REXO2	1.287723	0.000658	Up
MAN1A1	1.214421	0.000663	Up
PLXDC2	1.315137	0.000677	Up
DPYSL2	1.20111	0.000682	Up
INTS5	1.320636	0.000687	Up
ABCB7	− 1.288423	0.000692	Down
C3orf58	1.21307	0.000712	Up
FAM218A	1.35927	0.000717	Up
FMR1	1.401071	0.000722	Up
PCDHGC5	1.328006	0.000731	Up
TRPS1	− 1.275263	0.000736	Down
GPC6	1.24421	0.000741	Up
TC07000141.hg.1	− 1.446989	0.000771	Down
SEC23A	1.295262	0.000776	Up
ZDHHC15	− 1.240478	0.000786	Down
SMAD7	1.261408	0.000791	Up
GPC3	1.218435	0.000796	Up
TUBB4B	1.245339	0.00081	Up
TXNIP	1.202009	0.00082	Up
FIGN	1.385827	0.000825	Up
TC02002617.hg.1	− 1.775002	0.00084	Down
DTNA	− 1.217215	0.00085	Down
MEIS1	1.369642	0.000855	Up
PLK2	− 1.202425	0.00086	Down
RBPJ	− 1.218305	0.000864	Down
NPR2	− 1.309717	0.000869	Down
NID1	1.228805	0.000874	Up
AKT3	− 1.201507	0.000879	Down
SAT1	− 1.219609	0.000884	Down
SURF4	1.201325	0.000889	Up
CELF2	1.384512	0.000914	Up
ZBTB18	− 1.426169	0.000933	Down
TOX	1.381994	0.000938	Up
DACT1	1.205543	0.000943	Up
CASK	− 1.472338	0.000958	Down
KITLG	1.267156	0.000963	Up
KMT2A	− 1.28931	0.000968	Down
TRBV6-8	− 1.402432	0.000973	Down
PARP6	− 1.228703	0.000983	Down
CLMP	1.252655	0.001007	Up
EGR3	− 1.348087	0.001017	Down
PCDHB1	− 1.305233	0.001022	Down
TC10000913.hg.1	− 1.441636	0.001037	Down
TC01001567.hg.1	− 1.297173	0.001066	Down
SLC16A2	1.240464	0.001081	Up
COLEC12	1.203956	0.001096	Up
PAPD5	− 1.251764	0.001101	Down
GABRA3	1.355514	0.001116	Up
LPCAT3	1.334572	0.001126	Up
FAM102B	− 1.249464	0.00113	Down
CALR	1.210971	0.00115	Up
CNTFR	1.207485	0.00116	Up
HUWE1	− 1.286372	0.001165	Down
LOXL1	1.3066	0.00117	Up
JHDM1D	− 1.223213	0.00119	Down
HPRT1	− 1.395397	0.001209	Down
HOXC8	1.24955	0.001214	Up
DOCK11	− 1.266513	0.001234	Down
R3HDM2	− 1.226605	0.001278	Down
TRAM1L1	1.237032	0.001283	Up
POLA1	− 1.316949	0.001288	Down
VPS35	− 1.217861	0.001298	Down
CALU	− 1.289373	0.001323	Down
CD248	1.327827	0.001347	Up
THOC2	− 1.388434	0.001377	Down
UCP2	1.214025	0.001387	Up
ETV6	1.275435	0.001396	Up
AP2S1	1.218648	0.001411	Up
PIAS1	− 1.266045	0.001475	Down
TFAP2A	− 1.207326	0.00149	Down
IGFBP4	1.208644	0.0015	Up
DDIT3	− 1.300464	0.001515	Down
PRR9	− 1.306757	0.001529	Down
LRP1	1.262418	0.001539	Up
EPHA7	1.222492	0.001549	Up
GATM	1.233004	0.001594	Up
MSN	− 1.260531	0.001598	Down
P4HA3	1.253025	0.001623	Up
TAF10	1.23132	0.001628	Up
PDE7B	1.265724	0.001643	Up
KLHL13	1.21332	0.001658	Up
ATP11C	− 1.236782	0.001746	Down
HIST1H4L	1.225746	0.001761	Up
JMJD1C	− 1.223153	0.001766	Down
WNK1	− 1.347429	0.001791	Down
ATP6AP2	− 1.265769	0.001815	Down
TRBV10-1	− 1.203962	0.001855	Down
SRPX	1.225977	0.001864	Up
FGF7	1.217304	0.001909	Up
TMTC2	1.228712	0.001914	Up
MYO1D	1.22793	0.001948	Up
SARS	− 1.329997	0.001968	Down
STRBP	− 1.209603	0.002002	Down
SOX4	1.201564	0.002017	Up
EGR2	− 1.203287	0.002022	Down
LCE1D	− 1.216676	0.002047	Down
MED14	− 1.219439	0.002076	Down
OR10P1	− 1.236803	0.002086	Down
EXT1	1.244359	0.002091	Up
SOCS3	1.34295	0.002101	Up
CPSF6	− 1.214996	0.00216	Down
PKM	1.208686	0.00218	Up
FOSB	− 1.203628	0.002293	Down
TRAPPC1	1.301019	0.002318	Up
SPRR2B	− 1.274955	0.002323	Down
EMX2	1.278187	0.002396	Up
TOP1	1.225449	0.002401	Up
TBC1D19	− 1.224248	0.002406	Down
PTCHD1	− 1.206554	0.002431	Down
CDH2	1.221089	0.002441	Up
SPTLC2	1.254554	0.00252	Up
PCBP2	− 1.289738	0.002534	Down
IGKV1D-8	− 1.227643	0.002549	Down
TC03000927.hg.1	− 1.274058	0.002569	Down
TC13000014.hg.1	− 1.205937	0.002633	Down
BRAF	− 1.234126	0.002648	Down
CNOT6L	− 1.272615	0.002658	Down
PAN3	− 1.295252	0.002707	Down
SETBP1	1.224547	0.002712	Up
SPRR1A	− 1.236736	0.002717	Down
TXLNG	− 1.205245	0.002726	Down
TSC22D2	− 1.307114	0.002761	Down
ATF3	− 1.205287	0.002776	Down
FUBP1	− 1.224805	0.002781	Down
DNAJC30	1.22357	0.002825	Up
RCN3	1.218967	0.002879	Up
AGFG1	1.246198	0.002884	Up
HNRNPA3	1.23159	0.002919	Up
SRPK2	− 1.200287	0.002943	Down
ATRX	− 1.226812	0.002978	Down
ADAMTS6	− 1.248056	0.002983	Down
RAB1A	1.34731	0.003022	Up
IGKV3− 11	− 1.238746	0.003121	Down
OR4C6	1.227941	0.003145	Up
XBP1	1.202697	0.00316	Up
MURC	− 1.350099	0.003189	Down
SNX12	− 1.22367	0.003209	Down
TC11000975.hg.1	− 1.332179	0.003239	Down
HOXA5	1.302985	0.003268	Up
RBP1	1.216422	0.003396	Up
TC19000375.hg.1	− 1.216419	0.003406	Down
KDM6A	− 1.327135	0.003505	Down
TC06001136.hg.1	− 1.438272	0.003628	Down
CAV1	− 1.206156	0.003643	Down
ACSL4	1.257627	0.003682	Up
LRP6	1.211757	0.00379	Up
HIST1H1B	1.245633	0.004096	Up
DEFB136	− 1.244558	0.00415	Down
FBXO33	1.239768	0.004229	Up
TANC2	− 1.233366	0.004303	Down
USP12	1.334372	0.004475	Up
OR5A2	− 1.287342	0.004539	Down
TC18000270.hg.1	1.23255	0.004657	Up
OR51L1	− 1.224446	0.004948	Down
OR51B6	− 1.256007	0.004992	Down
PTEN	1.249442	0.005116	Up
TMSB10	1.251421	0.005327	Up
VEZF1	− 1.29591	0.005436	Down
NFIB	1.220599	0.0055	Up
CCDC166	1.217336	0.005529	Up
STAG2	− 1.224684	0.005933	Down
PIAS2	− 1.200256	0.00648	Down
ZSWIM6	1.222542	0.00651	Up
ZNF281	1.209366	0.006697	Up
SYT11	1.201281	0.00682	Up
SCXA	− 1.222753	0.007815	Down
ALX1	− 1.230342	0.007992	Down
EFNA5	1.202225	0.008702	Up
MGC15705	1.234392	0.009229	Up
GPR174	1.246926	0.009317	Up
TC09000877.hg.1	− 1.325127	0.010825	Down
KRTAP19-4	1.262139	0.010972	Up
OR52N2	1.201364	0.011977	Up

Gene symbol	Fold change	p-value	Gene feature
(B) *Genes up/down-regulated in non-purified lung TCs*^*SV40*^ *compared with purified primary lung TCs*			
COL1A1	2.030789	5.20E−05	Up
COL1A2	1.647326	5.70E−05	Up
SYVN1	1.520112	6.20E−05	Up
COL12A1	1.390303	6.60E−05	Up
MYH10	1.517922	7.10E−05	Up
ACTA1	1.453697	7.60E−05	Up
SLIT3	1.501818	8.10E−05	Up
COL3A1	1.756169	8.60E−05	Up
FBN1	1.641521	9.10E−05	Up
COL5A2	1.737694	9.60E−05	Up
GATA6	1.796826	0.000101	Up
IGFBP7	1.641817	0.000106	Up
SEMA3D	1.437537	0.000111	Up
ANGPTL7	1.811625	0.000116	Up
MEIS2	1.53255	0.000121	Up
THBS1	1.678925	0.000125	Up
HS6ST2	1.220779	0.00013	Up
BICC1	1.50068	0.000135	Up
MED12	− 1.428542	0.00014	Down
HSPA5	1.887927	0.000145	Up
PFN1	1.558653	0.00015	Up
PPARGC1A	− 1.432059	0.000155	Down
NFIB	1.535713	0.00016	Up
RBPJ	− 1.376913	0.000165	Down
ACTN1	1.37672	0.00017	Up
EXT1	1.57285	0.000175	Up
TNC	1.293769	0.00018	Up
PCDH7	1.762644	0.000185	Up
TXNIP	1.299338	0.000189	Up
LPCAT3	1.43629	0.000194	Up
COL4A5	1.312681	0.000199	Up
NUAK1	1.412387	0.000204	Up
ATP1A1	1.303558	0.000209	Up
WT1	1.363849	0.000214	Up
MASP1	− 1.206501	0.000219	Down
VCL	1.617321	0.000224	Up
IER5L	1.262549	0.000229	Up
MYO1D	1.463161	0.000234	Up
PLOD2	1.269697	0.000239	Up
FOXP2	1.290662	0.000244	Up
ATP1B1	1.344335	0.000248	Up
KRTAP29-1	− 1.332419	0.000253	Down
ITGAV	1.294132	0.000258	Up
CPXM1	1.343008	0.000263	Up
FST	1.55845	0.000268	Up
CTGF	1.578183	0.000273	Up
HMGCS1	1.228293	0.000278	Up
PLXDC2	1.295572	0.000288	Up
LOXL1	1.332093	0.000293	Up
TM9SF4	1.351928	0.000298	Up
GGCX	1.265383	0.000303	Up
INPPL1	1.32131	0.000308	Up
DUSP6	1.275474	0.000312	Up
NFIA	1.675759	0.000317	Up
GRIA3	1.592221	0.000322	Up
LTBP1	− 1.275386	0.000332	Down
BNC1	1.391268	0.000337	Up
FEZ1	− 1.275203	0.000342	Down
OGT	− 1.599369	0.000347	Down
NXN	1.203178	0.000352	Up
HIST1H2BM	1.309386	0.000357	Up
APH1A	1.273537	0.000362	Up
TMEM259	1.230715	0.000371	Up
THBS2	1.232906	0.000376	Up
ANXA6	1.256028	0.000381	Up
CDH2	1.2585	0.000391	Up
SEC23A	1.343492	0.000401	Up
NNAT	1.433848	0.000406	Up
EMX2	1.295739	0.000411	Up
VCAM1	1.215258	0.000416	Up
DNAJC1	1.413451	0.000421	Up
PCDH19	− 1.490493	0.000426	Down
ILDR2	1.412592	0.000431	Up
DOCK3	− 1.218811	0.000445	Down
DEFB128	− 1.253647	0.00045	Down
ERO1L	1.201323	0.00046	Up
CRYBA1	− 1.209259	0.000465	Down
CELF2	1.399193	0.000475	Up
ZNF503	1.316826	0.00048	Up
TM9SF3	1.337447	0.000485	Up
PTPRD	1.337777	0.000494	Up
IGHV3-43	− 1.352419	0.000499	Down
KIAA1199	1.358808	0.000509	Up
NPR2	− 1.233749	0.000514	Down
SLC7A2	1.219152	0.000519	Up
TRBV21OR9-2	− 1.26885	0.000524	Down
TCF7L2	1.344321	0.000534	Up
SRPX	1.275048	0.000549	Up
TUBG1	1.240509	0.000554	Up
TNFAIP1	1.207721	0.000558	Up
KCNAB1	1.21542	0.000563	Up
ARF6	1.224098	0.000568	Up
FBXO33	1.218704	0.000573	Up
PRPF19	1.25947	0.000578	Up
RAPH1	1.350071	0.000583	Up
PDIA3	1.527168	0.000593	Up
PBX3	1.328363	0.000598	Up
SEC23B	1.264846	0.000603	Up
GABRA3	1.405001	0.000608	Up
ARHGAP1	1.548495	0.000617	Up
AP3S1	1.293716	0.000622	Up
NFIX	1.300317	0.000627	Up
SPTLC2	1.34066	0.000632	Up
HOXC6	1.280515	0.000637	Up
RAB11B	1.318414	0.000642	Up
FHL1	1.206581	0.000652	Up
LMO4	1.222458	0.000672	Up
GBF1	1.291975	0.000677	Up
MBD6	− 1.274947	0.000681	Down
STAT3	1.346345	0.000686	Up
TLN1	1.318005	0.000691	Up
KIDINS220	1.20164	0.000696	Up
ETV6	1.300092	0.000701	Up
ATP2A2	1.245839	0.000706	Up
PARVA	1.340702	0.000711	Up
OR10D3	− 1.379974	0.000716	Down
ILK	1.411794	0.000721	Up
PLCE1	1.207815	0.000731	Up
SERPINH1	1.307929	0.000741	Up
HIF1A	1.377218	0.000745	Up
PDIA4	1.235338	0.000755	Up
MAP4K4	1.281043	0.00076	Up
REXO2	1.383088	0.000765	Up
SLC6A17	− 1.217838	0.00077	Down
PCDH17	− 1.632353	0.000775	Down
EFNA5	1.543491	0.00078	Up
LCE3E	− 1.318245	0.000785	Down
LOX	1.263793	0.00079	Up
FERMT2	1.235564	0.000795	Up
NEO1	1.379359	8.00E-04	Up
TENM3	1.236228	0.000819	Up
CDH11	1.294755	0.000824	Up
KLHDC3	1.275726	0.000829	Up
HMGA1	− 1.21677	0.000834	Down
SCD	1.277895	0.000839	Up
CCND2	− 1.222991	0.000844	Down
HES1	1.308538	0.000849	Up
PDE7B	1.38073	0.000854	Up
SPARC	1.349498	0.000864	Up
SMAD6	1.280817	0.000878	Up
SARS	− 1.260078	0.000883	Down
OSBP	1.345413	0.000903	Up
ACTR1A	1.256685	0.000913	Up
RUNX1T1	− 1.252339	0.000918	Down
TFAP2A	− 1.255715	0.000927	Down
NRG1	1.200557	0.000937	Up
HOXB5	− 1.295109	0.000942	Down
HECTD1	1.351927	0.000947	Up
LCE1C	− 1.270503	0.000952	Down
SPRED1	1.241191	0.000957	Up
PLAG1	− 1.268008	0.000967	Down
EFNB2	1.525576	0.000972	Up
HIPK3	1.37177	0.000977	Up
ID1	1.349752	0.000982	Up
GRIA4	1.321472	0.000991	Up
FAM168A	1.339626	0.001001	Up
FAM98A	1.331744	0.001006	Up
DERL2	1.375325	0.001026	Up
HSPA4	1.279904	0.001031	Up
ABCA1	1.213015	0.001036	Up
GATM	1.418597	0.001041	Up
SLC7A11	1.218849	0.001046	Up
RAB6A	1.254262	0.00105	Up
IGF1	1.369249	0.00107	Up
CCDC80	1.219878	0.00108	Up
KMT2D	− 1.206701	0.001085	Down
HOXC4	1.445692	0.00109	Up
PPP3CA	1.37054	0.001095	Up
HSPA1A	1.212978	0.0011	Up
BMPR2	1.217688	0.001105	Up
ACTG1	1.229217	0.00111	Up
ZDHHC5	1.288505	0.001114	Up
CNIH	1.285039	0.001124	Up
UNC5B	1.204984	0.001139	Up
CAPN6	− 1.418505	0.001149	Down
ZFP36L1	1.284405	0.001154	Up
FGF18	1.238166	0.001159	Up
RAB1A	1.541258	0.001164	Up
FMR1	1.333774	0.001208	Up
FAM188A	1.405263	0.001213	Up
FAM168B	1.275522	0.001228	Up
ITGB5	1.204294	0.001242	Up
KRTAP5-2	− 1.219252	0.001252	Down
FBLN5	1.287407	0.001262	Up
ACVR2A	1.403689	0.001277	Up
ADAMTS9	1.226553	0.001292	Up
NETO1	1.219365	0.001301	Up
ABI1	1.232061	0.001306	Up
INSL6	1.241028	0.001321	Up
TJP1	1.229587	0.001326	Up
HNRNPUL2	1.282031	0.001331	Up
COPG1	1.228987	0.001346	Up
COPB1	1.259033	0.001351	Up
DIO3	1.28299	0.001356	Up
DYNLL2	1.311492	0.00136	Up
DDX26B	− 1.257624	0.001365	Down
SLIT2	1.236976	0.00138	Up
GPC3	1.242766	0.001385	Up
PYGB	1.210405	0.001395	Up
MSANTD2	− 1.247787	0.001405	Down
CYGB	1.321169	0.00141	Up
GPC6	1.254278	0.001415	Up
TC08000204.hg.1	− 1.443858	0.001424	Down
STK39	1.37139	0.001429	Up
RAB1B	1.242841	0.001439	Up
ELAVL2	− 1.223928	0.001449	Down
HSP90B1	1.375708	0.001474	Up
ACTN4	1.314084	0.001483	Up
HSPA1A	1.215746	0.001503	Up
AMMECR1L	1.245785	0.001508	Up
RND3	1.239337	0.001518	Up
SPRY2	1.233483	0.001523	Up
DPYSL2	1.236003	0.001528	Up
DDB1	1.27575	0.001552	Up
SMARCA2	1.262741	0.001567	Up
HDGFRP3	1.330639	0.001572	Up
SULF1	1.221198	0.001577	Up
STK19	− 1.210825	0.001594	Down
STK19	− 1.210825	0.001594	Down
UPF2	1.267351	0.001606	Up
TRBV4-1	− 1.237852	0.001621	Down
APP	1.206738	0.001636	Up
AFF4	1.209778	0.001641	Up
MEF2A	1.213845	0.001651	Up
EDEM3	1.261632	0.001661	Up
HSPA1A	1.214617	0.001666	Up
GABRA4	1.24602	0.001675	Up
SPTAN1	1.260395	0.001685	Up
TEAD1	1.355621	0.001695	Up
WNK1	− 1.539904	0.001705	Down
LRP6	1.290518	0.00171	Up
PRRX1	1.233827	0.001749	Up
HSPA1A	1.213076	0.001759	Up
RAB8A	1.200712	0.001769	Up
STIM1	1.32461	0.001779	Up
ERLEC1	1.216094	0.001793	Up
ADRB1	1.24843	0.001803	Up
TMSB10	1.475064	0.001808	Up
ALCAM	1.207847	0.001857	Up
CYP51A1	1.220803	0.001862	Up
EGR3	− 1.357705	0.001877	Down
CDC27	1.318143	0.001887	Up
PANK3	1.23265	0.001921	Up
HOXA2	1.488536	0.001951	Up
TRBV6-8	− 1.340511	0.001971	Down
CRIM1	1.207809	0.001975	Up
LMAN1	1.205915	0.00198	Up
DEFB113	− 1.249904	0.001985	Down
FN1	1.284284	0.00199	Up
PRKG1	1.239048	0.00202	Up
TC15001075.hg.1	− 1.244805	0.002025	Down
HIST1H2BJ	1.336659	0.002074	Up
TMEM57	1.269164	0.002094	Up
USP12	1.314548	0.002118	Up
CAMK2N2	1.20406	0.002143	Up
TMTC2	1.200876	0.002162	Up
ANAPC1	1.257636	0.002167	Up
C1QL3	1.224547	0.002236	Up
KPRP	− 1.284699	0.002246	Down
PAPOLA	1.313258	0.002295	Up
ACTC1	1.2396	0.002305	Up
LCE2A	− 1.308229	0.00232	Down
CHD4	1.255055	0.002349	Up
EIF5	1.234533	0.002354	Up
PCDH10	1.328826	0.002379	Up
MYL9	1.277676	0.002404	Up
MUT	− 1.247925	0.002413	Down
MEIS1	1.223628	0.002423	Up
P2RX3	− 1.225147	0.002438	Down
UBE2M	1.345125	0.002458	Up
BMPR1A	1.384032	0.002482	Up
RAB39B	− 1.28009	0.002507	Down
MBNL2	1.267238	0.002556	Up
PLK1	1.222337	0.002561	Up
TRGV8	− 1.279773	0.002586	Down
CNN1	1.208197	0.00261	Up
ZSWIM6	1.291355	0.00265	Up
EFNB1	1.235229	0.002679	Up
MAN1A1	1.233248	0.002694	Up
HOXC8	1.264716	0.002714	Up
SMAD7	1.275196	0.002763	Up
TRIP12	1.322101	0.002778	Up
ATP2B1	1.212402	0.002812	Up
MEX3C	1.246507	0.002822	Up
SEC24A	1.225516	0.002827	Up
PSMC4	1.238673	0.002837	Up
KRTAP22-2	− 1.253194	0.002846	Down
LOC100509638	− 1.331709	0.002861	Down
KDM2A	1.21736	0.00292	Up
EIF4G2	1.346528	0.002945	Up
NOXRED1	− 1.247281	0.002964	Down
TC02002617.hg.1	− 1.384833	0.002989	Down
STT3A	1.269875	0.003009	Up
TC11000975.hg.1	− 1.273558	0.003058	Down
APOBEC1	− 1.247386	0.003063	Down
PPP2R5E	1.279695	0.003078	Up
FZD8	1.347838	0.003147	Up
HIST1H2BH	1.399643	0.003176	Up
AP1G1	1.251832	0.003191	Up
C11orf73	1.210719	0.003215	Up
RTN3	1.21898	0.00323	Up
LCE1E	− 1.222095	0.003255	Down
ADAM9	1.210498	0.00326	Up
CLINT1	1.294737	0.003294	Up
TRGV3	− 1.208645	0.003329	Down
P4HA3	1.227186	0.003388	Up
CELF1	1.215676	0.003417	Up
MAP4K5	1.258241	0.003422	Up
TARDBP	− 1.229146	0.003486	Down
BMP4	1.239271	0.003511	Up
ACTA2	1.255606	0.003525	Up
SF3B3	1.20972	0.003614	Up
DCUN1D5	1.248267	0.003634	Up
OLFML2B	1.220926	0.003643	Up
TOP1	1.254134	0.003663	Up
BTG1	− 1.348061	0.003673	Down
CALR	1.300821	0.003703	Up
H3F3A	1.228003	0.003742	Up
PUM1	1.232872	0.00383	Up
PSMA3	1.227334	0.003845	Up
OR2H2	− 1.261057	0.003865	Down
FAR1	1.284889	0.003875	Up
RC3H2	1.209665	0.003894	Up
IGKV1D-37	1.290533	0.003899	Up
EIF4B	− 1.253518	0.003949	Down
ROMO1	1.212986	0.004017	Up
NCKAP1	1.279797	0.004067	Up
HSPA2	1.241027	0.004086	Up
CPD	1.254726	0.004091	Up
NPR3	1.205107	0.004131	Up
RAP1B	1.300105	0.004165	Up
SEC62	1.322724	0.00419	Up
IGLJ4	1.46599	0.004263	Up
OR5A2	− 1.612969	0.004332	Down
IGLV6-57	1.202727	0.004342	Up
RAB21	1.224355	0.004367	Up
MMP14	1.259391	0.004465	Up
LELP1	− 1.280949	0.004475	Down
ZNF281	1.341512	0.004495	Up
KRTAP12-3	− 1.222558	0.004657	Down
MMD	1.36424	0.004691	Up
TIMP3	1.219996	0.004711	Up
ACTL8	− 1.248052	0.004746	Down
HIST1H2BE	1.383938	0.004829	Up
POLA1	− 1.226576	0.004839	Down
AIDA	1.221832	0.004854	Up
OR2H2	− 1.251288	0.004859	Down
PMP22	1.201201	0.004923	Up
KRTAP12-3	− 1.215537	0.004938	Down
SLC36A4	1.206965	0.004962	Up
SHOC2	1.224107	0.004972	Up
SOX4	1.200056	0.004977	Up
ISM1	1.253651	0.005031	Up
CSRP2	1.210735	0.005041	Up
OR4D11	− 1.307921	0.005046	Down
CAPRIN1	1.203686	0.005095	Up
PPP3R1	1.237317	0.005208	Up
PPIC	1.224048	0.005238	Up
PRAMEF10	− 1.247763	0.005257	Down
MBTPS2	− 1.210053	0.00542	Down
UBTD2	1.203269	0.00543	Up
SEC11A	1.208371	0.005449	Up
USP25	1.213603	0.005498	Up
PTMA	1.217797	0.005513	Up
FKBP1C	1.209098	0.005523	Up
ANKIB1	1.23614	0.005651	Up
GSPT1	1.233347	0.005715	Up
UCP2	1.209526	0.005735	Up
TGFB3	1.220573	0.005769	Up
HIST1H4G	1.221559	0.005803	Up
IGLJ5	− 1.226509	0.005818	Down
KIF5B	1.306336	0.005897	Up
TC10000913.hg.1	− 1.233065	0.006202	Down
TC03000927.hg.1	− 1.285309	0.006207	Down
PGRMC1	1.230418	0.006286	Up
HOXC5	1.236194	0.006291	Up
KRTAP13-4	1.202792	0.006315	Up
OR3A2	− 1.250913	0.006384	Down
SRSF6	1.240448	0.006389	Up
GTSF1L	− 1.204976	0.006532	Down
SYNCRIP	1.229937	0.006571	Up
HIST1H2BO	1.347018	0.006699	Up
EPC2	1.212558	0.006748	Up
CTDSPL2	1.229495	0.006768	Up
OR10A3	− 1.206131	0.006817	Down
KRTAP7-1	− 1.297287	0.006896	Down
THOC2	− 1.220789	0.006979	Down
ETF1	1.225052	0.006984	Up
TC11000974.hg.1	− 1.273131	0.006994	Down
PRELID1	1.210411	0.007014	Up
IGHV3-38	− 1.267673	0.007029	Down
OR1L3	− 1.275722	0.007034	Down
SRP54	1.294575	0.007117	Up
EXOC5	1.217832	0.007122	Up
OR2H2	− 1.263243	0.007292	Down
OR2H2	− 1.263243	0.007292	Down
RNF122	1.27436	0.007373	Up
PTBP2	− 1.249936	0.007644	Down
ERP44	1.208521	0.007683	Up
CCR7	− 1.217568	0.007747	Down
QKI	1.209051	0.008013	Up
ZFP42	− 1.215171	0.008141	Down
IGLV3-22	− 1.215548	0.008195	Down
DEFB103A	− 1.213104	0.008451	Down
OR3A3	− 1.258226	0.008495	Down
TOP2B	1.206659	0.008697	Up
PTEN	1.221197	0.008785	Up
IGHV3-66	− 1.226136	0.008839	Down
PPP6C	1.210188	0.008908	Up
PRAMEF8	− 1.238928	0.008923	Down
STRN3	1.291297	0.008948	Up
TC01003841.hg.1	− 1.354102	0.008997	Down
TC05001047.hg.1	− 1.208467	0.009046	Down
MAGT1	1.21942	0.00909	Up
OR2H2	− 1.252699	0.009179	Down
OR2H2	− 1.252699	0.009179	Down
OR2H2	− 1.252699	0.009179	Down
RPL4	− 1.201599	0.009336	Down
OSTC	1.203947	0.009622	Up
IKZF5	1.216696	0.009868	Up
KRTAP10-11	− 1.205771	0.009912	Down
NLK	1.209405	0.009932	Up
OR2W1	1.274304	0.009978	Up
OR2W1	1.274304	0.009978	Up
OR2W1	1.274304	0.009978	Up
OR2W1	1.274304	0.009978	Up
OR2W1	1.274304	0.009978	Up
OR2W1	1.274304	0.009978	Up
OR2W1	1.274304	0.009978	Up
OR2W1	1.274304	0.009978	Up
CD248	1.239436	0.010758	Up
HIST1H4F	1.204154	0.010876	Up
OR6C1	− 1.216993	0.010911	Down
TMEM202	− 1.213705	0.011093	Down
TP53INP1	− 1.205295	0.011329	Down
KPNA3	1.21541	0.011511	Up
C7orf66	− 1.226557	0.011919	Down
TMSB4XP4	1.260484	0.011964	Up
TRAV24	− 1.229097	0.012215	Down
WDR26	1.201142	0.012239	Up
KRTAP9-3	− 1.229216	0.012293	Down
CSNK1A1L	1.212513	0.012716	Up
TC08001202.hg.1	− 1.327872	0.013076	Down
TAS2R16	− 1.212834	0.013287	Down
IGHV1-8	1.229339	0.013312	Up
RAB2A	1.248861	0.013563	Up
GPRIN2	− 1.234909	0.014257	Down
DEFB118	− 1.270079	0.014778	Down
TC07000141.hg.1	− 1.33934	0.015	Down
TC02000056.hg.1	− 1.236353	0.015231	Down
LCE1D	− 1.234952	0.015432	Down
FAM218A	1.265433	0.015487	Up
LCE2B	− 1.208788	0.015511	Down
CRABP1	− 1.271706	0.015536	Down
OR1F1	− 1.231414	0.015841	Down
LOC100144595	− 1.207195	0.016023	Down
GALNT1	1.247945	0.016274	Up
LEP	− 1.236716	0.016746	Down
DEFB136	− 1.203294	0.017223	Down
TC07000218.hg.1	− 1.256974	0.017302	Down
PPIAL4E	1.263803	0.02144	Up
USP17	1.21594	0.026188	Up

Gene symbol	Fold change	p-value	Gene feature
(C) *Genes up/down-regulated in non-purified lung TCs*^*SV40*^ *compared with non-purified primary lung TCs*			
HSPA5	2.28735	5.20E−05	Up
BNC1	1.464673	5.70E−05	Up
CAPN6	− 1.711607	6.20E−05	Down
HOXC6	1.334235	6.60E−05	Up
COL12A1	1.349599	7.10E−05	Up
IGFBP7	1.60287	7.60E−05	Up
GATA6	1.812957	8.10E−05	Up
SLIT3	1.453596	8.60E−05	Up
ANGPTL7	1.780209	9.10E−05	Up
COL5A2	1.723719	9.60E−05	Up
COL1A1	1.699369	0.000101	Up
PPARGC1A	− 1.461747	0.000106	Down
MEIS2	1.482364	0.000111	Up
SYVN1	1.426147	0.000116	Up
MYH10	1.457796	0.000121	Up
MED12	− 1.423327	0.000126	Down
SMO	− 1.306238	0.00013	Down
DNM1	− 1.263174	0.000135	Down
ERO1L	1.270922	0.00014	Up
PBX1	− 1.307252	0.000145	Down
THBS1	1.469591	0.00015	Up
FBN1	1.535712	0.000155	Up
TRBV21OR9-2	− 1.373795	0.00016	Down
PTPRD	1.295981	0.000165	Up
BICC1	1.467091	0.00017	Up
DUSP6	1.245629	0.000175	Up
TXNIP	1.314454	0.00018	Up
FOXP2	1.294122	0.000185	Up
COL1A2	1.44837	0.00019	Up
ACTA1	1.287627	0.000195	Up
SEMA3A	− 1.310532	0.000199	Down
DES	1.335159	0.000204	Up
REXO2	1.380366	0.000209	Up
ACTN1	1.352297	0.000214	Up
VCL	1.66324	0.000219	Up
PCDH19	− 1.617275	0.000224	Down
SLC6A8	− 1.239617	0.000229	Down
PCDH17	− 1.932881	0.000239	Down
OGT	− 1.657267	0.000244	Down
HOXB5	− 1.442975	0.000249	Down
SEMA3D	1.383364	0.000254	Up
MECOM	− 1.327861	0.000259	Down
WT1	1.349575	0.000263	Up
KCNAB1	1.210026	0.000268	Up
RUNX1T1	− 1.360214	0.000273	Down
PLCE1	1.209156	0.000278	Up
COL3A1	1.499792	0.000283	Up
FAM102B	− 1.353459	0.000288	Down
MBD6	− 1.351938	0.000293	Down
CRYBA1	− 1.269111	0.000298	Down
ANXA6	1.224881	0.000303	Up
PCDH7	1.650618	0.000308	Up
PLXDC2	1.3089	0.000313	Up
FST	1.553608	0.000318	Up
NUAK1	1.27549	0.000323	Up
COL4A5	1.276706	0.000327	Up
IGF1	1.254137	0.000332	Up
SEC23A	1.389357	0.000337	Up
ATP1B1	1.276224	0.000342	Up
HS6ST2	1.230549	0.000347	Up
ZNF462	1.218491	0.000352	Up
FAM218A	1.448595	0.000357	Up
SEC23B	1.258266	0.000362	Up
PLOD2	1.25462	0.000367	Up
DNAJC1	1.413719	0.000372	Up
RAPH1	1.369576	0.000377	Up
GRIA3	1.691491	0.000382	Up
PSMC4	1.341278	0.000387	Up
SEC24A	1.317102	0.000391	Up
TGFB1I1	1.214649	0.000396	Up
ELAVL2	− 1.263137	0.000401	Down
NRP2	− 1.307128	0.000406	Down
KIAA1199	1.371075	0.000411	Up
LPHN3	− 1.212789	0.000421	Down
ARRDC3	− 1.350686	0.000426	Down
KLHL13	1.212748	0.000431	Up
MYO1D	1.385837	0.000436	Up
TM9SF3	1.307262	0.000441	Up
TFAP2A	− 1.274332	0.000446	Down
ARL6IP1	1.244781	0.000451	Up
CPXM1	1.300338	0.000455	Up
CCND2	− 1.241703	0.00046	Down
SPTLC2	1.354194	0.000465	Up
NNAT	1.392895	0.00047	Up
STK39	1.540497	0.000475	Up
ITGAV	1.267551	0.00048	Up
LTBP1	− 1.280112	0.000485	Down
CELF4	1.232625	0.00049	Up
OSBP	1.424925	0.000495	Up
MASP1	− 1.219414	5.00E−04	Down
FRMD5	− 1.36697	0.000505	Down
AP3S1	1.258065	0.00051	Up
BNC2	− 1.294519	0.000515	Down
TRPS1	− 1.34803	0.000519	Down
TUBG2	1.223767	0.000529	Up
CNN1	1.210395	0.000544	Up
TNC	1.281125	0.000549	Up
GBF1	1.314328	0.000554	Up
NFAT5	− 1.519776	0.000559	Down
NFIA	1.498716	0.000564	Up
ADAM22	1.202425	0.000584	Up
RBPJ	− 1.246466	0.000588	Down
EGR1	− 1.356633	0.000593	Down
ILDR2	1.458561	0.000603	Up
FSCN1	1.213951	0.000608	Up
VGLL3	1.201156	0.000623	Up
HIF1A	1.377011	0.000633	Up
ATP2A2	1.25653	0.000638	Up
ARF6	1.256706	0.000648	Up
NETO1	1.206084	0.000662	Up
PLK1	1.239207	0.000667	Up
CAV1	− 1.227122	0.000682	Down
ATF6	1.278798	0.000697	Up
ATP1A1	1.260299	0.000702	Up
GPC3	1.252397	0.000707	Up
HSPA4	1.310919	0.000716	Up
MAP4K4	1.293954	0.000721	Up
HOXC4	1.528822	0.000736	Up
JAG1	1.203931	0.000746	Up
INPPL1	1.248465	0.000756	Up
FOSB	− 1.204723	0.000766	Down
KMT2D	− 1.228557	0.000771	Down
PRPF19	1.227842	0.000776	Up
CCDC80	1.225889	0.00079	Up
TM9SF4	1.256719	0.000805	Up
NXN	1.219999	0.00081	Up
COPB1	1.27883	0.00082	Up
SRPX	1.337622	0.000825	Up
SMAD7	1.390506	0.00083	Up
VPS25	1.267452	0.000835	Up
TC18000270.hg.1	1.241506	0.000844	Up
DDX26B	− 1.386832	0.000854	Down
SCD	1.222675	0.000859	Up
KMT2A	− 1.233077	0.000869	Down
JUNB	− 1.289174	0.000874	Down
OR10P1	− 1.222678	0.000884	Down
LPCAT3	1.33701	0.000894	Up
ARHGAP1	1.390092	0.000909	Up
GRIA4	1.313269	0.000928	Up
GGCX	1.210524	0.000948	Up
CNIH	1.326625	0.000958	Up
SPARC	1.31356	0.000963	Up
S100A2	− 1.206393	0.000968	Down
THBS2	1.201097	0.000973	Up
MEF2A	1.231751	0.000977	Up
HOXC8	1.40158	0.000987	Up
SARS	− 1.216959	0.001002	Down
HOXC4	1.205214	0.001017	Up
FEZ1	− 1.210057	0.001022	Down
HDGFRP3	1.360953	0.001027	Up
SMAD6	1.248102	0.001032	Up
EGR3	− 1.413218	0.001037	Down
LPL	1.241889	0.001046	Up
CREB3L1	1.208352	0.001051	Up
EXT1	1.352789	0.001066	Up
SERPINH1	1.267578	0.001071	Up
CDH11	1.256532	0.001076	Up
FAM126A	− 1.231349	0.001086	Down
ZNF521	− 1.298692	0.00111	Down
PCDH18	− 1.284827	0.00113	Down
ACTN4	1.348564	0.00114	Up
C10orf53	− 1.240495	0.00115	Down
EMX2	1.428467	0.001155	Up
TUBG1	1.240367	0.001169	Up
PLAG1	− 1.253787	0.001194	Down
NEO1	1.311703	0.001209	Up
NR2F2	− 1.327418	0.001214	Down
RAB39B	− 1.208364	0.001229	Down
LCE1C	− 1.227094	0.001234	Down
NPR2	− 1.277907	0.001243	Down
APH1A	1.202275	0.001248	Up
PRR9	− 1.473668	0.001263	Down
FAM168A	1.316093	0.001273	Up
LOX	1.233141	0.001278	Up
ETV6	1.225393	0.001283	Up
MBNL1	− 1.348394	0.001312	Down
ZSWIM6	1.360747	0.001327	Up
GPM6B	− 1.209186	0.001342	Down
FERMT2	1.204761	0.001347	Up
ISM1	1.222367	0.001357	Up
HECTD1	1.325901	0.001366	Up
GPC6	1.249832	0.001371	Up
SPRR1A	− 1.304855	0.001376	Down
C17orf47	− 1.210708	0.001381	Down
POLA1	− 1.278784	0.001396	Down
RNF114	1.215485	0.001406	Up
PHLDB2	1.22064	0.001421	Up
BTG1	− 1.419463	0.001435	Down
OR2H2	− 1.386141	0.00146	Down
RAB6A	1.218989	0.00147	Up
STK19	− 1.226269	0.001482	Down
STK19	− 1.226269	0.001482	Down
EFNB1	1.214243	0.00149	Up
HIPK3	1.272379	0.001514	Up
OR2H2	− 1.336272	0.001524	Down
OR4X2	− 1.231192	0.001539	Down
ZNF503	1.203437	0.001554	Up
ABI1	1.207101	0.001568	Up
FAM188A	1.354332	0.001578	Up
MBNL2	1.315575	0.001593	Up
CDH2	1.263605	0.001618	Up
FAM98A	1.272083	0.001652	Up
DDB1	1.233239	0.001691	Up
TC11000975.hg.1	− 1.370641	0.001696	Down
DEFB128	− 1.229163	0.001701	Down
CYP51A1	1.215873	0.001706	Up
EFNA5	1.525255	0.001711	Up
CLINT1	1.352971	0.001721	Up
TCF4	− 1.350334	0.001736	Down
INTS5	1.272327	0.001741	Up
GATM	1.354506	0.00177	Up
GABRA3	1.32631	0.001775	Up
RAB1A	1.472237	0.001785	Up
TC02001150.hg.1	1.202559	0.001795	Up
CAMK2N2	1.205549	0.0018	Up
HNRNPUL2	1.26108	0.001815	Up
FGF18	1.254064	0.001829	Up
LCE3B	− 1.20559	0.001849	Down
SRPR	1.282511	0.001859	Up
RNF138	1.200216	0.001864	Up
GABRB1	1.20311	0.001869	Up
ZDHHC2	1.231848	0.001879	Up
PTGS2	− 1.235944	0.001888	Down
ANGPT1	− 1.239367	0.001923	Down
CDC27	1.301846	0.001933	Up
ZFP36L2	− 1.309184	0.001957	Down
THOC2	− 1.298345	0.001972	Down
AMMECR1L	1.204913	0.001982	Up
TAGLN	1.21811	0.001997	Up
SPRR1B	− 1.386398	0.002007	Down
ADAMTS6	− 1.251192	0.002021	Down
LCE3E	− 1.238425	0.002036	Down
TEAD1	1.316471	0.00209	Up
OR4D11	− 1.397758	0.002115	Down
PSMD14	1.269457	0.002164	Up
INSL6	1.241962	0.002169	Up
FBXO33	1.240947	0.002174	Up
OR2H2	− 1.335922	0.002211	Down
OR2H2	− 1.335922	0.002211	Down
LCE1D	− 1.24485	0.002233	Down
STIM1	1.291974	0.002253	Up
TRBV6-8	− 1.383088	0.002297	Down
PARVA	1.212507	0.002307	Up
TC11000974.hg.1	− 1.355331	0.002322	Down
CTGF	1.278251	0.002327	Up
PRKG1	1.245661	0.002341	Up
TLN1	1.209426	0.002356	Up
OR3A2	− 1.258635	0.002361	Down
NPR3	1.252907	0.002366	Up
CYGB	1.286596	0.002376	Up
ID1	1.302257	0.002396	Up
HSPA2	1.291744	0.002401	Up
DERL2	1.338707	0.002415	Up
OR2H2	− 1.331198	0.002469	Down
OR2H2	− 1.331198	0.002469	Down
OR2H2	− 1.331198	0.002469	Down
EIF4G2	1.351151	0.002548	Up
PAPOLA	1.311535	0.002578	Up
PPP3R1	1.259841	0.002588	Up
PDIA3	1.321177	0.002597	Up
HSP90B1	1.297674	0.002627	Up
EIF4A1	1.248301	0.002642	Up
TNPO3	− 1.20247	0.002676	Down
UCP2	1.245612	0.002686	Up
EEF1G	1.228255	0.00274	Up
TC13000014.hg.1	− 1.237888	0.002745	Down
TC02002617.hg.1	− 1.519734	0.00275	Down
PFN1	1.248818	0.00276	Up
ERLEC1	1.219854	0.00277	Up
GSPT1	1.296726	0.002878	Up
TC15001723.hg.1	− 1.244628	0.002883	Down
C8orf49	− 1.225571	0.002888	Down
STAT3	1.204904	0.002957	Up
MAN1A1	1.22109	0.002982	Up
ACTR1A	1.210577	0.003046	Up
TAF10	1.234009	0.003075	Up
ONECUT2	− 1.203425	0.0031	Down
PPP2R5E	1.303497	0.003105	Up
STT3A	1.268152	0.003115	Up
TC03001641.hg.1	− 1.243194	0.003134	Down
TGFB3	1.241756	0.003159	Up
DICER1	1.208135	0.003164	Up
LCE2A	− 1.356188	0.003213	Down
HIST1H4J	1.203634	0.003257	Up
P4HA1	1.268543	0.003277	Up
TCF20	− 1.200225	0.003287	Down
HOXC5	1.25077	0.003302	Up
MTX2	1.203709	0.003316	Up
MUT	− 1.206951	0.003376	Down
CPD	1.217165	0.003405	Up
PRELP	− 1.216805	0.00343	Down
OR10D3	− 1.289554	0.003558	Down
EFNB2	1.322603	0.003563	Up
MBTPS2	− 1.219406	0.003577	Down
ACVR2A	1.277435	0.003632	Up
PCDH10	1.289149	0.003641	Up
OR3A3	− 1.294084	0.003676	Down
VAT1L	1.224017	0.003681	Up
ANKIB1	1.250405	0.003705	Up
ATP6AP2	− 1.203066	0.003829	Down
TMSB10	1.33115	0.003893	Up
MYL9	1.238522	0.003976	Up
P4HA3	1.223749	0.004006	Up
LOC100144595	− 1.210771	0.004045	Down
CCDC47	1.254626	0.004085	Up
PTP4A1	1.207393	0.004104	Up
SNX12	− 1.231653	0.004163	Down
TC03000927.hg.1	− 1.34441	0.004173	Down
PRAMEF10	− 1.226358	0.004218	Down
HIST1H2BF	1.201242	0.004331	Up
HIST1H4F	1.278307	0.004355	Up
LSM2	− 1.23126	0.00436	Down
PSMC6	1.28439	0.00437	Up
TMEM57	1.214675	0.00438	Up
TRGV8	− 1.24305	0.004449	Down
SEC11A	1.212858	0.004508	Up
OR51B6	− 1.25219	0.004528	Down
ZNF830	1.223738	0.004552	Up
TMEM238	1.259086	0.004582	Up
TOP1	1.207832	0.004685	Up
LELP1	− 1.254685	0.004764	Down
MMD	1.342077	0.004774	Up
LCE1A	− 1.207411	0.004804	Down
IGKV1D-13	− 1.253243	0.004808	Down
FZD8	1.20833	0.004853	Up
OR5A2	− 1.583233	0.004887	Down
C1QL3	1.202082	0.005015	Up
PANK3	1.209123	0.00506	Up
KIF5B	1.306357	0.005094	Up
SRP54	1.335205	0.005153	Up
ETF1	1.247056	0.005385	Up
HIST1H4G	1.221559	0.005399	Up
TRIP12	1.250366	0.005463	Up
TRAJ59	1.280347	0.005591	Up
DEFB133	− 1.217544	0.00568	Down
HIST1H2BH	1.324812	0.005739	Up
ACTL8	− 1.34524	0.005828	Down
HIST1H2BO	1.331009	0.005867	Up
C16orf87	1.227019	0.005877	Up
HIST1H1B	1.236488	0.005971	Up
PPP3CA	1.2093	0.006	Up
DAZAP2	− 1.246936	0.006069	Down
C7orf66	− 1.257301	0.006168	Down
IRF2BPL	1.222737	0.006197	Up
USP12	1.245254	0.006202	Up
PGAP2	− 1.236121	0.006217	Down
WNK1	− 1.317909	0.006315	Down
FAR1	1.239903	0.006424	Up
HOXA2	1.321485	0.006497	Up
IGHV3-48	− 1.200801	0.006537	Down
LCE2C	− 1.207171	0.006714	Down
PPIC	1.208975	0.006822	Up
COPA	1.213652	0.006847	Up
SPANXN3	1.276398	0.006916	Up
DCUN1D5	1.204299	0.006931	Up
RAP1B	1.238418	0.006946	Up
CMAS	1.312695	0.007064	Up
NLK	1.232188	0.007211	Up
HIST1H2BE	1.335735	0.007216	Up
HRNR	− 1.31544	0.007221	Down
OR4A16	1.208665	0.007285	Up
C8orf48	1.252595	0.007477	Up
CSNK1A1	1.214353	0.007497	Up
UBE2M	1.219256	0.00761	Up
ZNF281	1.250794	0.007817	Up
IGHD2-21	− 1.332138	0.007837	Down
E2F3	1.206829	0.008019	Up
TC10000913.hg.1	− 1.226511	0.008108	Down
NCKAP1	1.22608	0.008285	Up
PPP6C	1.215357	0.008364	Up
SEC62	1.25965	0.008536	Up
EXOC5	1.207899	0.00862	Up
FKBP1C	1.20189	0.008787	Up
ZBTB6	1.223895	0.008964	Up
TRBV6-7	− 1.261291	0.009048	Down
DEFB136	− 1.297342	0.009122	Down
FGF10	− 1.247986	0.009186	Down
OR4C6	1.293432	0.00927	Up
GFPT1	1.20605	0.009309	Up
PSMA6	1.216218	0.009353	Up
TC01002282.hg.1	− 1.202154	0.009477	Down
KRTAP9-8	− 1.222393	0.009486	Down
LCE2B	− 1.251225	0.009595	Down
TC17000686.hg.1	− 1.224599	0.009846	Down
TMSB4XP4	1.276305	0.009935	Up
LOC100509638	− 1.228863	0.010215	Down
OR51L1	− 1.209919	0.010486	Down
TC07000141.hg.1	− 1.389386	0.011299	Down
USP17	1.231161	0.011338	Up
BMPR1A	1.225957	0.011535	Up
IGHV1OR21-1	− 1.207255	0.011811	Down
LOC728819	1.209199	0.011968	Up
WDR26	1.21391	0.012145	Up
TC15001075.hg.1	− 1.206125	0.012165	Down
TRAPPC1	1.207842	0.012264	Up
STRN3	1.251923	0.012589	Up
IGKV1D-37	1.290804	0.012667	Up
TC05000944.hg.1	− 1.209012	0.012855	Down
KRTAP6-3	− 1.209466	0.013017	Down
TC01001567.hg.1	− 1.220919	0.013106	Down
TC08000204.hg.1	− 1.257586	0.013199	Down
TRAV24	− 1.224177	0.013224	Down
KPNA3	1.208042	0.013244	Up
CALHM1	− 1.281085	0.01346	Down
KRTAP5-6	− 1.213396	0.013514	Down
OR5W2	− 1.227139	0.013682	Down
HIST2H2BE	1.203613	0.013928	Up
TAOK1	1.203131	0.014223	Up
RBMXL2	1.242693	0.014371	Up
MGC15705	1.248494	0.014499	Up
NOP58	1.210344	0.014898	Up
TC01003841.hg.1	− 1.272609	0.014947	Down
CASK	− 1.202663	0.015371	Down
GPX5	− 1.231507	0.015435	Down
IGLJ6	− 1.209384	0.015651	Down
OR6C1	− 1.220835	0.015715	Down
OR1L3	− 1.26804	0.015981	Down
IGKV1-9	− 1.205738	0.016425	Down
CSNK1A1L	1.236726	0.016981	Up
OR51A4	− 1.251899	0.017158	Down
TC07000847.hg.1	1.200866	0.017404	Up
TAS2R16	− 1.220859	0.017424	Down
EIF2S2	1.202591	0.018104	Up
RAB2A	1.218567	0.018571	Up
OR56A5	1.204074	0.022471	Up

Gene symbol	Fold change	p-value	Gene feature
(D) *Genes up/down-regulated in purified TCs*^*SV40*^ *compared with non-purified lung TCs*^*SV40*^			
ZBTB18	− 1.554972	0.000298	Down
MID2	− 1.541494	0.000126	Down
HSPA5	− 1.518909	0.000465	Down
TSC22D2	− 1.5017	0.00048	Down
VCL	− 1.495358	0.000204	Down
MEIS2	− 1.460638	8.60E−05	Down
STK39	− 1.423681	0.000313	Down
CTGF	− 1.421299	0.000268	Down
DOCK11	− 1.415118	0.000426	Down
ATF6	− 1.409588	0.000199	Down
SLC7A11	− 1.404048	0.000101	Down
BRAF	− 1.396899	0.000755	Down
NLK	− 1.384761	0.00076	Down
ERO1L	− 1.366172	5.20E−05	Down
HIST1H2BE	− 1.360048	0.003083	Down
MEF2A	− 1.354049	0.000135	Down
CELF1	− 1.34869	0.000706	Down
HPRT1	− 1.348486	0.001415	Down
TBPL1	− 1.344601	0.000573	Down
FAM188A	− 1.344027	0.000652	Down
AZIN1	− 1.322403	0.000288	Down
SYVN1	− 1.309011	0.000145	Down
HIST1H2BO	− 1.308399	0.002104	Down
STRBP	− 1.307068	0.000362	Down
ABCB7	− 1.302327	0.00239	Down
EFNB2	− 1.298157	0.002606	Down
ANKRD1	− 1.293984	0.000455	Down
SLC6A9	− 1.292011	0.000244	Down
RND1	− 1.288509	0.000258	Down
HOXC4	− 1.286961	0.001459	Down
PGRMC1	− 1.285024	0.000775	Down
IGHV1-8	− 1.283244	0.003162	Down
GSPT1	− 1.277447	0.002286	Down
FN1	− 1.272748	0.001287	Down
AKT3	− 1.269844	0.000436	Down
STIM1	− 1.269255	0.00139	Down
EFNA5	− 1.268694	0.002818	Down
CCDC47	− 1.265588	0.001528	Down
DES	− 1.265153	0.00013	Down
PDZD8	− 1.258982	0.000696	Down
GATA6	− 1.253813	0.000637	Down
SEL1L	− 1.25119	0.000603	Down
HDGFRP3	− 1.249545	0.000947	Down
ZNF827	− 1.249169	0.000175	Down
NEO1	− 1.24814	0.001996	Down
MYH10	− 1.247444	0.00049	Down
C16orf87	− 1.247099	0.002311	Down
HES1	− 1.244124	0.000308	Down
HERPUD1	− 1.239348	0.000229	Down
DUSP6	− 1.237186	0.002138	Down
RAPH1	− 1.235005	0.000992	Down
CDC42BPA	− 1.233364	0.00203	Down
ACTN4	− 1.232684	0.00174	Down
ZNF462	− 1.232013	0.000111	Down
VPRBP	− 1.230287	0.00078	Down
RALGAPA1	− 1.229069	0.001804	Down
UPRT	− 1.229057	0.002852	Down
UBE2W	− 1.228348	0.000785	Down
SAMD4B	− 1.227886	0.000667	Down
MTRNR2L4	− 1.224725	0.00172	Down
SEC24A	− 1.224506	0.000421	Down
AMMECR1L	− 1.222428	0.00047	Down
PRKAA2	− 1.221814	0.003029	Down
ACTN1	− 1.218558	0.000623	Down
OSBP	− 1.217315	0.001464	Down
VLDLR	− 1.216913	0.000239	Down
MSI2	− 1.211676	0.000731	Down
MSN	− 1.209973	0.002842	Down
LRRC58	− 1.209927	0.000273	Down
COL4A5	− 1.209539	0.000317	Down
SLAIN2	− 1.207964	0.002094	Down
NDFIP1	− 1.206718	0.001932	Down
PPP2R5B	− 1.20442	0.001134	Down
VPS25	− 1.20254	0.00079	Down
CPD	− 1.201293	0.002626	Down
COLEC12	1.200894	0.00173	Up
THBS3	1.201895	0.000726	Up
KITLG	1.202163	0.000337	Up
SETBP1	1.202182	0.000765	Up
LOXL1	1.202879	0.001203	Up
PRKD1	1.20361	9.10E−05	Up
ZFP36L1	1.203909	0.001755	Up
EDDM3B	1.205244	0.001159	Up
ANGPT1	1.207484	0.001346	Up
MMP2	1.208515	0.000165	Up
FAM170B	1.210029	0.000923	Up
LELP1	1.213262	0.002473	Up
IGFBP4	1.215597	0.002011	Up
STC1	1.217133	0.000928	Up
EPHA7	1.218188	0.000568	Up
PRDM6	1.219262	0.000618	Up
ACTRT1	1.219685	0.001509	Up
XDH	1.222067	0.000209	Up
OR4D11	1.224206	0.000874	Up
HOXA11	1.22653	0.002001	Up
PAK3	1.228895	0.000381	Up
BNC2	1.242835	0.000303	Up
SLC16A2	1.248637	0.000593	Up
NRN1	1.251496	0.000564	Up
GDPD2	1.251704	0.000189	Up
RUNX1T1	1.251964	0.000352	Up
PCDH18	1.252101	0.000263	Up
PCDH9	1.255458	0.001848	Up
MMP16	1.25842	0.000155	Up
PRRX1	1.259453	0.001144	Up
LRP1	1.263799	0.000997	Up
SEMA5A	1.267548	7.10E−05	Up
PBX1	1.270348	0.000185	Up
RDH10	1.271163	6.60E−05	Up
VCAN	1.277158	0.000121	Up
HOXB8	1.284619	0.00016	Up
FOXG1	1.287872	0.001824	Up
MT1G	1.289954	0.000485	Up
SOCS3	1.291472	0.002252	Up
TBX4	1.309269	0.00017	Up
ADAMTS15	1.313557	0.000278	Up
FGF7	1.320284	0.000805	Up
HOXA5	1.322982	0.00144	Up
MME	1.331844	0.00015	Up
DBC1	1.332864	8.10E−05	Up
ZEB2	1.333152	0.000249	Up
LTBP1	1.335297	0.00018	Up
BTG1	1.338416	0.00112	Up
MT1H	1.346673	0.000327	Up
UGCG	1.347608	9.60E−05	Up
TCF4	1.350209	0.000559	Up
MEIS1	1.358188	0.000445	Up
BHLHE22	1.358601	0.00014	Up
MT1B	1.367849	0.000539	Up
TSHZ1	1.396499	0.000106	Up
FGF10	1.434899	5.00E−04	Up
ARRDC3	1.438034	0.000194	Up
FIGN	1.458516	0.000253	Up
NR2F2	1.464969	0.000549	Up
ZNF521	1.487048	0.000283	Up
GPM6B	1.570568	7.60E−05	Up
HOXB5	1.727273	5.70E−05	Up
FRMD5	1.736738	6.20E−05	Up
SFRP2	1.894966	0.000116	Up

Gene symbol	Fold change	p-value	Gene feature
(E) *Genes up/down-regulated in purified TCs*^*SV40*^ *compared with purified primary lung TCs*			
COL3A1	2.033893	5.20E−05	Up
FST	1.763096	5.70E−05	Up
SLIT3	1.476233	6.20E−05	Up
COL1A1	1.754216	6.60E−05	Up
FRMD5	1.529829	7.10E−05	Up
FBN1	1.662323	7.60E−05	Up
ZFP36L1	1.546307	8.10E−05	Up
TNC	1.410268	8.60E−05	Up
PCDH7	1.75739	9.10E−05	Up
DBC1	1.550832	9.60E-05	Up
MMP2	1.365924	0.000101	Up
NNAT	1.470003	0.000106	Up
STAT3	1.55145	0.000111	Up
KLHDC10	− 1.299299	0.000116	Down
KIAA1199	1.437971	0.000121	Up
BHLHE22	1.541751	0.000126	Up
SPRY2	1.451741	0.00013	Up
LOXL1	1.602346	0.000135	Up
TSHZ1	1.635298	0.00014	Up
COL5A2	1.534133	0.000145	Up
HOXA2	1.907531	0.00015	Up
FGF10	1.384277	0.000155	Up
COL12A1	1.291331	0.00016	Up
ZNF503	1.448549	0.000165	Up
NID1	1.254112	0.00017	Up
NFIX	1.50204	0.000175	Up
ANGPTL7	1.516574	0.00018	Up
COL1A2	1.516627	0.000185	Up
IGFBP7	1.527419	0.000189	Up
MEIS1	1.661917	0.000194	Up
ILDR2	1.354674	0.000199	Up
IER5L	1.407578	0.000204	Up
HMGCS1	1.325093	0.000209	Up
CALM3	1.256977	0.000214	Up
CELF2	1.661174	0.000219	Up
PCDH10	1.601334	0.000224	Up
NDST1	1.200203	0.000229	Up
CPXM1	1.387893	0.000234	Up
PPARGC1A	− 1.43313	0.000239	Down
ZEB2	1.389745	0.000244	Up
SPRR1B	− 1.417133	0.000249	Down
MMP16	1.351496	0.000253	Up
RNF150	1.301929	0.000258	Up
LOX	1.275704	0.000263	Up
CBX6	1.291217	0.000273	Up
ZNF521	1.642922	0.000278	Up
MME	1.32368	0.000283	Up
GDPD2	1.247925	0.000288	Up
FMR1	1.627765	0.000293	Up
PDGFRA	1.21296	0.000298	Up
C3orf58	1.227127	0.000303	Up
SFRP2	2.026877	0.000308	Up
FBLN2	1.287435	0.000313	Up
CYP51A1	1.229665	0.000317	Up
ARF6	1.234812	0.000322	Up
RBPJ	− 1.345805	0.000327	Down
GPM6B	1.420512	0.000332	Up
NFIA	1.616514	0.000337	Up
MBD6	− 1.277385	0.000342	Down
EXT1	1.446781	0.000347	Up
MID2	− 1.366576	0.000352	Down
LRP6	1.5391	0.000357	Up
BICC1	1.448877	0.000362	Up
WT1	1.215831	0.000367	Up
CHD3	1.226874	0.000372	Up
THBS1	1.572993	0.000377	Up
UPRT	− 1.413812	0.000381	Down
NLGN2	1.224543	0.000386	Up
NXN	1.274044	0.000391	Up
MED12	− 1.307768	0.000396	Down
LRP1	1.46219	0.000401	Up
HOXB5	1.333689	0.000406	Up
KLF7	1.264161	0.000411	Up
ACLY	1.361581	0.000416	Up
RDH10	1.355596	0.000421	Up
DPYSL2	1.315251	0.000431	Up
PRRX1	1.553947	0.000436	Up
LPHN2	1.258686	0.00044	Up
GATA6	1.43309	0.000445	Up
CDH11	1.32449	0.00045	Up
PRG4	1.243068	0.000455	Up
PCDH17	− 1.543509	0.00046	Down
NFIB	1.600764	0.000465	Up
VCAN	1.21944	0.00047	Up
FAM98A	1.298444	0.000475	Up
EFEMP1	1.226158	0.00048	Up
FOXP2	1.205803	0.000485	Up
PCDHGC5	1.331799	5.00E−04	Up
THBS2	1.266764	0.000504	Up
HIF1A	1.440204	0.000509	Up
IGFBP4	1.30241	0.000514	Up
MARK1	1.233597	0.000519	Up
KMT2D	− 1.200632	0.000524	Down
SCD	1.32132	0.000534	Up
LPCAT3	1.43367	0.000539	Up
TBPL1	− 1.393924	0.000544	Down
PDE1A	1.230559	0.000554	Up
GRIA3	1.359144	0.000564	Up
EFEMP2	1.222385	0.000568	Up
TCF20	− 1.318507	0.000573	Down
PDE7B	1.556907	0.000578	Up
HTRA1	− 1.270642	0.000583	Down
HOXA5	1.58066	0.000593	Up
CYB5R1	− 1.258022	0.000603	Down
SETBP1	1.318692	0.000608	Up
CACNA1C	1.265093	0.000613	Up
ADAMTS2	1.224627	0.000618	Up
DOCK9	− 1.215588	0.000623	Down
OGT	− 1.564357	0.000632	Down
COLEC12	1.376494	0.000637	Up
ZBTB18	− 1.778186	0.000642	Down
KITLG	1.25933	0.000647	Up
GFPT2	1.276106	0.000652	Up
GABRA3	1.435938	0.000657	Up
SLC16A2	1.320833	0.000662	Up
PCDHB1	− 1.242627	0.000667	Down
EPHA7	1.315288	0.000677	Up
SLCO3A1	1.220403	0.000682	Up
DDX26B	− 1.214763	0.000687	Down
SEMA3D	1.235461	0.000692	Up
ADAMTS15	1.389205	0.000696	Up
MAN1A1	1.226513	0.000701	Up
TCF7L2	1.319781	0.000706	Up
PBX3	1.27527	0.000711	Up
SLIT2	1.356085	0.000721	Up
TENM4	− 1.247945	0.000731	Down
PARP6	− 1.252561	0.000736	Down
KLF9	1.320073	0.000755	Up
IGF1	1.441883	0.00076	Up
HOXB8	1.273005	0.00077	Up
ARID5B	1.28496	0.000775	Up
PKM	1.322278	0.00078	Up
BMP1	1.215932	0.000785	Up
PCDH19	− 1.416186	0.000795	Down
FEZ1	− 1.257686	8.00E−04	Down
NPR2	− 1.26446	0.00081	Down
STRBP	− 1.352471	0.000819	Down
DNAJC1	1.241378	0.000824	Up
WNK1	− 1.574397	0.000829	Down
DHCR24	1.236232	0.000844	Up
EGR1	− 1.325378	0.000849	Down
MT1M	1.25661	0.000864	Up
AKAP8L	− 1.20015	0.000874	Down
ATP1B1	1.211897	0.000879	Up
GATM	1.291347	0.000883	Up
TRBV6-8	− 1.35926	0.000893	Down
FZD8	1.649274	0.000903	Up
AHCYL2	− 1.206907	0.000913	Down
PPP3CA	1.344731	0.000918	Up
SURF4	1.211797	0.000923	Up
CCND2	− 1.224408	0.000938	Down
RAB11B	1.26997	0.000943	Up
FHL1	1.202305	0.000952	Up
TOX	1.397482	0.000957	Up
FOXC1	1.242409	0.000967	Up
AIDA	1.274415	0.000977	Up
TNFAIP1	1.202941	0.000982	Up
THBS3	1.214669	0.000987	Up
CLMP	1.294055	0.000992	Up
DOCK3	− 1.231871	0.001002	Down
DDIT3	− 1.40635	0.001006	Down
GPC6	1.248637	0.001011	Up
ABCB7	− 1.307951	0.001016	Down
ETV6	1.353185	0.001026	Up
CHKA	− 1.240302	0.001051	Down
R3HDM2	− 1.252354	0.001061	Down
TLN1	1.226184	0.001085	Up
PFN1	1.394161	0.00109	Up
ADAMTS4	1.218343	0.001095	Up
CLIP3	1.213372	0.0011	Up
ILK	1.304537	0.00111	Up
PLXDC2	1.301745	0.00113	Up
PABPC4	− 1.218394	0.001134	Down
NR2F2	1.485732	0.001174	Up
PPAP2B	1.278773	0.001189	Up
FIGN	1.399002	0.001198	Up
SPARC	1.228554	0.001223	Up
AKT3	− 1.215306	0.001233	Down
TC10000913.hg.1	− 1.44934	0.001238	Down
RHOT1	− 1.311667	0.001243	Down
NUAK1	1.245725	0.001253	Up
SOCS3	1.418689	0.001287	Up
TC02002617.hg.1	− 1.617442	0.001292	Down
TC07000141.hg.1	− 1.394869	0.001302	Down
MYO1D	1.296444	0.001307	Up
CDH2	1.216156	0.001317	Up
TCF4	1.260214	0.001321	Up
FUBP1	− 1.41599	0.001326	Down
ARRDC3	1.309616	0.001341	Up
CYGB	1.229195	0.001351	Up
TBX4	1.238548	0.001356	Up
MUT	− 1.208493	0.001361	Down
MT1H	1.423387	0.001371	Up
MBTPS2	− 1.36196	0.001381	Down
RAB1B	1.221867	0.00143	Up
AZIN1	− 1.289217	0.001435	Down
CNTFR	1.224893	0.001445	Up
NRN1	1.247028	0.001449	Up
PRPF19	1.221176	0.001469	Up
HPRT1	− 1.432868	0.001484	Down
PARVA	1.248515	0.001494	Up
MSN	− 1.307225	0.001499	Down
SPRR2F	− 1.204892	0.001509	Down
DOCK11	− 1.249159	0.001513	Down
H3F3A	1.288991	0.001518	Up
MAP4K3	1.215421	0.001528	Up
RGL1	1.23898	0.001543	Up
PSAT1	− 1.279406	0.001572	Down
BNC1	1.24342	0.001617	Up
HNRNPUL2	1.206099	0.001622	Up
MSI2	− 1.235799	0.001627	Down
FAM71B	1.244138	0.001656	Up
SEC23A	1.252503	0.001661	Up
CNOT6L	− 1.344862	0.001666	Down
CTDSP2	1.310633	0.001676	Up
FGF7	1.282848	0.001691	Up
MYH10	1.216826	0.001696	Up
EMP3	1.28252	0.00171	Up
SOX4	1.304636	0.00172	Up
NR4A3	1.23027	0.00175	Up
USP17	1.224951	0.00176	Up
LDB1	1.225476	0.001764	Up
ATF6	− 1.284147	0.001789	Down
ABI1	1.224035	0.001804	Up
P4HA3	1.256545	0.001814	Up
PTEN	1.427111	0.001838	Up
CCT2	− 1.25044	0.001843	Down
KMT2A	− 1.254142	0.001848	Down
ISM1	1.407921	0.001873	Up
DEFB113	− 1.306901	0.001878	Down
EFNA5	1.216599	0.001942	Up
RBP1	1.223532	0.002006	Up
HSPA9	− 1.22658	0.002015	Down
TRBV21OR9-2	− 1.275752	0.002035	Down
JHDM1D	− 1.22599	0.002089	Down
PRKAA2	− 1.288077	0.002109	Down
GPC3	1.209065	0.002129	Up
INTS5	1.218441	0.002158	Up
EPT1	− 1.215737	0.002163	Down
PAK3	1.209358	0.002178	Up
SARS	− 1.377121	0.002183	Down
MYL9	1.215861	0.002202	Up
CALR	1.328144	0.002217	Up
PLK1	1.234092	0.002222	Up
PDIA3	1.320796	0.002247	Up
C14orf1	1.234254	0.002266	Up
PRELID1	1.300632	0.002281	Up
TC01001567.hg.1	− 1.230659	0.002301	Down
SULF1	1.211791	0.002326	Up
CPSF6	− 1.256973	0.00234	Down
NR4A2	1.213443	0.002345	Up
NR1D1	− 1.219622	0.00235	Down
RAB5C	1.222135	0.002375	Up
REXO2	1.290263	0.002399	Up
LMO4	1.200112	0.002409	Up
GPR174	1.216642	0.002414	Up
AP2S1	1.217261	0.002419	Up
DYNLL2	1.251735	0.002449	Up
HUWE1	− 1.260641	0.002468	Down
VPRBP	− 1.26699	0.002473	Down
BMPR1A	1.33189	0.002478	Up
DIO3	1.203031	0.002493	Up
TM9SF4	1.216029	0.002547	Up
RAB1A	1.410474	0.002572	Up
CEBPB	1.214692	0.002577	Up
ACTR1A	1.216125	0.002606	Up
LOC100509638	− 1.209083	0.002611	Down
CHMP4B	1.224858	0.002636	Up
ARHGAP1	1.325099	0.00265	Up
HMGA1	− 1.240781	0.002655	Down
TOP1	1.272427	0.00267	Up
EGR3	− 1.295132	0.002675	Down
C9orf172	1.228614	0.002685	Up
ID3	1.245858	0.002695	Up
CD248	1.388851	0.0027	Up
FAM168B	1.234437	0.002768	Up
RCN3	1.226194	0.002783	Up
MT1A	1.288399	0.002798	Up
EIF4B	− 1.207714	0.002803	Down
GTPBP2	− 1.208847	0.002813	Down
TMSB10	1.386714	0.002847	Up
GABARAP	1.284343	0.002852	Up
IGHV3-38	− 1.236566	0.002916	Down
TC11000974.hg.1	− 1.267039	0.002931	Down
RAB39B	− 1.258083	0.002985	Down
MMP14	1.257854	0.003034	Up
NFAT5	− 1.270263	0.003069	Down
LCE3D	− 1.208922	0.003093	Down
TMTC2	1.248958	0.003108	Up
USP12	1.408625	0.003133	Up
ANKRD1	− 1.219365	0.003152	Down
RBBP5	− 1.210453	0.003172	Down
PTMA	1.251584	0.003197	Up
ACTA1	1.225395	0.003246	Up
RNF141	− 1.205359	0.00333	Down
FGFBP1	− 1.200483	0.003364	Down
RPL4	− 1.217294	0.003408	Down
CASK	− 1.354502	0.003413	Down
LCE3E	− 1.213158	0.003453	Down
EPC2	1.261066	0.003467	Up
NOXRED1	− 1.234805	0.003507	Down
MT1B	1.263775	0.003517	Up
CCR7	− 1.260932	0.003561	Down
BRAF	− 1.278428	0.003595	Down
RPS27	− 1.246149	0.0036	Down
HNRNPH2	1.328316	0.00361	Up
IGHV2-26	− 1.203819	0.00364	Down
DEFB115	− 1.226413	0.003679	Down
PTBP2	− 1.331489	0.003763	Down
RPS6KB1	1.216347	0.003896	Up
MT2A	1.290212	0.003935	Up
TARDBP	− 1.205193	0.003969	Down
SYT11	1.24811	0.004014	Up
MT1F	1.263703	0.004024	Up
SFRP1	1.245684	0.004053	Up
SEMA3A	− 1.202169	0.004073	Down
TC14000204.hg.1	1.20508	0.004122	Up
TSC22D2	− 1.302317	0.004127	Down
KCNJ2	1.282504	0.004176	Up
SPTLC2	1.242016	0.004309	Up
POLA1	− 1.263183	0.004373	Down
HIPK3	1.207585	0.004398	Up
HIST2H2AA4	1.242261	0.004407	Up
TEAD1	1.201058	0.004432	Up
FBXO33	1.217546	0.004471	Up
ENOX2	− 1.227983	0.00453	Down
OR5A2	− 1.31152	0.00455	Down
THOC2	− 1.305497	0.004555	Down
CAPN6	− 1.245443	0.004614	Down
ZNF281	1.297079	0.004624	Up
NFKBIA	1.21647	0.004649	Up
TBC1D19	− 1.208949	0.004683	Down
TC03000927.hg.1	− 1.21805	0.004688	Down
EDDM3B	1.206252	0.004693	Up
TC19000375.hg.1	− 1.233615	0.004703	Down
OR5AU1	− 1.225907	0.004752	Down
PSMA3	1.210788	0.004781	Up
PABPC1	− 1.24424	0.004796	Down
OR2AJ1	− 1.209642	0.004988	Down
PPIAL4A	1.303974	0.004993	Up
FIGF	1.222296	0.005082	Up
TP53INP1	− 1.227022	0.00519	Down
DNAJA2	1.20171	0.005215	Up
SAP30	1.223176	0.005254	Up
OR4F21	− 1.206284	0.005461	Down
OR1L3	− 1.211827	0.005485	Down
TBX15	1.209874	0.005638	Up
IPO7	− 1.287169	0.005751	Down
ACVR2A	1.227166	0.005889	Up
FOXG1	1.300571	0.005918	Up
AGFG1	1.205554	0.006032	Up
MURC	− 1.204566	0.006169	Down
OR10D3	− 1.304006	0.006337	Down
ADAMTS5	1.212932	0.006342	Up
EID3	1.207392	0.007065	Up
MT1G	1.209678	0.007784	Up
MSANTD2	− 1.200157	0.007966	Down
KDM6A	− 1.246053	0.007971	Down
DERL2	1.224813	0.008443	Up
HSPA5	1.242949	0.008561	Up
POM121L2	− 1.202141	0.009423	Down
CCIN	1.410994	0.009492	Up
TC11000975.hg.1	− 1.23782	0.009501	Down
SYNCRIP	1.202486	0.009526	Up
OR1F1	− 1.200102	0.009708	Down
TRAM1L1	1.227933	0.009723	Up
PPIAL4C	1.292198	0.009954	Up
TC11000959.hg.1	− 1.206482	0.010584	Down
KRTAP29-1	− 1.332407	0.010889	Down
TC07000218.hg.1	− 1.236963	0.011884	Down
ALX1	− 1.216059	0.012036	Down
HEXIM1	1.213559	0.012292	Up
OR10J5	− 1.31215	0.012991	Down
IGLJ4	1.339919	0.014029	Up
C10orf85	1.259314	0.014064	Up
RNF122	1.213118	0.014384	Up
PPIAL4C	1.217453	0.015481	Up

Gene symbol	p-value	q-value	Gene feature
(F) *Genes up/down-regulated in purified primary lung TCs compared with non-purified primary lung TCs*			
ZNF521	5.20E−05	0	Down
NFIX	6.20E−05	0	Down
ZFP36L2	0.000263	0.038653	Down
NR2F2	0.00013	0	Down
SLIT2	7.60E−05	0	Down
NFIB	0.000155	0	Down
NFAT5	0.000288	0.038653	Down
LRP6	5.70E−05	0	Down
FAM102B	0.00019	0	Down
TCF4	0.000372	0.045591	Down
HES1	0.000406	0.045591	Down
PFN1	0.000387	0.045591	Down
TSHZ1	5.00E−04	0.04939	Down
SPRY2	0.000268	0.038653	Down
CTGF	9.10E−05	0	Down
ARRDC3	0.000244	0.038653	Down
LOXL1	0.000682	0.04939	Down
NLGN2	0.000116	0	Down
ZEB2	0.00017	0	Down
CTDSP2	0.00051	0.04939	Down
FBLN5	0.000293	0.038653	Down
FRMD5	0.000342	0.045591	Down
RGL1	0.000416	0.045591	Down

**Table 2:** Summary of LncRNA expressed preferentially in primary TCs, SV40-transformed TCs, primary lung cells, and SV40-transformed primary lung cells

Compared pairs	Up > 0	Up > 2	Up > 4	Down > 0	Down > 2	Down > 4
Purified TCs^SV40^ vs. non-purified primary lung TCs	77	3	0	579	16	2
Non-purified lung TCs^SV40^ vs. purified primary lung TCs	306	15	4	756	15	0
Non-purified lung TCs^SV40^ vs. non-purified primary lung TCs	114	7	2	607	20	0
Purified TCs^SV40^ vs. non-purified lung TCs^SV40^	7	0	0	41	4	1
Purified TCs^SV40^ vs. purified primary lung TCs	112	11	0	499	12	1
Purified primary lung TCs vs. non-purified primary lung TCs	0	0	0	13	2	1

Probe set ID	Fold change	p-value	Gene feature
(A) *Genes up/down-regulated in SV40-transformed TCs compared with non-purified primary lung TCs*			
TC17000728.hg.1	− 3.568496	2.70E−05	Down
TC06001978.hg.1	− 2.084397	3.00E−05	Down
TC08000302.hg.1	− 1.909385	3.30E−05	Down
TC07001784.hg.1	− 1.941664	3.50E−05	Down
TC03003114.hg.1	2.957271	3.80E−05	Up
TC11002382.hg.1	− 5.521638	4.00E−05	Down
TC09000963.hg.1	− 2.006316	4.30E−05	Down
TC15000452.hg.1	− 2.008375	4.60E−05	Down
TC16001289.hg.1	− 1.75485	4.80E−05	Down
TC02000396.hg.1	− 1.817337	5.10E−05	Down
TC06000639.hg.1	− 2.1972	5.40E−05	Down
TC12000927.hg.1	− 2.098063	5.60E−05	Down
TC08002253.hg.1	− 3.102457	5.90E−05	Down
TC11001010.hg.1	− 1.660071	6.10E−05	Down
TC17001801.hg.1	− 2.429971	6.40E−05	Down
TC02001602.hg.1	− 1.575753	6.70E−05	Down
TC02001940.hg.1	− 1.965258	6.90E−05	Down
TC07000780.hg.1	− 2.054482	7.20E−05	Down
TC19000767.hg.1	− 1.795968	7.40E−05	Down
TC09001648.hg.1	− 2.05885	7.70E−05	Down
TC05001593.hg.1	− 1.786234	8.00E−05	Down
TC09000971.hg.1	− 1.864315	8.20E−05	Down
TC04000160.hg.1	− 1.653172	8.50E−05	Down
TC0X001624.hg.1	− 1.966802	8.70E−05	Down
TC10002919.hg.1	− 1.570089	9.00E−05	Down
TC03002042.hg.1	− 1.634325	9.30E−05	Down
TC08001415.hg.1	− 1.709011	9.50E−05	Down
TC02000336.hg.1	− 1.6597	9.80E−05	Down
TC01006068.hg.1	− 1.773582	0.000101	Down
TC11000967.hg.1	− 2.600896	0.000103	Down
TC20001277.hg.1	1.567066	0.000106	Up
TC19000951.hg.1	− 1.643102	0.000108	Down
TC05000152.hg.1	− 1.698079	0.000111	Down
TC12000188.hg.1	− 1.705076	0.000114	Down
TC02000205.hg.1	− 1.840391	0.000116	Down
TC02002456.hg.1	− 1.803182	0.000119	Down
TC06002006.hg.1	− 1.642511	0.000121	Down
TC01003510.hg.1	− 1.59482	0.000124	Down
TC08001864.hg.1	1.822	0.000127	Up
TC20000952.hg.1	− 1.813744	0.000129	Down
TC04002890.hg.1	1.584375	0.000132	Up
TC11002894.hg.1	1.642096	0.000135	Up
TC03001841.hg.1	− 1.694023	0.000137	Down
TC04001635.hg.1	− 2.085812	0.00014	Down
TC04002560.hg.1	2.11556	0.000142	Up
TC16001498.hg.1	1.522853	0.000145	Up
TC15001604.hg.1	− 1.523167	0.000148	Down
TC20000373.hg.1	− 1.641514	0.00015	Down
TC12000633.hg.1	− 1.690876	0.000153	Down
TC13000783.hg.1	− 2.460055	0.000155	Down
TC15001326.hg.1	− 1.512893	0.000158	Down
TC12001286.hg.1	− 1.794425	0.000161	Down
TC01005824.hg.1	− 1.623728	0.000163	Down
TC19001567.hg.1	1.574923	0.000166	Up
TC17001773.hg.1	− 1.52185	0.000168	Down
TC02004814.hg.1	− 1.610212	0.000171	Down
TC19000785.hg.1	− 1.687945	0.000174	Down
TC12000558.hg.1	− 1.462384	0.000176	Down
TC02003744.hg.1	1.609164	0.000179	Up
TC10000962.hg.1	− 1.497291	0.000182	Down
TC12000231.hg.1	− 1.45832	0.000184	Down
TC11003109.hg.1	2.194265	0.000187	Up
TC09001602.hg.1	− 1.532716	0.000189	Down
TC20000650.hg.1	− 1.709435	0.000192	Down
TC0X001292.hg.1	− 2.223119	0.000195	Down
TC17000491.hg.1	− 1.571049	0.000197	Down
TC02001836.hg.1	− 1.460727	2.00E−04	Down
TC02001799.hg.1	− 1.884772	0.000202	Down
TC11002486.hg.1	− 1.617606	0.000205	Down
TC05001562.hg.1	− 1.506318	0.000208	Down
TC19000163.hg.1	− 1.58983	0.00021	Down
TC03001571.hg.1	− 1.551407	0.000213	Down
TC01002801.hg.1	1.387205	0.000215	Up
TC10000791.hg.1	− 1.567348	0.000218	Down
TC05002279.hg.1	− 1.404635	0.000221	Down
TC14000620.hg.1	− 1.492016	0.000223	Down
TC21000698.hg.1	− 1.432959	0.000226	Down
TC0X001976.hg.1	1.511003	0.000229	Up
TC05003313.hg.1	1.520386	0.000231	Up
TC22000098.hg.1	− 1.597171	0.000234	Down
TC08000801.hg.1	− 1.529742	0.000236	Down
TC22000156.hg.1	1.691877	0.000239	Up
TC16002030.hg.1	− 1.399794	0.000242	Down
TC0X001158.hg.1	− 1.925543	0.000244	Down
TC06000041.hg.1	− 1.3817	0.000247	Down
TC10000866.hg.1	− 1.499786	0.000249	Down
TC03000195.hg.1	− 1.569494	0.000252	Down
TC01003308.hg.1	− 1.760166	0.000255	Down
TC01004112.hg.1	− 1.64911	0.000257	Down
TC0X001770.hg.1	− 1.818282	0.00026	Down
TC04000508.hg.1	− 1.514532	0.000262	Down
TC06000705.hg.1	− 1.536016	0.000265	Down
TC02003743.hg.1	1.526381	0.000268	Up
TC05002800.hg.1	− 1.561318	0.00027	Down
TC01005268.hg.1	1.82533	0.000273	Up
TC08000450.hg.1	− 1.626193	0.000276	Down
TC10000439.hg.1	− 1.447509	0.000278	Down
TC06004042.hg.1	− 1.43228	0.000281	Down
TC09002851.hg.1	− 1.367414	0.000283	Down
TC06001651.hg.1	− 1.434151	0.000286	Down
TC07003059.hg.1	1.731542	0.000289	Up
TC10000686.hg.1	− 1.370893	0.000291	Down
TC08000385.hg.1	− 1.537691	0.000294	Down
TC02001432.hg.1	− 1.513117	0.000296	Down
TC01002746.hg.1	− 1.610494	0.000299	Down
TC03002116.hg.1	− 1.353733	0.000302	Down
TC01005428.hg.1	1.560865	0.000304	Up
TC11003210.hg.1	1.443241	0.000307	Up
TC21000149.hg.1	− 1.397728	0.00031	Down
TC09001534.hg.1	− 1.579598	0.000312	Down
TC0X000450.hg.1	− 1.38553	0.000315	Down
TC0X001136.hg.1	− 1.386582	0.000317	Down
TC06002801.hg.1	− 1.504601	0.00032	Down
TC12002688.hg.1	− 1.609811	0.000323	Down
TC04002443.hg.1	− 1.520125	0.000325	Down
TC16001253.hg.1	− 1.436957	0.000328	Down
TC06002402.hg.1	1.593718	0.00033	Up
TC04001524.hg.1	− 1.497563	0.000333	Down
TC22001417.hg.1	− 1.526196	0.000336	Down
TC10001922.hg.1	1.256632	0.000338	Up
TC02003914.hg.1	− 1.445488	0.000341	Down
TC16000510.hg.1	− 1.576476	0.000343	Down
TC01002976.hg.1	− 1.493934	0.000346	Down
TC0M000020.hg.1	− 5.95641	0.000349	Down
TC01002798.hg.1	− 1.77144	0.000351	Down
TC01006074.hg.1	− 1.417269	0.000354	Down
TC06000568.hg.1	− 1.558205	0.000357	Down
TC12000088.hg.1	1.369654	0.000359	Up
TC16001359.hg.1	1.37628	0.000362	Up
TC03001321.hg.1	− 1.300817	0.000364	Down
TC07002531.hg.1	1.474871	0.000367	Up
TC0X000233.hg.1	− 1.387042	0.00037	Down
TC0X000985.hg.1	− 1.911084	0.000372	Down
TC0X001762.hg.1	− 1.495234	0.000375	Down
TC05000067.hg.1	1.605557	0.000377	Up
TC09001461.hg.1	− 1.504051	0.00038	Down
TC15000651.hg.1	− 1.579594	0.000383	Down
TC0X001889.hg.1	1.389217	0.000385	Up
TC15002609.hg.1	1.387993	0.000388	Up
TC14000925.hg.1	− 1.487896	0.00039	Down
TC22001175.hg.1	− 1.488885	0.000393	Down
TC04001945.hg.1	− 1.669152	0.000396	Down
TC09000096.hg.1	− 1.861195	0.000398	Down
TC05002818.hg.1	− 1.472401	0.000401	Down
TC07002463.hg.1	1.484579	0.000404	Up
TC01003990.hg.1	− 1.543233	0.000406	Down
TC06003126.hg.1	− 1.312105	0.000409	Down
TC12000805.hg.1	− 1.554705	0.000411	Down
TC17002196.hg.1	1.345657	0.000414	Up
TC04000811.hg.1	− 1.321197	0.000422	Down
TC22000646.hg.1	1.223546	0.000424	Up
TC11003382.hg.1	1.556575	0.000427	Up
TC15001184.hg.1	− 1.306114	0.00043	Down
TC01000388.hg.1	1.394364	0.000432	Up
TC03003257.hg.1	− 1.314749	0.000435	Down
TC08000285.hg.1	− 1.561996	0.000437	Down
TC0X001410.hg.1	− 1.54477	0.00044	Down
TC07000478.hg.1	− 1.522336	0.000443	Down
TC12002858.hg.1	1.359725	0.000448	Up
TC03001056.hg.1	− 1.423964	0.000451	Down
TC15001493.hg.1	− 1.264085	0.000456	Down
TC05001001.hg.1	− 1.366729	0.000458	Down
TC02001858.hg.1	− 1.336156	0.000461	Down
TC10002369.hg.1	1.416824	0.000464	Up
TC01002620.hg.1	− 1.372948	0.000466	Down
TC12001449.hg.1	− 1.559039	0.000477	Down
TC15002177.hg.1	1.204774	0.000479	Up
TC10001021.hg.1	− 1.351391	0.000482	Down
TC0X002006.hg.1	− 1.276702	0.000487	Down
TC0M000021.hg.1	− 1.76836	0.000492	Down
TC02001302.hg.1	− 1.500158	0.000498	Down
TC07000690.hg.1	− 1.464123	0.000505	Down
TC05000861.hg.1	− 1.43654	0.000508	Down
TC05003112.hg.1	− 1.883715	0.000511	Down
TC22000400.hg.1	− 1.397564	0.000513	Down
TC14001892.hg.1	− 1.409308	0.000516	Down
TC11002784.hg.1	1.463689	0.000518	Up
TC0X001907.hg.1	1.631743	0.000526	Up
TC0X001411.hg.1	− 1.575078	0.000529	Down
TC06000972.hg.1	− 1.412607	0.000532	Down
TC07000702.hg.1	− 1.574012	0.000534	Down
TC15001073.hg.1	− 1.487709	0.000537	Down
TC08002416.hg.1	− 1.30562	0.000539	Down
TC05000611.hg.1	− 1.342755	0.000542	Down
TC09001256.hg.1	− 1.245674	0.000545	Down
TC0M000023.hg.1	− 1.665398	0.000552	Down
TC16001522.hg.1	− 1.550585	0.000555	Down
TC02001429.hg.1	− 1.384528	0.000558	Down
TC05000688.hg.1	− 1.465004	0.00056	Down
TC07001655.hg.1	− 1.483501	0.000563	Down
TC09002148.hg.1	− 1.294238	0.000565	Down
TC03002425.hg.1	− 1.414502	0.000568	Down
TC17002307.hg.1	1.574493	0.000571	Up
TC11002476.hg.1	− 1.407878	0.000573	Down
TC07002321.hg.1	1.340462	0.000576	Up
TC10001073.hg.1	1.420479	0.000579	Up
TC13001025.hg.1	1.281803	0.000581	Up
TC18000050.hg.1	− 1.43278	0.000586	Down
TC08001833.hg.1	1.301019	0.000589	Up
TC02001366.hg.1	− 1.411719	0.000594	Down
TC09002445.hg.1	− 1.365813	0.000597	Down
TC12000244.hg.1	− 1.456782	0.000599	Down
TC16000919.hg.1	− 1.412488	0.000602	Down
TC01001291.hg.1	1.30011	0.000605	Up
TC04002772.hg.1	− 1.318518	0.000607	Down
TC09001898.hg.1	− 1.355328	0.00061	Down
TC02000784.hg.1	1.244517	0.000612	Up
TC0X001210.hg.1	− 1.297482	0.000615	Down
TC03001672.hg.1	− 1.300852	0.00062	Down
TC16001991.hg.1	− 1.312834	0.000623	Down
TC20001376.hg.1	1.259521	0.000626	Up
TC12001995.hg.1	− 1.534637	0.000631	Down
TC20001199.hg.1	− 1.438895	0.000633	Down
TC07001788.hg.1	− 1.605406	0.000639	Down
TC08001734.hg.1	− 1.313819	0.000641	Down
TC07001706.hg.1	− 1.621814	0.000645	Down
TC07001709.hg.1	− 1.621814	0.000645	Down
TC03002939.hg.1	1.465012	0.000649	Up
TC11000808.hg.1	− 1.255062	0.000652	Down
TC02002028.hg.1	− 1.408438	0.000654	Down
TC02002581.hg.1	− 1.345698	0.000657	Down
TC01005437.hg.1	− 1.25784	0.000659	Down
TC18000864.hg.1	1.420831	0.000662	Up
TC03003057.hg.1	1.297577	0.000667	Up
TC11002077.hg.1	− 1.516075	0.00067	Down
TC0X001618.hg.1	− 1.55017	0.000673	Down
TC16002013.hg.1	− 1.464825	0.000675	Down
TC02002957.hg.1	− 1.21553	0.000678	Down
TC14001601.hg.1	− 1.266846	0.00068	Down
TC06003365.hg.1	1.258709	0.000683	Up
TC02001623.hg.1	− 1.387294	0.000691	Down
TC0X001430.hg.1	− 1.517692	0.000693	Down
TC02001248.hg.1	− 1.343015	0.000701	Down
TC07000139.hg.1	− 1.548066	0.000704	Down
TC08001239.hg.1	− 1.411515	0.000709	Down
TC16001996.hg.1	− 1.32531	0.000714	Down
TC07001848.hg.1	− 1.223116	0.000717	Down
TC17001317.hg.1	− 1.258521	0.00072	Down
TC06001365.hg.1	− 1.568292	0.000722	Down
TC09000783.hg.1	− 1.241694	0.000725	Down
TC03003056.hg.1	1.309714	0.000727	Up
TC11001141.hg.1	− 1.474463	0.00073	Down
TC15000819.hg.1	− 1.466781	0.000733	Down
TC17000164.hg.1	− 1.381715	0.000743	Down
TC10002136.hg.1	− 1.222626	0.000748	Down
TC12000238.hg.1	− 1.398297	0.000751	Down
TC19002149.hg.1	1.274434	0.000754	Up
TC10001432.hg.1	− 1.395271	0.000756	Down
TC12002396.hg.1	1.37332	0.000759	Up
TC06002303.hg.1	− 1.481066	0.000767	Down
TC02002233.hg.1	1.281339	0.000769	Up
TC05002034.hg.1	− 1.450196	0.000772	Down
TC03002972.hg.1	− 1.372256	0.000777	Down
TC02001721.hg.1	− 1.367369	0.00078	Down
TC05001948.hg.1	− 1.229624	0.000782	Down
TC06003416.hg.1	− 1.25784	0.000785	Down
TC05000214.hg.1	− 1.345182	0.000787	Down
TC0X002258.hg.1	− 1.40134	0.000795	Down
TC13001479.hg.1	1.612735	0.000798	Up
TC01004166.hg.1	− 1.350561	0.000803	Down
TC15000333.hg.1	1.346913	0.000806	Up
TC18000381.hg.1	− 1.459511	0.000808	Down
TC0X001739.hg.1	− 1.221974	0.000821	Down
TC04000854.hg.1	− 1.342571	0.000824	Down
TC14001245.hg.1	− 1.363644	0.000827	Down
TC16000853.hg.1	− 1.526047	0.000829	Down
TC02004957.hg.1	− 1.256608	0.000832	Down
TC16000648.hg.1	− 1.967375	0.000834	Down
TC01002757.hg.1	− 1.315911	0.000837	Down
TC05000183.hg.1	− 1.466612	0.000842	Down
TC08000652.hg.1	− 1.444517	0.000845	Down
TC12003194.hg.1	− 1.396169	0.00085	Down
TC01002633.hg.1	− 1.459449	0.000853	Down
TC02004273.hg.1	− 1.239977	0.000855	Down
TC0X000473.hg.1	− 1.208647	0.000863	Down
TC01001013.hg.1	1.402441	0.000866	Up
TC07003162.hg.1	− 1.286344	0.000871	Down
TC20000340.hg.1	− 1.467848	0.000874	Down
TC02000955.hg.1	− 1.516537	0.000876	Down
TC17001429.hg.1	− 1.288095	0.000884	Down
TC02002970.hg.1	− 1.262033	0.000887	Down
TC19000423.hg.1	− 1.270008	0.000892	Down
TC04002642.hg.1	− 1.379802	0.000895	Down
TC14000739.hg.1	1.321768	0.000902	Up
TC09001812.hg.1	− 1.231765	0.000905	Down
TC15001577.hg.1	− 1.461504	0.000908	Down
TC12003053.hg.1	1.319866	0.00091	Up
TC12002444.hg.1	1.329822	0.000913	Up
TC07001462.hg.1	1.464142	0.000915	Up
TC12001455.hg.1	− 1.443029	0.000918	Down
TC04000571.hg.1	− 1.489108	0.000921	Down
TC0X001193.hg.1	1.282141	0.000923	Up
TC17002577.hg.1	− 1.222474	0.000929	Down
TC0X000986.hg.1	− 1.537172	0.000934	Down
TC18000372.hg.1	1.306631	0.000936	Up
TC12003159.hg.1	− 1.26419	0.000944	Down
TC12001777.hg.1	− 1.387482	0.000947	Down
TC17000629.hg.1	1.506256	0.000952	Up
TC01002397.hg.1	− 1.298179	0.000955	Down
TC11000089.hg.1	− 1.293079	0.00096	Down
TC19001173.hg.1	− 1.788733	0.000962	Down
TC07002196.hg.1	− 1.237867	0.000965	Down
TC01004898.hg.1	1.21174	0.000968	Up
TC15002247.hg.1	1.224493	0.000976	Up
TC05001142.hg.1	− 1.326114	0.000978	Down
TC05000621.hg.1	− 1.464508	0.000989	Down
TC09000642.hg.1	− 1.540151	0.000991	Down
TC09001936.hg.1	− 1.364479	0.000994	Down
TC17002761.hg.1	− 1.269107	0.000999	Down
TC04002187.hg.1	1.408721	0.001002	Up
TC02003570.hg.1	− 1.280663	0.001004	Down
TC0X001616.hg.1	− 1.322208	0.001007	Down
TC09001972.hg.1	− 1.209848	0.001012	Down
TC06003710.hg.1	− 1.497029	0.001015	Down
TC03001944.hg.1	1.214647	0.001017	Up
TC01004109.hg.1	− 1.321069	0.00102	Down
TC17002195.hg.1	1.21302	0.001023	Up
TC05002180.hg.1	− 1.221883	0.001036	Down
TC20001446.hg.1	1.296941	0.001038	Up
TC09002470.hg.1	1.352053	0.001041	Up
TC08001621.hg.1	− 1.246738	0.001043	Down
TC02004746.hg.1	1.298033	0.001046	Up
TC10000082.hg.1	− 1.243931	0.001051	Down
TC08001847.hg.1	1.362437	0.001056	Up
TC10002381.hg.1	− 1.21549	0.001062	Down
TC14001177.hg.1	− 1.270448	0.001064	Down
TC10000872.hg.1	− 1.230721	0.00107	Down
TC06000025.hg.1	1.328452	0.001075	Up
TC01005249.hg.1	− 1.213079	0.001083	Down
TC16001555.hg.1	− 1.208988	0.00109	Down
TC0Y000124.hg.1	1.246549	0.001093	Up
TC11001732.hg.1	− 1.318377	0.001096	Down
TC03000629.hg.1	− 1.439342	0.001098	Down
TC09001843.hg.1	− 1.204212	0.001104	Down
TC09002783.hg.1	− 1.269006	0.001106	Down
TC01004933.hg.1	1.342382	0.001109	Up
TC10000236.hg.1	− 1.319501	0.001114	Down
TC05001371.hg.1	− 1.314131	0.001122	Down
TC14000052.hg.1	− 1.409573	0.001124	Down
TC21000931.hg.1	− 1.264853	0.00113	Down
TC03002997.hg.1	1.270405	0.001132	Up
TC05003198.hg.1	− 1.253982	0.001135	Down
TC02002711.hg.1	− 1.272432	0.001137	Down
TC02004784.hg.1	− 1.200546	0.001143	Down
TC05002575.hg.1	1.219313	0.001145	Up
TC01002594.hg.1	− 1.421401	0.001148	Down
TC16001826.hg.1	− 1.353943	0.001169	Down
TC08001640.hg.1	− 1.376088	0.001171	Down
TC17002837.hg.1	− 1.262026	0.001174	Down
TC08001247.hg.1	− 1.233191	0.001177	Down
TC07000624.hg.1	− 1.263829	0.001179	Down
TC12001263.hg.1	− 1.216333	0.001184	Down
TC06000824.hg.1	− 1.41643	0.00119	Down
TC12000184.hg.1	− 1.273853	0.001192	Down
TC12003089.hg.1	− 1.237444	0.001208	Down
TC10000369.hg.1	− 1.232303	0.001218	Down
TC03000823.hg.1	− 1.318296	0.001231	Down
TC0X000808.hg.1	− 1.294835	0.001237	Down
TC15001473.hg.1	− 1.390182	0.001239	Down
TC01006043.hg.1	− 1.279341	0.001242	Down
TC16001156.hg.1	− 1.347061	0.001245	Down
TC05000708.hg.1	− 1.457383	0.001247	Down
TC07002757.hg.1	− 1.342035	0.00125	Down
TC01003748.hg.1	− 1.247279	0.001252	Down
TC20000045.hg.1	− 1.611347	0.001255	Down
TC19000349.hg.1	− 1.294863	0.001258	Down
TC08002335.hg.1	− 1.575981	0.001273	Down
TC02003970.hg.1	− 1.261765	0.001276	Down
TC07002353.hg.1	− 1.21386	0.001292	Down
TC03000155.hg.1	− 1.308938	0.001294	Down
TC0X002344.hg.1	− 1.336301	0.001299	Down
TC01004110.hg.1	− 1.549747	0.00131	Down
TC12000390.hg.1	− 1.251394	0.001318	Down
TC14000427.hg.1	− 1.249078	0.001323	Down
TC03000342.hg.1	− 1.293203	0.001326	Down
TC09001279.hg.1	− 1.291408	0.001331	Down
TC14000693.hg.1	− 1.497742	0.001344	Down
TC12003094.hg.1	− 1.417707	0.001349	Down
TC06002606.hg.1	− 1.537348	0.001352	Down
TC06001540.hg.1	− 1.242118	0.001354	Down
TC03001808.hg.1	− 1.327132	0.001359	Down
TC10000793.hg.1	− 1.384112	0.001362	Down
TC20001166.hg.1	− 1.216393	0.001383	Down
TC01005264.hg.1	− 1.316901	0.001388	Down
TC09000471.hg.1	− 1.31262	0.001404	Down
TC19002342.hg.1	− 1.360527	0.00142	Down
TC0X001450.hg.1	− 1.226402	0.001433	Down
TC0X001094.hg.1	− 1.281421	0.001448	Down
TC0X000282.hg.1	− 1.397268	0.001451	Down
TC16001699.hg.1	− 1.21053	0.001459	Down
TC08000411.hg.1	− 1.264325	0.001477	Down
TC01003906.hg.1	− 1.277746	0.001485	Down
TC02001952.hg.1	− 1.202124	0.001487	Down
TC03002704.hg.1	− 1.2066	0.00149	Down
TC06002227.hg.1	− 1.536921	0.001493	Down
TC16000560.hg.1	− 1.319732	0.001501	Down
TC0X001939.hg.1	− 1.239263	0.001503	Down
TC11002900.hg.1	− 1.422263	0.001506	Down
TC09000516.hg.1	− 1.3743	0.001521	Down
TC20000564.hg.1	− 1.259432	0.001529	Down
TC08000425.hg.1	− 1.222783	0.001532	Down
TC07000862.hg.1	− 1.318867	0.001534	Down
TC12001821.hg.1	− 1.267134	0.001545	Down
TC11000653.hg.1	− 1.256931	0.001548	Down
TC02004514.hg.1	− 1.278303	0.00155	Down
TC05002588.hg.1	− 1.361963	0.001553	Down
TC06002096.hg.1	− 1.234314	0.001561	Down
TC10001912.hg.1	− 1.30431	0.001563	Down
TC02000188.hg.1	− 1.343934	0.001566	Down
TC03000381.hg.1	− 1.236027	0.0016	Down
TC20000763.hg.1	− 1.225856	0.001602	Down
TC19000786.hg.1	− 1.255489	0.001608	Down
TC08000224.hg.1	− 1.273889	0.00161	Down
TC12001052.hg.1	− 1.452174	0.001613	Down
TC07000779.hg.1	− 1.324442	0.001644	Down
TC12002368.hg.1	− 1.228785	0.001647	Down
TC02001190.hg.1	− 1.338948	0.001652	Down
TC05000526.hg.1	− 1.221816	0.001665	Down
TC04002294.hg.1	− 1.301327	0.001668	Down
TC21000706.hg.1	− 1.298316	0.001686	Down
TC02002768.hg.1	− 1.312801	0.001694	Down
TC17001656.hg.1	− 1.267211	0.001702	Down
TC03001494.hg.1	− 1.366098	0.00172	Down
TC13000481.hg.1	− 1.28573	0.001723	Down
TC16000776.hg.1	− 1.273221	0.001725	Down
TC10002723.hg.1	− 1.376529	0.001728	Down
TC10002728.hg.1	− 1.244225	0.001733	Down
TC01006051.hg.1	− 1.250802	0.001736	Down
TC05001849.hg.1	− 1.217863	0.001743	Down
TC10000232.hg.1	− 1.310615	0.001751	Down
TC01003920.hg.1	− 1.223489	0.001779	Down
TC01003921.hg.1	− 1.223489	0.001779	Down
TC01003922.hg.1	− 1.223489	0.001779	Down
TC01003923.hg.1	− 1.223489	0.001779	Down
TC01003924.hg.1	− 1.223489	0.001779	Down
TC01003925.hg.1	− 1.223489	0.001779	Down
TC01003926.hg.1	− 1.223489	0.001779	Down
TC01003927.hg.1	− 1.223489	0.001779	Down
TC01003929.hg.1	− 1.223489	0.001779	Down
TC01003930.hg.1	− 1.223489	0.001779	Down
TC01003931.hg.1	− 1.223489	0.001779	Down
TC01003932.hg.1	− 1.223489	0.001779	Down
TC01003933.hg.1	− 1.223489	0.001779	Down
TC01003934.hg.1	− 1.223489	0.001779	Down
TC01003935.hg.1	− 1.223489	0.001779	Down
TC01003936.hg.1	− 1.223489	0.001779	Down
TC03003030.hg.1	− 1.478844	0.001803	Down
TC14000845.hg.1	− 1.312238	0.001806	Down
TC05002975.hg.1	− 1.285007	0.001809	Down
TC20000261.hg.1	− 1.329768	0.001817	Down
TC14001281.hg.1	− 1.272863	0.001819	Down
TC05000998.hg.1	− 1.275857	0.00183	Down
TC13001120.hg.1	− 1.345262	0.001832	Down
TC03000057.hg.1	− 1.226194	0.001853	Down
TC11000768.hg.1	− 1.347505	0.001856	Down
TC04001473.hg.1	− 1.385031	0.001861	Down
TC08001306.hg.1	− 1.237854	0.001864	Down
TC02004490.hg.1	− 1.228885	0.001866	Down
TC02004794.hg.1	− 1.244544	0.001877	Down
TC08001540.hg.1	− 1.312764	0.001884	Down
TC03000875.hg.1	− 1.363667	0.001898	Down
TC06001064.hg.1	− 1.411292	0.001903	Down
TC08000407.hg.1	− 1.291451	0.001905	Down
TC10002820.hg.1	− 1.233983	0.001908	Down
TC14000661.hg.1	− 1.226019	0.001937	Down
TC19000244.hg.1	− 1.222774	0.001939	Down
TC09000897.hg.1	− 1.241196	0.001952	Down
TC11002142.hg.1	− 1.530933	0.001978	Down
TC04000338.hg.1	− 1.28848	0.001984	Down
TC12002736.hg.1	− 1.306143	0.001989	Down
TC09000458.hg.1	− 1.260067	0.002005	Down
TC19002478.hg.1	− 1.251909	0.002012	Down
TC17000607.hg.1	− 1.376805	0.00202	Down
TC08002174.hg.1	− 1.252071	0.002049	Down
TC15002522.hg.1	− 1.352134	0.002059	Down
TC07001721.hg.1	− 1.27015	0.002099	Down
TC11000172.hg.1	− 1.297266	0.002101	Down
TC0M000022.hg.1	− 1.790883	0.002127	Down
TC01000642.hg.1	− 1.273087	0.002135	Down
TC04002779.hg.1	− 1.295919	0.002172	Down
TC17000230.hg.1	− 1.28801	0.002177	Down
TC10002673.hg.1	− 1.341757	0.002185	Down
TC02000761.hg.1	− 1.200639	0.00219	Down
TC07001870.hg.1	− 1.503979	0.002198	Down
TC13001485.hg.1	− 1.322513	0.0022	Down
TC0X002104.hg.1	− 1.235461	0.002203	Down
TC01003794.hg.1	− 1.275671	0.002216	Down
TC14000809.hg.1	− 1.232224	0.002247	Down
TC08001563.hg.1	− 1.281014	0.002292	Down
TC02003041.hg.1	− 1.439696	0.002305	Down
TC01000878.hg.1	− 1.200347	0.002313	Down
TC16001204.hg.1	− 1.216715	0.002315	Down
TC10001355.hg.1	− 1.266825	0.002318	Down
TC10001940.hg.1	− 1.302501	0.002334	Down
TC04000456.hg.1	− 1.259762	0.002347	Down
TC19001288.hg.1	− 1.211289	0.002355	Down
TC21000403.hg.1	− 1.262158	0.002365	Down
TC15001614.hg.1	− 1.242348	0.00237	Down
TC01002339.hg.1	− 1.287548	0.002373	Down
TC21000126.hg.1	− 1.220964	0.002375	Down
TC04001229.hg.1	− 1.226388	0.002399	Down
TC10002579.hg.1	− 1.204095	0.002422	Down
TC01003671.hg.1	− 1.209995	0.00243	Down
TC01001982.hg.1	− 1.217144	0.002436	Down
TC15001480.hg.1	− 1.259755	0.002438	Down
TC22001201.hg.1	− 1.534217	0.002443	Down
TC10000404.hg.1	− 1.319697	0.002472	Down
TC01000565.hg.1	− 1.208333	0.002493	Down
TC11001288.hg.1	− 1.381912	0.002496	Down
TC08001096.hg.1	− 1.310737	0.002532	Down
TC17001148.hg.1	− 1.273032	0.002535	Down
TC22001301.hg.1	− 1.306173	0.002566	Down
TC04002309.hg.1	− 1.209813	0.002595	Down
TC21000144.hg.1	− 1.230499	0.0026	Down
TC20000800.hg.1	− 1.228074	0.002613	Down
TC10001508.hg.1	− 1.242578	0.002629	Down
TC11001429.hg.1	− 1.21474	0.002699	Down
TC10001326.hg.1	− 1.260137	0.002712	Down
TC16001788.hg.1	− 1.266336	0.00272	Down
TC01000718.hg.1	− 1.246934	0.002731	Down
TC02004391.hg.1	− 1.248587	0.002752	Down
TC02004870.hg.1	− 1.393997	0.002775	Down
TC02004787.hg.1	− 1.417426	0.002791	Down
TC12000361.hg.1	− 1.251152	0.002793	Down
TC15000227.hg.1	− 1.256604	0.002806	Down
TC01001745.hg.1	− 1.285385	0.002814	Down
TC05002924.hg.1	− 1.252245	0.002817	Down
TC07002052.hg.1	− 1.237299	0.00283	Down
TC03002295.hg.1	− 1.273649	0.002848	Down
TC06001529.hg.1	− 1.485667	0.002863	Down
TC6_apd_hap1000080.hg.1	− 1.485667	0.002863	Down
TC6_cox_hap2000158.hg.1	− 1.485667	0.002863	Down
TC6_dbb_hap3000147.hg.1	− 1.485667	0.002863	Down
TC6_mann_hap4000135.hg.1	− 1.485667	0.002863	Down
TC6_mcf_hap5000135.hg.1	− 1.485667	0.002863	Down
TC6_qbl_hap6000150.hg.1	− 1.485667	0.002863	Down
TC6_ssto_hap7000130.hg.1	− 1.485667	0.002863	Down
TC07002428.hg.1	− 1.377767	0.002882	Down
TC08000954.hg.1	− 1.303647	0.002911	Down
TC06002505.hg.1	− 1.254488	0.002927	Down
TC21000447.hg.1	− 1.250815	0.002953	Down
TC17001238.hg.1	− 1.230333	0.002955	Down
TC15002586.hg.1	− 1.305785	0.002963	Down
TC12000249.hg.1	− 1.225331	0.002976	Down
TC11002394.hg.1	− 1.214327	0.002989	Down
TC01005988.hg.1	− 1.33252	0.003005	Down
TC21000081.hg.1	− 1.227667	0.003015	Down
TC08000521.hg.1	− 1.204106	0.003023	Down
TC09000907.hg.1	− 1.244625	0.003026	Down
TC14000688.hg.1	− 1.223768	0.003062	Down
TC03000666.hg.1	− 1.298135	0.003065	Down
TC09000250.hg.1	− 1.206244	0.003078	Down
TC01004185.hg.1	− 1.401887	0.003086	Down
TC15002417.hg.1	− 1.211724	0.003089	Down
TC06003509.hg.1	− 1.227037	0.003094	Down
TC01004296.hg.1	− 1.218277	0.003143	Down
TC16000068.hg.1	− 1.254429	0.003169	Down
TC01002874.hg.1	− 1.282112	0.003198	Down
TC03000249.hg.1	− 1.287577	0.003209	Down
TC16001224.hg.1	− 1.33179	0.003219	Down
TC21000483.hg.1	− 1.416389	0.003224	Down
TC14001345.hg.1	− 1.283492	0.003237	Down
TC16001268.hg.1	− 1.22617	0.003256	Down
TC02004413.hg.1	− 1.215328	0.003269	Down
TC02001762.hg.1	− 1.263185	0.003271	Down
TC18000587.hg.1	− 1.230616	0.003274	Down
TC10001008.hg.1	− 1.241843	0.003295	Down
TC0Y000340.hg.1	− 1.250366	0.003297	Down
TC13000269.hg.1	− 1.260255	0.0033	Down
TC20000841.hg.1	− 1.232297	0.003318	Down
TC14001908.hg.1	− 1.215891	0.003324	Down
TC19000842.hg.1	− 1.258765	0.003358	Down
TC04000223.hg.1	− 1.241108	0.00336	Down
TC21000697.hg.1	− 1.303591	0.003394	Down
TC19000162.hg.1	− 1.200106	0.003397	Down
TC10000985.hg.1	− 1.215571	0.003402	Down
TC17001640.hg.1	− 1.212141	0.003405	Down
TC05000237.hg.1	− 1.373017	0.003431	Down
TC09000041.hg.1	− 1.214059	0.003441	Down
TC14002178.hg.1	− 1.230783	0.003446	Down
TC05002490.hg.1	− 1.232531	0.003465	Down
TC15002624.hg.1	− 1.272763	0.003483	Down
TC02002012.hg.1	− 1.316727	0.003499	Down
TC09000159.hg.1	− 1.390197	0.003533	Down
TC09001661.hg.1	− 1.227836	0.003577	Down
TC10001055.hg.1	− 1.228079	0.003624	Down
TC08001514.hg.1	− 1.277552	0.003629	Down
TC11000282.hg.1	− 1.329442	0.003642	Down
TC05000068.hg.1	− 1.350724	0.003645	Down
TC11001479.hg.1	− 1.279519	0.003647	Down
TC18000287.hg.1	− 1.200612	0.003655	Down
TC0X000232.hg.1	− 1.202026	0.003658	Down
TC01002467.hg.1	− 1.339589	0.003689	Down
TC01005508.hg.1	− 1.233888	0.003692	Down
TC02000635.hg.1	− 1.204559	0.003708	Down
TC05002063.hg.1	− 1.245733	0.003747	Down
TC0X002282.hg.1	− 1.216687	0.003788	Down
TC06001847.hg.1	− 1.202026	0.003791	Down
TC05003245.hg.1	− 1.245568	0.003825	Down
TC15002600.hg.1	− 1.202092	0.003846	Down
TC05001212.hg.1	− 1.356607	0.003867	Down
TC14001340.hg.1	− 1.246169	0.003903	Down
TC16002021.hg.1	− 1.243627	0.003914	Down
TC19000560.hg.1	− 1.215974	0.003922	Down
TC10000041.hg.1	− 1.253239	0.003961	Down
TC03002003.hg.1	− 1.293355	0.003977	Down
TC01003999.hg.1	− 1.213308	0.004016	Down
TC11003279.hg.1	− 1.239214	0.004073	Down
TC12000588.hg.1	− 1.255784	0.004105	Down
TC03001070.hg.1	− 1.236896	0.004107	Down
TC05000876.hg.1	− 1.344018	0.004112	Down
TC16001209.hg.1	− 1.206352	0.004131	Down
TC22000194.hg.1	− 1.201822	0.004141	Down
TC01000906.hg.1	− 1.237207	0.004146	Down
TC09001410.hg.1	− 1.328568	0.004149	Down
TC15002068.hg.1	− 1.261482	0.004152	Down
TC13000345.hg.1	− 1.211536	0.004193	Down
TC03002998.hg.1	− 1.271102	0.004199	Down
TC03002746.hg.1	− 1.217988	0.004201	Down
TC15000264.hg.1	− 1.253176	0.004219	Down
TC13000676.hg.1	− 1.259466	0.004222	Down
TC13001035.hg.1	− 1.299097	0.00423	Down
TC01000516.hg.1	− 1.415817	0.004238	Down
TC11002367.hg.1	− 1.203564	0.004256	Down
TC09001709.hg.1	− 1.365244	0.004261	Down
TC10001951.hg.1	− 1.254194	0.004269	Down
TC01005957.hg.1	− 1.276394	0.00429	Down
TC21000155.hg.1	− 1.253291	0.004303	Down
TC08001116.hg.1	− 1.261791	0.004358	Down
TC17002050.hg.1	− 1.21877	0.004384	Down
TC11001973.hg.1	− 1.210002	0.004426	Down
TC10001296.hg.1	− 1.200871	0.004441	Down
TC14000289.hg.1	− 1.311616	0.004481	Down
TC12000607.hg.1	− 1.30518	0.004488	Down
TC05002808.hg.1	− 1.219978	0.004507	Down
TC12001404.hg.1	− 1.256127	0.004509	Down
TC22001312.hg.1	− 1.204404	0.004515	Down
TC13000501.hg.1	− 1.213954	0.004517	Down
TC19001712.hg.1	− 1.212198	0.004546	Down
TC05001815.hg.1	− 1.239784	0.004569	Down
TC0X000283.hg.1	− 1.229291	0.004585	Down
TC08001986.hg.1	− 1.281049	0.004588	Down
TC14000798.hg.1	− 1.441811	0.004598	Down
TC16001481.hg.1	− 1.228168	0.004619	Down
TC0X002079.hg.1	− 1.246788	0.004622	Down
TC17000557.hg.1	− 1.302083	0.004624	Down
TC04002474.hg.1	− 1.274389	0.004635	Down
TC17001193.hg.1	− 1.233444	0.004648	Down
TC01000331.hg.1	− 1.248432	0.004663	Down
TC11002966.hg.1	− 1.259327	0.004695	Down
TC06002589.hg.1	− 1.216984	0.004713	Down
TC06002011.hg.1	− 1.434725	0.004731	Down
TC0X000412.hg.1	− 1.250337	0.004744	Down
TC14001352.hg.1	− 1.286012	0.004755	Down
TC18000425.hg.1	− 1.208537	0.004757	Down
TC16002106.hg.1	− 1.246345	0.004784	Down

Probe set ID	Fold change	p-value	Gene feature
(B) *Genes up/down-regulated in non-purified lung TCs*^*SV40*^ *compared with purified primary lung TCs*			
TC07002463.hg.1	2.512854	2.70E−05	Up
TC0M000020.hg.1	5.689014	3.00E−05	Up
TC03003114.hg.1	4.799932	3.20E−05	Up
TC16000648.hg.1	− 2.174193	3.50E−05	Down
TC17001801.hg.1	− 2.453481	3.80E−05	Down
TC02003744.hg.1	1.994216	4.00E−05	Up
TC11002894.hg.1	4.163733	4.30E−05	Up
TC11003109.hg.1	5.91745	4.50E−05	Up
TC20000952.hg.1	− 2.038322	4.80E−05	Down
TC12001286.hg.1	− 1.75483	5.10E−05	Down
TC09000971.hg.1	− 1.873134	5.30E−05	Down
TC02002456.hg.1	− 1.779116	5.60E−05	Down
TC04002560.hg.1	2.777915	5.80E−05	Up
TC05003313.hg.1	2.480494	6.10E−05	Up
TC02000205.hg.1	− 1.822189	6.40E−05	Down
TC08002510.hg.1	2.45585	6.60E−05	Up
TC20000045.hg.1	− 2.011887	6.90E−05	Down
TC12000927.hg.1	− 2.097234	7.10E−05	Down
TC09001648.hg.1	− 1.962423	7.40E−05	Down
TC0X000985.hg.1	− 2.143818	7.70E−05	Down
TC01003308.hg.1	− 1.928676	7.90E−05	Down
TC22000156.hg.1	1.958661	8.20E−05	Up
TC04001945.hg.1	− 2.062837	8.40E−05	Down
TC06000639.hg.1	− 2.085546	8.70E−05	Down
TC06001978.hg.1	− 1.915437	9.00E−05	Down
TC15000452.hg.1	− 1.936021	9.20E−05	Down
TC02001940.hg.1	− 1.951298	9.50E−05	Down
TC06000568.hg.1	− 1.714082	9.70E−05	Down
TC08000302.hg.1	− 1.960068	1.00E−04	Down
TC17002479.hg.1	1.736394	0.000102	Up
TC04002890.hg.1	2.211564	0.000105	Up
TC20000650.hg.1	− 1.762113	0.000108	Down
TC02004814.hg.1	− 1.74863	0.00011	Down
TC17001773.hg.1	− 1.57464	0.000113	Down
TC07001784.hg.1	− 1.815099	0.000115	Down
TC05001593.hg.1	− 1.890235	0.000118	Down
TC0X001889.hg.1	1.807993	0.000121	Up
TC11002382.hg.1	− 2.886542	0.000123	Down
TC0M000022.hg.1	2.047915	0.000126	Up
TC06004042.hg.1	− 1.606168	0.000128	Down
TC12001449.hg.1	− 1.770781	0.000131	Down
TC09000096.hg.1	− 1.882465	0.000134	Down
TC0X001824.hg.1	1.668798	0.000136	Up
TC08000801.hg.1	− 1.742013	0.000139	Down
TC01005811.hg.1	1.59682	0.000141	Up
TC0X001410.hg.1	− 1.540303	0.000144	Down
TC13000783.hg.1	− 2.094361	0.000147	Down
TC0X001411.hg.1	− 1.589254	0.000149	Down
TC07000702.hg.1	− 1.788577	0.000152	Down
TC07002531.hg.1	1.762967	0.000154	Up
TC20000373.hg.1	− 1.578384	0.000157	Down
TC06002402.hg.1	1.778454	0.00016	Up
TC09001483.hg.1	1.350997	0.000162	Up
TC07001655.hg.1	− 1.528388	0.000165	Down
TC01004012.hg.1	1.503918	0.000167	Up
TC0X001136.hg.1	− 1.534991	0.00017	Down
TC01002339.hg.1	− 1.538502	0.000173	Down
TC12000633.hg.1	− 1.841917	0.000175	Down
TC02001602.hg.1	− 1.669996	0.000178	Down
TC16001991.hg.1	− 1.351113	0.00018	Down
TC03001841.hg.1	− 1.638782	0.000183	Down
TC22001175.hg.1	− 1.558447	0.000186	Down
TC14001892.hg.1	− 1.603015	0.000188	Down
TC08002500.hg.1	− 1.406478	0.000191	Down
TC02004615.hg.1	1.498343	0.000193	Up
TC12000231.hg.1	− 1.476476	0.000196	Down
TC14000711.hg.1	− 1.357936	0.000198	Down
TC16001289.hg.1	− 1.683139	0.000201	Down
TC0X001822.hg.1	1.466789	0.000204	Up
TC02000396.hg.1	− 1.782594	0.000206	Down
TC15002247.hg.1	1.443916	0.000209	Up
TC10000686.hg.1	− 1.508486	0.000211	Down
TC22000400.hg.1	− 1.524343	0.000214	Down
TC06003416.hg.1	− 1.409684	0.000217	Down
TC16002030.hg.1	− 1.497175	0.000219	Down
TC01002976.hg.1	− 1.555849	0.000222	Down
TC15001326.hg.1	− 1.510761	0.000224	Down
TC07002052.hg.1	− 1.585855	0.000227	Down
TC12001995.hg.1	− 1.471839	0.00023	Down
TC19000163.hg.1	− 1.563519	0.000232	Down
TC21000931.hg.1	− 1.527689	0.000235	Down
TC02003743.hg.1	1.519001	0.000237	Up
TC10000866.hg.1	− 1.606186	0.00024	Down
TC04002346.hg.1	1.48682	0.000243	Up
TC08001887.hg.1	1.336453	0.000245	Up
TC06001651.hg.1	− 1.472924	0.000248	Down
TC04000811.hg.1	− 1.415531	0.00025	Down
TC0X000279.hg.1	− 1.52073	0.000253	Down
TC04001635.hg.1	− 2.149662	0.000256	Down
TC19000767.hg.1	− 1.792384	0.000258	Down
TC04000160.hg.1	− 1.443667	0.000261	Down
TC16001498.hg.1	1.716487	0.000263	Up
TC04001524.hg.1	− 1.497583	0.000266	Down
TC01002798.hg.1	− 1.728642	0.000269	Down
TC04002187.hg.1	1.535246	0.000271	Up
TC05002063.hg.1	− 1.378512	0.000274	Down
TC09002847.hg.1	− 1.569396	0.000276	Down
TC05002818.hg.1	− 1.548005	0.000279	Down
TC0X001292.hg.1	− 2.249923	0.000282	Down
TC0X000450.hg.1	− 1.560574	0.000284	Down
TC03001056.hg.1	− 1.589272	0.000287	Down
TC09001898.hg.1	− 1.413369	0.000289	Down
TC02000955.hg.1	− 1.593632	0.000292	Down
TC02001432.hg.1	− 1.603841	0.000294	Down
TC03000195.hg.1	− 1.56613	0.000297	Down
TC01002877.hg.1	1.393011	3.00E−04	Up
TC02001366.hg.1	− 1.50307	0.000302	Down
TC06001365.hg.1	− 1.798942	0.000305	Down
TC21000483.hg.1	− 1.526505	0.000307	Down
TC11002486.hg.1	− 1.571098	0.00031	Down
TC10002723.hg.1	− 1.737066	0.000313	Down
TC09002408.hg.1	2.424384	0.000315	Up
TC02002994.hg.1	− 1.689071	0.000318	Down
TC01000642.hg.1	− 1.53535	0.00032	Down
TC14000620.hg.1	− 1.463791	0.000323	Down
TC07003059.hg.1	1.529988	0.000326	Up
TC21000650.hg.1	− 1.430525	0.000328	Down
TC11001010.hg.1	− 1.579571	0.000331	Down
TC05000526.hg.1	− 1.395094	0.000333	Down
TC09001461.hg.1	− 1.680036	0.000336	Down
TC09002258.hg.1	1.60311	0.000339	Up
TC07001656.hg.1	− 1.511784	0.000341	Down
TC10000439.hg.1	− 1.472641	0.000344	Down
TC05002279.hg.1	− 1.557636	0.000346	Down
TC14000775.hg.1	1.356138	0.000349	Up
TC14002059.hg.1	1.523057	0.000352	Up
TC15001184.hg.1	− 1.391194	0.000357	Down
TC11003147.hg.1	− 1.401112	0.000359	Down
TC07002742.hg.1	− 1.399246	0.000362	Down
TC08001864.hg.1	2.950612	0.000365	Up
TC09001602.hg.1	− 1.880185	0.000367	Down
TC05002800.hg.1	− 1.563391	0.00037	Down
TC11002476.hg.1	− 1.690386	0.000372	Down
TC07002321.hg.1	1.27127	0.000375	Up
TC01002633.hg.1	− 1.686971	0.000378	Down
TC05002022.hg.1	1.497479	0.00038	Up
TC17000728.hg.1	− 1.965686	0.000383	Down
TC10002300.hg.1	1.994904	0.000385	Up
TC01002746.hg.1	− 1.430809	0.000388	Down
TC05000708.hg.1	− 1.617304	0.000391	Down
TC17000491.hg.1	− 1.515315	0.000396	Down
TC09001534.hg.1	− 1.459227	0.000398	Down
TC12003194.hg.1	− 1.49803	0.000401	Down
TC12002688.hg.1	− 1.685657	0.000403	Down
TC04000508.hg.1	− 1.50861	0.000406	Down
TC01002620.hg.1	− 1.500063	0.000409	Down
TC21000887.hg.1	1.383973	0.000414	Up
TC18000603.hg.1	2.095404	0.000416	Up
TC02002711.hg.1	− 1.374202	0.000419	Down
TC01004110.hg.1	1.321687	0.000422	Up
TC06000824.hg.1	− 1.53592	0.000427	Down
TC01004763.hg.1	1.590669	0.000429	Up
TC12000558.hg.1	− 1.427752	0.000432	Down
TC20001202.hg.1	1.308112	0.000435	Up
TC07001267.hg.1	− 1.48023	0.000437	Down
TC06004031.hg.1	− 1.480003	0.00044	Down
TC22001053.hg.1	− 1.545286	0.000442	Down
TC0X001158.hg.1	− 1.890718	0.000445	Down
TC16002013.hg.1	− 1.631206	0.000448	Down
TC04002642.hg.1	− 1.43066	0.000453	Down
TC21000042.hg.1	2.453488	0.000455	Up
TC20001679.hg.1	1.762017	0.000458	Up
TC14001143.hg.1	− 1.26258	0.000461	Down
TC11002810.hg.1	1.384015	0.000463	Up
TC11001848.hg.1	1.462057	0.000466	Up
TC12003094.hg.1	− 1.4237	0.000468	Down
TC11002492.hg.1	− 1.316801	0.000471	Down
TC04000429.hg.1	1.289905	0.000474	Up
TC01005268.hg.1	2.055799	0.000476	Up
TC01000487.hg.1	1.354288	0.000479	Up
TC02001429.hg.1	− 1.455529	0.000487	Down
TC19001967.hg.1	1.548221	0.000489	Up
TC12000813.hg.1	− 1.316062	0.000492	Down
TC22001417.hg.1	− 1.597844	0.000494	Down
TC08000652.hg.1	− 1.574349	0.000497	Down
TC18000408.hg.1	1.572603	0.000499	Up
TC11003186.hg.1	1.33033	0.000502	Up
TC01004251.hg.1	1.32401	0.000505	Up
TC08002253.hg.1	− 2.279339	0.000507	Down
TC01002594.hg.1	− 1.779567	0.00051	Down
TC03003057.hg.1	1.407168	0.000512	Up
TC0X002258.hg.1	− 1.448026	0.000515	Down
TC14001731.hg.1	1.360459	0.000518	Up
TC11000031.hg.1	− 1.412204	0.00052	Down
TC08001833.hg.1	1.37955	0.000525	Up
TC03001342.hg.1	− 1.290438	0.000528	Down
TC17002307.hg.1	1.569429	0.000531	Up
TC04002813.hg.1	1.2442	0.000533	Up
TC05000688.hg.1	− 1.475197	0.000536	Down
TC05002034.hg.1	− 1.389715	0.000538	Down
TC12000088.hg.1	1.466129	0.000544	Up
TC05001142.hg.1	− 1.347908	0.000546	Down
TC06002303.hg.1	− 1.437374	0.000562	Down
TC09000586.hg.1	− 1.463022	0.000564	Down
TC15001796.hg.1	− 1.33117	0.000567	Down
TC17001193.hg.1	− 1.355381	0.00057	Down
TC06002340.hg.1	− 1.4849	0.000575	Down
TC05003112.hg.1	1.638048	0.000577	Up
TC06000705.hg.1	− 1.607894	0.000583	Down
TC12000238.hg.1	− 1.335439	0.000585	Down
TC01000565.hg.1	− 1.337671	0.000588	Down
TC02000092.hg.1	− 1.330873	0.00059	Down
TC0X002282.hg.1	− 1.325366	0.000593	Down
TC09000383.hg.1	− 1.274316	0.000598	Down
TC11003210.hg.1	1.231344	0.000601	Up
TC15001604.hg.1	− 1.487855	0.000603	Down
TC10002919.hg.1	− 1.480823	0.000606	Down
TC21000511.hg.1	1.377046	0.000611	Up
TC02003970.hg.1	− 1.387345	0.000616	Down
TC07002506.hg.1	− 1.369026	0.000619	Down
TC01003622.hg.1	1.356757	0.000621	Up
TC12001052.hg.1	− 1.676926	0.000624	Down
TC13000706.hg.1	− 1.254769	0.000627	Down
TC01004698.hg.1	1.469743	0.000632	Up
TC07001706.hg.1	− 1.439366	0.000636	Down
TC07001709.hg.1	− 1.439366	0.000636	Down
TC05001595.hg.1	− 1.447738	0.00064	Down
TC02001302.hg.1	− 1.576693	0.000642	Down
TC05001591.hg.1	− 1.203418	0.000645	Down
TC08002317.hg.1	− 1.330718	0.000647	Down
TC09000516.hg.1	− 1.664198	0.00065	Down
TC12002858.hg.1	1.350881	0.000655	Up
TC01005360.hg.1	1.271483	0.000658	Up
TC19001567.hg.1	1.505097	0.00066	Up
TC07000690.hg.1	− 1.411787	0.000663	Down
TC03001571.hg.1	− 1.477672	0.000666	Down
TC06002795.hg.1	1.436937	0.000668	Up
TC06003874.hg.1	− 1.29272	0.000671	Down
TC03002116.hg.1	− 1.412674	0.000673	Down
TC05000127.hg.1	1.513078	0.000676	Up
TC17002638.hg.1	1.348987	0.000679	Up
TC20000751.hg.1	− 1.402838	0.000681	Down
TC08002421.hg.1	− 1.74914	0.000684	Down
TC01005354.hg.1	1.229747	0.000686	Up
TC12003088.hg.1	− 1.281487	0.000689	Down
TC08001583.hg.1	− 1.537328	0.000691	Down
TC05000998.hg.1	− 1.483305	0.000694	Down
TC04001473.hg.1	− 1.432026	0.000697	Down
TC04002443.hg.1	− 1.487139	0.000702	Down
TC02000005.hg.1	− 1.472423	0.000704	Down
TC0X001624.hg.1	− 1.596058	0.000707	Down
TC04000461.hg.1	1.412843	0.00071	Up
TC08000450.hg.1	− 1.615791	0.000712	Down
TC18000833.hg.1	1.283471	0.000715	Up
TC01003671.hg.1	− 1.422938	0.00072	Down
TC01001291.hg.1	1.409687	0.000723	Up
TC10001432.hg.1	− 1.432311	0.000725	Down
TC17001310.hg.1	− 1.283493	0.000728	Down
TC08002093.hg.1	− 1.29301	0.00073	Down
TC20001234.hg.1	1.431939	0.000733	Up
TC11000270.hg.1	1.380022	0.000736	Up
TC06002451.hg.1	− 1.361116	0.000738	Down
TC02003138.hg.1	1.349475	0.000741	Up
TC05000183.hg.1	− 1.48138	0.000746	Down
TC01003990.hg.1	− 1.390426	0.000749	Down
TC02001762.hg.1	− 1.302759	0.000756	Down
TC0X001430.hg.1	− 1.470658	0.000762	Down
TC05001244.hg.1	1.28468	0.000764	Up
TC16001854.hg.1	− 1.274852	0.000767	Down
TC19000179.hg.1	− 1.220968	0.000769	Down
TC01005416.hg.1	1.308645	0.000772	Up
TC21000041.hg.1	1.720569	0.000777	Up
TC06002615.hg.1	1.698567	0.000782	Up
TC0X001210.hg.1	− 1.72284	0.000787	Down
TC08000385.hg.1	− 1.30756	0.00079	Down
TC01001501.hg.1	− 1.261062	0.000793	Down
TC11000882.hg.1	1.360165	0.000795	Up
TC17002123.hg.1	1.252373	0.000798	Up
TC10001266.hg.1	− 1.270974	8.00E−04	Down
TC04000223.hg.1	− 1.399388	0.000803	Down
TC02004784.hg.1	− 1.270101	0.000806	Down
TC03003257.hg.1	− 1.473539	0.000811	Down
TC11002784.hg.1	1.544173	0.000816	Up
TC17000637.hg.1	1.549618	0.000821	Up
TC21000447.hg.1	− 1.357028	0.000824	Down
TC16001066.hg.1	− 1.250451	0.000826	Down
TC10001922.hg.1	1.308392	0.000834	Up
TC11002991.hg.1	− 1.203628	0.000839	Down
TC03003014.hg.1	− 1.272182	0.000845	Down
TC05001291.hg.1	− 1.22311	0.000847	Down
TC11002432.hg.1	− 1.294734	0.00085	Down
TC07000282.hg.1	− 1.386049	0.000852	Down
TC0X001907.hg.1	1.514463	0.000855	Up
TC12000589.hg.1	− 1.437338	0.000858	Down
TC01001111.hg.1	1.562931	0.00086	Up
TC08000734.hg.1	− 1.2404	0.000863	Down
TC0X000649.hg.1	− 1.204547	0.000865	Down
TC12000005.hg.1	− 1.230721	0.000868	Down
TC06003061.hg.1	1.681888	0.000871	Up
TC11000655.hg.1	− 1.343701	0.000873	Down
TC09000963.hg.1	− 1.43499	0.000881	Down
TC15002417.hg.1	− 1.286319	0.000883	Down
TC07003181.hg.1	− 1.334931	0.000886	Down
TC10001115.hg.1	1.283726	0.000889	Up
TC12000578.hg.1	1.240881	0.000891	Up
TC01005437.hg.1	1.248673	0.000894	Up
TC17002178.hg.1	− 1.402437	0.000896	Down
TC14001936.hg.1	1.238815	0.000899	Up
TC14000725.hg.1	− 1.30455	0.000904	Down
TC13000186.hg.1	− 1.264527	0.000907	Down
TC05002042.hg.1	− 1.348682	0.000909	Down
TC12001770.hg.1	1.739266	0.000917	Up
TC17001656.hg.1	− 1.467195	0.000922	Down
TC01005905.hg.1	− 1.214045	0.000925	Down
TC0Y000302.hg.1	− 1.280838	0.000931	Down
TC0Y000304.hg.1	− 1.280838	0.000931	Down
TC02004706.hg.1	1.367562	0.000935	Up
TC06003540.hg.1	1.286007	0.000938	Up
TC12001099.hg.1	− 1.215286	0.000941	Down
TC10000843.hg.1	1.313133	0.000943	Up
TC03001294.hg.1	1.437371	0.000946	Up
TC15000406.hg.1	− 1.277322	0.000948	Down
TC05002576.hg.1	− 1.39417	0.000951	Down
TC14000991.hg.1	1.299653	0.000954	Up
TC02003574.hg.1	− 1.248912	0.000956	Down
TC15002494.hg.1	− 1.335358	0.000959	Down
TC10001764.hg.1	− 1.280423	0.000961	Down
TC02001836.hg.1	− 1.445879	0.000964	Down
TC06000102.hg.1	− 1.497939	0.000969	Down
TC03001531.hg.1	1.252613	0.000972	Up
TC08001082.hg.1	− 1.277646	0.000977	Down
TC03000629.hg.1	− 1.58654	0.00098	Down
TC04000854.hg.1	− 1.369976	0.000982	Down
TC0X000283.hg.1	− 1.606025	0.000987	Down
TC02003376.hg.1	1.288982	0.00099	Up
TC16001973.hg.1	1.416974	0.000995	Up
TC09001936.hg.1	− 1.55743	0.000998	Down
TC14002181.hg.1	− 1.292272	0.001	Down
TC19002169.hg.1	1.292553	0.001003	Up
TC22001135.hg.1	− 1.237744	0.001008	Down
TC14001911.hg.1	1.226465	0.001013	Up
TC04001384.hg.1	1.326226	0.001016	Up
TC09002810.hg.1	− 1.253762	0.001018	Down
TC01006061.hg.1	1.239967	0.001024	Up
TC09002176.hg.1	− 1.252655	0.001026	Down
TC0X001762.hg.1	− 1.338176	0.001029	Down
TC07000339.hg.1	− 1.441623	0.001034	Down
TC18000715.hg.1	− 1.339535	0.001039	Down
TC20001391.hg.1	1.279887	0.001042	Up
TC04001218.hg.1	1.463061	0.001044	Up
TC01005893.hg.1	− 1.235444	0.001047	Down
TC03001845.hg.1	1.468478	0.001052	Up
TC01006068.hg.1	− 1.684105	0.001055	Down
TC20000564.hg.1	− 1.33301	0.001057	Down
TC20000106.hg.1	1.445871	0.00106	Up
TC04000543.hg.1	1.606432	0.001063	Up
TC12000805.hg.1	− 1.359918	0.001065	Down
TC04001743.hg.1	1.217508	0.001068	Up
TC09000060.hg.1	− 1.302308	0.00107	Down
TC03001672.hg.1	− 1.340418	0.001073	Down
TC08002335.hg.1	− 1.675618	0.001076	Down
TC09001709.hg.1	− 1.550874	0.001078	Down
TC21000663.hg.1	1.304123	0.001081	Up
TC08002008.hg.1	1.368127	0.001083	Up
TC20000053.hg.1	− 1.230537	0.001088	Down
TC01003510.hg.1	− 1.353839	0.001091	Down
TC11002378.hg.1	1.452002	0.001094	Up
TC03001943.hg.1	− 1.331955	0.001099	Down
TC05002812.hg.1	− 1.25135	0.001101	Down
TC03001366.hg.1	− 1.383993	0.001104	Down
TC01002044.hg.1	1.389355	0.001107	Up
TC03002276.hg.1	− 1.385647	0.001109	Down
TC12000315.hg.1	− 1.378694	0.001112	Down
TC02000413.hg.1	− 1.31176	0.001114	Down
TC08002504.hg.1	− 1.386422	0.001117	Down
TC01001142.hg.1	1.505991	0.001127	Up
TC07001801.hg.1	1.753925	0.00113	Up
TC05002870.hg.1	1.302868	0.001133	Up
TC02002904.hg.1	− 1.387138	0.001135	Down
TC11001108.hg.1	1.530186	0.00114	Up
TC13001025.hg.1	1.287199	0.001146	Up
TC09001939.hg.1	− 1.354181	0.001148	Down
TC19000963.hg.1	1.281426	0.001156	Up
TC14000314.hg.1	1.326006	0.001159	Up
TC06002689.hg.1	1.339061	0.001161	Up
TC11000768.hg.1	− 1.28868	0.001164	Down
TC12000107.hg.1	1.674608	0.001166	Up
TC07002087.hg.1	− 1.291715	0.001172	Down
TC02003914.hg.1	− 1.506351	0.001174	Down
TC11002142.hg.1	− 1.78159	0.001177	Down
TC01002397.hg.1	− 1.278246	0.001179	Down
TC19000409.hg.1	− 1.323654	0.001182	Down
TC02001844.hg.1	1.297798	0.001187	Up
TC07001099.hg.1	− 1.298125	0.00119	Down
TC01004750.hg.1	− 1.393313	0.001192	Down
TC05001001.hg.1	− 1.289514	0.001195	Down
TC14001735.hg.1	1.217484	0.0012	Up
TC02004275.hg.1	− 1.283611	0.001203	Down
TC08001850.hg.1	1.293788	0.001205	Up
TC09000043.hg.1	1.326023	0.001208	Up
TC02000138.hg.1	1.378171	0.00121	Up
TC0X002006.hg.1	− 1.254125	0.001213	Down
TC05001174.hg.1	1.248122	0.001216	Up
TC18000381.hg.1	− 1.439065	0.001218	Down
TC0X002099.hg.1	1.697539	0.001221	Up
TC15001301.hg.1	1.38106	0.001223	Up
TC01000109.hg.1	1.392654	0.001231	Up
TC0X001298.hg.1	1.461986	0.001234	Up
TC0X001901.hg.1	− 1.233679	0.001239	Down
TC04002100.hg.1	− 1.359631	0.001244	Down
TC20000460.hg.1	− 1.318744	0.001247	Down
TC0X000064.hg.1	1.391058	0.001252	Up
TC0X001094.hg.1	− 1.429617	0.001257	Down
TC11002051.hg.1	− 1.484736	0.00126	Down
TC12001929.hg.1	− 1.342651	0.001265	Down
TC11003367.hg.1	1.656682	0.001268	Up
TC02003380.hg.1	1.301934	0.001275	Up
TC21000762.hg.1	1.361985	0.001278	Up
TC0M000023.hg.1	1.382876	0.00128	Up
TC02004390.hg.1	1.446827	0.001283	Up
TC10002633.hg.1	− 1.301706	0.001288	Down
TC01006076.hg.1	− 1.245012	0.001291	Down
TC12002203.hg.1	1.526303	0.001296	Up
TC11002740.hg.1	− 1.283389	0.001299	Down
TC04002160.hg.1	− 1.262713	0.001301	Down
TC17002633.hg.1	1.378591	0.001304	Up
TC12001768.hg.1	− 1.398601	0.001306	Down
TC04002095.hg.1	1.241208	0.001312	Up
TC05001178.hg.1	1.339261	0.001317	Up
TC04001688.hg.1	1.362728	0.001319	Up
TC10002901.hg.1	− 1.278397	0.001322	Down
TC15001808.hg.1	1.434852	0.00133	Up
TC04000710.hg.1	1.355916	0.001332	Up
TC10002092.hg.1	1.281193	0.001335	Up
TC09002305.hg.1	1.26724	0.001343	Up
TC12000651.hg.1	− 1.351627	0.001345	Down
TC10001511.hg.1	− 1.316949	0.001356	Down
TC01000472.hg.1	− 1.298179	0.001358	Down
TC11003246.hg.1	1.335547	0.001366	Up
TC11000161.hg.1	− 1.27089	0.001374	Down
TC12000359.hg.1	1.237241	0.001379	Up
TC12001702.hg.1	− 1.275897	0.001382	Down
TC08001540.hg.1	− 1.346581	0.001384	Down
TC08001886.hg.1	1.259731	0.001387	Up
TC13000269.hg.1	− 1.424301	0.001389	Down
TC01005067.hg.1	1.487055	0.001392	Up
TC01003976.hg.1	1.273839	0.001395	Up
TC10000265.hg.1	1.547355	0.001402	Up
TC04001202.hg.1	− 1.265554	0.001405	Down
TC02001892.hg.1	− 1.479308	0.00141	Down
TC0X001653.hg.1	− 1.20304	0.001413	Down
TC17001939.hg.1	− 1.305775	0.001415	Down
TC14001708.hg.1	1.42332	0.001423	Up
TC20001199.hg.1	− 1.794386	0.001426	Down
TC06004094.hg.1	− 1.213016	0.001434	Down
TC11003053.hg.1	1.483375	0.001436	Up
TC01001470.hg.1	− 1.244999	0.001439	Down
TC04001675.hg.1	1.367312	0.001441	Up
TC05001384.hg.1	− 1.20032	0.001444	Down
TC22000509.hg.1	1.20276	0.001449	Up
TC10000687.hg.1	1.301899	0.001454	Up
TC11000849.hg.1	1.465989	0.001457	Up
TC15002506.hg.1	1.432903	0.00146	Up
TC14001281.hg.1	− 1.515784	0.001462	Down
TC05002143.hg.1	− 1.507259	0.001465	Down
TC07001428.hg.1	− 1.281883	0.001467	Down
TC07002487.hg.1	− 1.220035	0.001472	Down
TC13000434.hg.1	− 1.269599	0.001475	Down
TC17000700.hg.1	1.521397	0.001478	Up
TC06000356.hg.1	1.234269	0.00148	Up
TC07000879.hg.1	1.442272	0.001483	Up
TC16000168.hg.1	− 1.307892	0.001485	Down
TC07002026.hg.1	− 1.230563	0.001491	Down
TC15002522.hg.1	− 1.247948	0.001493	Down
TC03002349.hg.1	1.308143	0.001496	Up
TC04001294.hg.1	− 1.255584	0.001498	Down
TC01005264.hg.1	− 1.438176	0.001501	Down
TC05002959.hg.1	− 1.301601	0.001504	Down
TC08001642.hg.1	1.379421	0.001506	Up
TC12001758.hg.1	− 1.281904	0.001509	Down
TC11002955.hg.1	1.23146	0.001514	Up
TC01006001.hg.1	− 1.231238	0.001519	Down
TC11001275.hg.1	− 1.326731	0.001522	Down
TC09000835.hg.1	− 1.232379	0.001524	Down
TC10001178.hg.1	1.259514	0.001527	Up
TC01004281.hg.1	− 1.281757	0.00153	Down
TC12002368.hg.1	− 1.214252	0.001532	Down
TC03002510.hg.1	− 1.22681	0.001537	Down
TC18000199.hg.1	1.359443	0.00154	Up
TC03000889.hg.1	− 1.378763	0.001548	Down
TC16001163.hg.1	1.522691	0.001553	Up
TC16000573.hg.1	− 1.384428	0.001561	Down
TC22001327.hg.1	− 1.261884	0.001563	Down
TC19000423.hg.1	− 1.349595	0.001566	Down
TC19000016.hg.1	− 1.203688	0.001569	Down
TC12000395.hg.1	1.483422	0.001571	Up
TC07002238.hg.1	1.258405	0.001574	Up
TC0X000584.hg.1	1.568076	0.001576	Up
TC15002609.hg.1	1.230717	0.001584	Up
TC05002180.hg.1	− 1.289242	0.001587	Down
TC12001542.hg.1	1.248048	0.001594	Up
TC09002127.hg.1	− 1.251934	0.001597	Down
TC01005541.hg.1	1.349643	0.0016	Up
TC13000759.hg.1	1.325838	0.001602	Up
TC03002654.hg.1	1.573194	0.001605	Up
TC04000264.hg.1	− 1.242144	0.001607	Down
TC10001183.hg.1	1.254169	0.00161	Up
TC02001767.hg.1	1.501282	0.001613	Up
TC16001787.hg.1	− 1.23458	0.001615	Down
TC16001996.hg.1	− 1.413337	0.00162	Down
TC16001381.hg.1	− 1.250738	0.001626	Down
TC20001170.hg.1	− 1.215293	0.001628	Down
TC0X001367.hg.1	1.327188	0.001631	Up
TC05002924.hg.1	− 1.38887	0.001639	Down
TC01005172.hg.1	− 1.413469	0.001646	Down
TC06000650.hg.1	− 1.207228	0.001649	Down
TC02004514.hg.1	− 1.251876	0.001652	Down
TC14000850.hg.1	− 1.358753	0.001654	Down
TC04001198.hg.1	1.27254	0.001657	Up
TC0X000282.hg.1	− 1.384996	0.001659	Down
TC08000176.hg.1	− 1.262382	0.001662	Down
TC19002431.hg.1	− 1.271144	0.001667	Down
TC07000154.hg.1	1.214019	0.001672	Up
TC15001223.hg.1	1.41761	0.001677	Up
TC15000346.hg.1	1.607314	0.001685	Up
TC02003220.hg.1	− 1.220085	0.001693	Down
TC02001567.hg.1	− 1.514096	0.001696	Down
TC07003062.hg.1	1.473937	0.001701	Up
TC16001481.hg.1	− 1.256589	0.001703	Down
TC11002658.hg.1	− 1.243073	0.001706	Down
TC20000800.hg.1	− 1.22335	0.001714	Down
TC01004190.hg.1	− 1.280267	0.001719	Down
TC10000755.hg.1	− 1.500832	0.001727	Down
TC07002475.hg.1	1.335462	0.001748	Up
TC09000471.hg.1	− 1.258358	0.001758	Down
TC0X001618.hg.1	− 1.273233	0.001761	Down
TC01003152.hg.1	1.441204	0.001768	Up
TC20000841.hg.1	− 1.313791	0.001771	Down
TC05002975.hg.1	− 1.309682	0.001779	Down
TC08001847.hg.1	1.406037	0.001784	Up
TC02003913.hg.1	− 1.372422	0.001786	Down
TC10002891.hg.1	− 1.201915	0.001792	Down
TC19001852.hg.1	1.491505	0.001794	Up
TC03002962.hg.1	− 1.248919	0.001805	Down
TC01005988.hg.1	− 1.415789	0.001812	Down
TC01006043.hg.1	− 1.359798	0.001818	Down
TC14000381.hg.1	1.281079	0.00182	Up
TC12000580.hg.1	1.735542	0.001825	Up
TC01002098.hg.1	− 1.253355	0.001831	Down
TC02001032.hg.1	− 1.303562	0.001833	Down
TC04001271.hg.1	1.318074	0.001836	Up
TC11000769.hg.1	− 1.271684	0.001838	Down
TC03001559.hg.1	1.416685	0.001844	Up
TC09001810.hg.1	− 1.270957	0.001846	Down
TC08001514.hg.1	− 1.204519	0.001851	Down
TC14001530.hg.1	− 1.323404	0.001854	Down
TC02003388.hg.1	− 1.23358	0.001864	Down
TC0X000822.hg.1	− 1.718844	0.001867	Down
TC17002619.hg.1	− 1.211196	0.001877	Down
TC07000080.hg.1	− 1.269449	0.00188	Down
TC09002217.hg.1	− 1.319933	0.001882	Down
TC01003082.hg.1	1.682659	0.001894	Up
TC01003146.hg.1	1.682659	0.001894	Up
TC07001692.hg.1	1.305275	0.001901	Up
TC04001091.hg.1	− 1.284332	0.001903	Down
TC15001480.hg.1	− 1.38294	0.001911	Down
TC06002096.hg.1	− 1.296343	0.001914	Down
TC20001131.hg.1	1.239192	0.001916	Up
TC10001843.hg.1	− 1.279966	0.001919	Down
TC06002013.hg.1	1.468244	0.001924	Up
TC04001248.hg.1	1.498609	0.001927	Up
TC11002681.hg.1	1.284111	0.001929	Up
TC07003058.hg.1	− 1.28452	0.00194	Down
TC19002000.hg.1	− 1.245035	0.001942	Down
TC22000340.hg.1	1.589672	0.001945	Up
TC01002805.hg.1	1.202882	0.00195	Up
TC04000366.hg.1	1.409609	0.001953	Up
TC09000159.hg.1	− 1.312322	0.001958	Down
TC10001198.hg.1	− 1.357252	0.00196	Down
TC06000940.hg.1	1.265177	0.001963	Up
TC17001752.hg.1	1.334898	0.001965	Up
TC22001201.hg.1	− 2.439349	0.001973	Down
TC05003153.hg.1	− 1.240737	0.001976	Down
TC17002053.hg.1	− 1.244021	0.001978	Down
TC01003999.hg.1	− 1.700442	0.001986	Down
TC04002855.hg.1	− 1.268654	0.001989	Down
TC14000667.hg.1	1.399213	0.001991	Up
TC04001141.hg.1	− 1.525018	0.001994	Down
TC01005544.hg.1	1.237713	0.002002	Up
TC0X001406.hg.1	1.230021	0.002007	Up
TC05001662.hg.1	1.440258	0.00201	Up
TC11000282.hg.1	− 1.356168	0.002015	Down
TC21000954.hg.1	− 1.296443	0.002017	Down
TC08001129.hg.1	− 1.472614	0.002023	Down
TC06004012.hg.1	− 1.212754	0.002028	Down
TC17002825.hg.1	1.349775	0.00203	Up
TC06004016.hg.1	− 1.239402	0.002038	Down
TC10002884.hg.1	1.431763	0.002043	Up
TC05003136.hg.1	1.353844	0.002049	Up
TC12001421.hg.1	1.333251	0.002056	Up
TC15001280.hg.1	1.343707	0.00206	Up
TC15001283.hg.1	1.343707	0.00206	Up
TC14000739.hg.1	1.38166	0.002103	Up
TC10002474.hg.1	1.331327	0.002111	Up
TC14000601.hg.1	− 1.288544	0.002113	Down
TC11002989.hg.1	− 1.321635	0.002116	Down
TC0X001753.hg.1	− 1.205232	0.002119	Down
TC17000925.hg.1	1.272254	0.002121	Up
TC17000253.hg.1	1.345784	0.002124	Up
TC21000698.hg.1	− 1.374031	0.002137	Down
TC02001905.hg.1	1.328816	0.002147	Up
TC06002227.hg.1	− 1.498561	0.00215	Down
TC07000555.hg.1	− 1.337119	0.002152	Down
TC09002594.hg.1	1.339428	0.002155	Up
TC12001568.hg.1	1.341653	0.002157	Up
TC05001229.hg.1	1.360326	0.002165	Up
TC04001597.hg.1	− 1.331486	0.00217	Down
TC07002682.hg.1	− 1.221462	0.002173	Down
TC08001671.hg.1	1.488276	0.002176	Up
TC12002539.hg.1	− 1.275317	0.002178	Down
TC09002851.hg.1	− 1.333711	0.002181	Down
TC01000483.hg.1	1.552037	0.002186	Up
TC06003970.hg.1	− 1.368856	0.002189	Down
TC15002586.hg.1	− 1.340035	0.002191	Down
TC12002612.hg.1	− 1.256137	0.002196	Down
TC09001443.hg.1	− 1.207418	0.002212	Down
TC03001682.hg.1	1.260171	0.002215	Up
TC17000782.hg.1	− 1.204102	0.002217	Down
TC15000597.hg.1	1.213718	0.00222	Up
TC06003660.hg.1	− 1.237973	0.002222	Down
TC17002039.hg.1	1.340985	0.002225	Up
TC03002816.hg.1	− 1.247147	0.002228	Down
TC09000100.hg.1	− 1.377179	0.00223	Down
TC12002319.hg.1	− 1.211631	0.002235	Down
TC02002799.hg.1	− 1.264613	0.002241	Down
TC07000677.hg.1	− 1.215502	0.002246	Down
TC07000516.hg.1	1.289644	0.002248	Up
TC0X000501.hg.1	− 1.324115	0.002254	Down
TC16001448.hg.1	1.236904	0.002259	Up
TC07002912.hg.1	− 1.326523	0.002266	Down
TC10001062.hg.1	1.412222	0.002269	Up
TC10000761.hg.1	1.583303	0.002272	Up
TC05000214.hg.1	− 1.353026	0.002274	Down
TC09002468.hg.1	1.209262	0.002282	Up
TC12001658.hg.1	1.437781	0.002285	Up
TC03001924.hg.1	1.271901	0.00229	Up
TC03003246.hg.1	1.207269	0.002295	Up
TC14001678.hg.1	1.530119	0.0023	Up
TC03002629.hg.1	− 1.225872	0.002313	Down
TC20001519.hg.1	− 1.220912	0.002321	Down
TC01006051.hg.1	− 1.336503	0.002324	Down
TC06002665.hg.1	1.461845	0.002331	Up
TC02001522.hg.1	− 1.248588	0.002334	Down
TC12002293.hg.1	− 1.209515	0.002342	Down
TC21000628.hg.1	− 1.231307	0.002344	Down
TC12001919.hg.1	− 1.351643	0.002347	Down
TC02001799.hg.1	− 2.445831	0.00235	Down
TC17000742.hg.1	− 1.320632	0.002357	Down
TC04002002.hg.1	− 1.303665	0.00236	Down
TC0X001976.hg.1	1.267861	0.00237	Up
TC11002465.hg.1	− 1.277875	0.002383	Down
TC09001504.hg.1	− 1.312141	0.002388	Down
TC05000162.hg.1	1.294054	0.002391	Up
TC06003710.hg.1	− 1.344179	0.002399	Down
TC15002646.hg.1	1.2472	0.002401	Up
TC03002025.hg.1	− 1.217553	0.002417	Down
TC08000782.hg.1	1.360634	0.002422	Up
TC12001854.hg.1	− 1.235064	0.002427	Down
TC15000426.hg.1	− 1.229315	0.002435	Down
TC15002177.hg.1	1.279118	0.00244	Up
TC22001207.hg.1	− 1.203482	0.002451	Down
TC03002554.hg.1	− 1.220846	0.002458	Down
TC11000720.hg.1	1.488337	0.002461	Up
TC12003053.hg.1	1.261293	0.002471	Up
TC01003159.hg.1	1.455932	0.002479	Up
TC03000673.hg.1	− 1.307368	0.002484	Down
TC12001455.hg.1	− 1.316009	0.002487	Down
TC05000503.hg.1	− 1.223516	0.002497	Down
TC01002453.hg.1	1.461387	0.002508	Up
TC09002781.hg.1	1.429014	0.002518	Up
TC02000495.hg.1	1.291697	0.002529	Up
TC20001320.hg.1	1.297954	0.002531	Up
TC01004677.hg.1	− 1.285666	0.002534	Down
TC03000004.hg.1	− 1.234276	0.002539	Down
TC07000031.hg.1	1.223867	0.002557	Up
TC18000275.hg.1	− 1.277381	0.00256	Down
TC01003175.hg.1	1.43838	0.002565	Up
TC18000216.hg.1	− 1.286168	0.002567	Down
TC01006192.hg.1	1.268851	0.002573	Up
TC08000259.hg.1	− 1.344703	0.002583	Down
TC04001562.hg.1	1.450228	0.002593	Up
TC03003056.hg.1	1.215057	0.002601	Up
TC12001883.hg.1	1.569242	0.002609	Up
TC01003899.hg.1	− 1.28947	0.002617	Down
TC0Y000064.hg.1	1.200448	0.002628	Up
TC0Y000173.hg.1	1.200448	0.002628	Up
TC09000674.hg.1	− 1.2102	0.002635	Down
TC04000005.hg.1	− 1.295829	0.002638	Down
TC17002085.hg.1	− 1.281199	0.00264	Down
TC01001139.hg.1	1.508166	0.00265	Up
TC20001755.hg.1	1.352651	0.002656	Up
TC02002970.hg.1	− 1.429192	0.002658	Down
TC07002772.hg.1	1.373647	0.002661	Up
TC11001556.hg.1	− 1.206529	0.002666	Down
TC04002294.hg.1	− 1.401452	0.002671	Down
TC01005305.hg.1	1.356928	0.002676	Up
TC15001904.hg.1	− 1.272703	0.002682	Down
TC20000317.hg.1	1.430596	0.002687	Up
TC08000740.hg.1	1.402556	0.002692	Up
TC09001635.hg.1	− 1.276086	0.0027	Down
TC13001064.hg.1	− 1.26174	0.002702	Down
TC02003591.hg.1	1.299115	0.00271	Up
TC07002773.hg.1	1.331845	0.002721	Up
TC05002891.hg.1	− 1.259569	0.002726	Down
TC09002429.hg.1	− 1.205487	0.002728	Down
TC06001125.hg.1	− 1.232463	0.002734	Down
TC03002933.hg.1	− 1.211047	0.002744	Down
TC09002223.hg.1	− 1.202365	0.002746	Down
TC01004768.hg.1	− 1.224528	0.002757	Down
TC08000722.hg.1	1.365601	0.002762	Up
TC03001348.hg.1	− 1.239947	0.002767	Down
TC09002651.hg.1	− 1.69314	0.002778	Down
TC07002574.hg.1	− 1.24146	0.002788	Down
TC08000076.hg.1	1.404643	0.002793	Up
TC05000182.hg.1	1.301406	0.002804	Up
TC0X000127.hg.1	− 1.20556	0.002809	Down
TC03000962.hg.1	− 1.382564	0.002819	Down
TC07000246.hg.1	− 1.267632	0.002827	Down
TC07000320.hg.1	1.341276	0.002835	Up
TC11002734.hg.1	1.297811	0.002843	Up
TC17001691.hg.1	− 1.271637	0.002848	Down
TC01001837.hg.1	1.550832	0.00285	Up
TC20000915.hg.1	− 1.232658	0.002863	Down
TC02003364.hg.1	− 1.266053	0.002871	Down
TC0X001104.hg.1	1.359394	0.002889	Up
TC01000711.hg.1	− 1.216828	0.002892	Down
TC17000989.hg.1	− 1.21244	0.002894	Down
TC02002022.hg.1	1.263206	0.002897	Up
TC07002067.hg.1	− 1.31306	0.0029	Down
TC07001289.hg.1	− 1.351691	0.002902	Down
TC01000295.hg.1	1.305782	0.002905	Up
TC06000662.hg.1	1.480994	0.002907	Up
TC19002432.hg.1	− 1.286462	0.00291	Down
TC14002087.hg.1	− 1.270823	0.002918	Down
TC05002826.hg.1	1.273654	0.00292	Up
TC04001876.hg.1	− 1.318727	0.002936	Down
TC07002081.hg.1	− 1.319864	0.002939	Down
TC20001277.hg.1	1.278604	0.002946	Up
TC05000501.hg.1	1.274401	0.002962	Up
TC10000590.hg.1	1.370535	0.002977	Up
TC15001485.hg.1	− 1.291058	0.002993	Down
TC15002487.hg.1	− 1.367271	0.003006	Down
TC21000774.hg.1	− 1.289989	0.003009	Down
TC02002679.hg.1	− 1.293013	0.003011	Down
TC07001604.hg.1	1.354263	0.003014	Up
TC12001252.hg.1	1.447457	0.003016	Up
TC13001458.hg.1	− 1.207147	0.003019	Down
TC09002644.hg.1	− 1.26481	0.003022	Down
TC0X001878.hg.1	− 1.209099	0.003024	Down
TC0X001302.hg.1	1.569258	0.003027	Up
TC18000294.hg.1	1.260461	0.003045	Up
TC08000507.hg.1	− 1.315641	0.003055	Down
TC08000970.hg.1	− 1.224392	0.003058	Down
TC05002688.hg.1	− 1.2392	0.00306	Down
TC11003229.hg.1	− 1.251246	0.003063	Down
TC09002325.hg.1	− 1.270503	0.003076	Down
TC10002238.hg.1	− 1.329862	0.003081	Down
TC06001064.hg.1	− 1.274065	0.003092	Down
TC02003391.hg.1	− 1.362446	0.003099	Down
TC10000133.hg.1	− 1.238252	0.003103	Down
TC10000136.hg.1	− 1.238252	0.003103	Down
TC03002201.hg.1	− 1.331047	0.003112	Down
TC07001869.hg.1	− 1.488884	0.003118	Down
TC15001493.hg.1	− 1.20754	0.003125	Down
TC15002311.hg.1	− 1.221615	0.003136	Down
TC07001788.hg.1	− 1.398937	0.003143	Down
TC06003354.hg.1	− 1.228478	0.003146	Down
TC07002240.hg.1	1.460799	0.003151	Up
TC01000348.hg.1	− 1.280176	0.003162	Down
TC05000429.hg.1	− 1.204846	0.003172	Down
TC10002375.hg.1	− 1.214255	0.003175	Down
TC11001740.hg.1	1.307581	0.003177	Up
TC11000061.hg.1	1.501956	0.00318	Up
TC16000236.hg.1	1.306824	0.003203	Up
TC07001599.hg.1	− 1.353408	0.003211	Down
TC08001580.hg.1	− 1.29275	0.003219	Down
TC20000677.hg.1	− 1.209774	0.003221	Down
TC0X001145.hg.1	1.32182	0.003227	Up
TC19000162.hg.1	− 1.296602	0.003232	Down
TC0X001119.hg.1	1.23651	0.003258	Up
TC16001238.hg.1	1.548063	0.00326	Up
TC04000904.hg.1	− 1.325403	0.003281	Down
TC01004933.hg.1	1.296042	0.003284	Up
TC03002799.hg.1	1.233829	0.003286	Up
TC17001438.hg.1	− 1.282082	0.003307	Down
TC12002384.hg.1	1.229617	0.003315	Up
TC06003692.hg.1	− 1.256837	0.003323	Down
TC06001254.hg.1	− 1.229231	0.003328	Down
TC21000398.hg.1	− 1.279981	0.003333	Down
TC06000858.hg.1	1.611132	0.003341	Up
TC15001164.hg.1	− 1.235207	0.003343	Down
TC01000551.hg.1	1.249583	0.003348	Up
TC12003054.hg.1	1.220865	0.003354	Up
TC18000207.hg.1	1.207556	0.003359	Up
TC16001555.hg.1	− 1.235816	0.003369	Down
TC07000470.hg.1	1.268777	0.003372	Up
TC06001808.hg.1	1.248183	0.003385	Up
TC01000364.hg.1	− 1.330246	0.003387	Down
TC12000919.hg.1	1.563172	0.00339	Up
TC05001064.hg.1	1.565417	0.003393	Up
TC17002281.hg.1	− 1.200596	0.003411	Down
TC10000082.hg.1	− 1.375037	0.00346	Down
TC02003240.hg.1	− 1.307516	0.003481	Down
TC04001241.hg.1	− 1.290744	0.003491	Down
TC02004324.hg.1	− 1.232763	0.003494	Down
TC10002413.hg.1	− 1.245515	0.003515	Down
TC16001946.hg.1	− 1.22412	0.003525	Down
TC02002923.hg.1	− 1.276668	0.003528	Down
TC09002766.hg.1	− 1.258491	0.003535	Down
TC09000642.hg.1	− 1.347036	0.003538	Down
TC02001155.hg.1	− 1.210161	0.003548	Down
TC17000690.hg.1	− 1.278329	0.003556	Down
TC22000690.hg.1	− 1.201581	0.003564	Down
TC03001758.hg.1	− 1.260934	0.003566	Down
TC10002858.hg.1	− 1.261472	0.0036	Down
TC01001573.hg.1	− 1.31615	0.003608	Down
TC12000943.hg.1	− 1.20209	0.003613	Down
TC07000478.hg.1	− 1.269749	0.003626	Down
TC22001006.hg.1	− 1.20719	0.003636	Down
TC21000941.hg.1	− 1.364074	0.003655	Down
TC07002774.hg.1	− 1.224753	0.00366	Down
TC06003965.hg.1	− 1.238846	0.003665	Down
TC0Y000049.hg.1	− 1.363073	0.003675	Down
TC04000236.hg.1	− 1.212799	0.003681	Down
TC0X001770.hg.1	− 1.33727	0.003691	Down
TC06003404.hg.1	− 1.327154	0.003694	Down
TC05001562.hg.1	− 1.380095	0.003699	Down
TC05001222.hg.1	− 1.267421	0.003725	Down
TC01005060.hg.1	− 1.314409	0.003727	Down
TC03000359.hg.1	− 1.202882	0.003751	Down
TC08001194.hg.1	− 1.424464	0.003753	Down
TC09001332.hg.1	− 1.371182	0.003782	Down
TC07001668.hg.1	− 1.221176	0.003816	Down
TC06000609.hg.1	− 1.2365	0.003834	Down
TC14002171.hg.1	− 1.305933	0.003839	Down
TC05003054.hg.1	− 1.216461	0.003841	Down
TC12000990.hg.1	− 1.45711	0.003854	Down
TC18000510.hg.1	− 1.260881	0.003862	Down
TC04000456.hg.1	− 1.263786	0.00388	Down
TC01000871.hg.1	− 1.21274	0.003896	Down
TC16000865.hg.1	− 1.285648	0.003899	Down
TC06002606.hg.1	− 1.49097	0.003927	Down
TC22000272.hg.1	− 1.554161	0.003932	Down
TC06002746.hg.1	− 1.202909	0.00394	Down
TC0Y000257.hg.1	− 1.281043	0.003945	Down
TC18000540.hg.1	− 1.251836	0.003948	Down
TC08002048.hg.1	− 1.207051	0.003966	Down
TC17002700.hg.1	− 1.235688	0.003974	Down
TC05001722.hg.1	− 1.273013	0.003982	Down
TC01005183.hg.1	− 1.227973	0.003987	Down
TC12002485.hg.1	− 1.260362	0.004	Down
TC10001548.hg.1	− 1.301554	0.004002	Down
TC13001206.hg.1	− 1.467406	0.004005	Down
TC11003065.hg.1	− 1.232606	0.004013	Down
TC10000091.hg.1	− 1.407866	0.004018	Down
TC06004002.hg.1	− 1.324321	0.00402	Down
TC02004420.hg.1	− 1.245413	0.004057	Down
TC02002409.hg.1	− 1.296558	0.004059	Down
TC05002084.hg.1	− 1.429991	0.004067	Down
TC03000687.hg.1	− 1.368897	0.004096	Down
TC05002736.hg.1	− 1.269686	0.004101	Down
TC11000172.hg.1	− 1.381513	0.004117	Down
TC04000860.hg.1	− 1.20631	0.004197	Down
TC13001692.hg.1	− 1.204469	0.004202	Down
TC03002000.hg.1	− 1.245183	0.00421	Down
TC0X000016.hg.1	− 1.216418	0.00422	Down
TC01005274.hg.1	− 1.212808	0.004223	Down
TC17001702.hg.1	− 1.257121	0.004264	Down
TC07002787.hg.1	− 1.2704	0.004275	Down
TC11001982.hg.1	− 1.215692	0.004283	Down
TC11002457.hg.1	− 1.224026	0.004285	Down
TC21000490.hg.1	− 1.222362	0.004298	Down
TC02001213.hg.1	− 1.275065	0.004309	Down
TC05002802.hg.1	− 1.454294	0.004316	Down
TC02002613.hg.1	− 1.202558	0.004342	Down
TC07002265.hg.1	− 1.303624	0.00435	Down
TC05002711.hg.1	− 1.278714	0.00436	Down
TC09000458.hg.1	− 1.295586	0.004368	Down
TC15001598.hg.1	− 1.273945	0.004373	Down
TC08000107.hg.1	− 1.284986	0.004389	Down
TC12002849.hg.1	− 1.258618	0.004394	Down
TC06002956.hg.1	− 1.208955	0.004397	Down
TC10000426.hg.1	− 1.257512	0.004417	Down
TC07000389.hg.1	− 1.257421	0.004438	Down
TC16001097.hg.1	− 1.300925	0.004443	Down
TC07002791.hg.1	− 1.206028	0.004459	Down
TC19001288.hg.1	− 1.237947	0.004464	Down
TC16000146.hg.1	− 1.233673	0.004467	Down
TC01002560.hg.1	− 1.207402	0.004482	Down
TC10002728.hg.1	− 1.272287	0.004506	Down
TC12000184.hg.1	− 1.207964	0.004508	Down
TC10001956.hg.1	− 1.201634	0.004513	Down
TC10001530.hg.1	− 1.212456	0.00456	Down
TC17002899.hg.1	− 1.208538	0.004573	Down
TC0X000632.hg.1	− 1.3172	0.004581	Down
TC17001547.hg.1	− 1.20639	0.004589	Down
TC04000311.hg.1	− 1.204792	0.004599	Down
TC20001506.hg.1	− 1.26979	0.004609	Down
TC06004015.hg.1	− 1.26321	0.004612	Down
TC01004440.hg.1	− 1.222055	0.004622	Down
TC01002542.hg.1	− 1.318907	0.004628	Down
TC18000718.hg.1	− 1.231172	0.004638	Down
TC04002390.hg.1	− 1.292294	0.004716	Down
TC04002908.hg.1	− 1.22141	0.004724	Down
TC01001491.hg.1	− 1.255189	0.004731	Down
TC19000841.hg.1	− 1.300943	0.004755	Down
TC01002756.hg.1	− 1.426354	0.00476	Down
TC20000309.hg.1	− 1.304516	0.004765	Down
TC11002990.hg.1	− 1.235653	0.00477	Down
TC03001403.hg.1	− 1.314851	0.004799	Down
TC06003498.hg.1	− 1.355157	0.004802	Down
TC09002774.hg.1	− 1.224146	0.00482	Down
TC10000250.hg.1	− 1.330922	0.004846	Down
TC16000333.hg.1	− 1.201118	0.004848	Down
TC22001283.hg.1	− 1.280786	0.004859	Down
TC10001915.hg.1	− 1.228546	0.004864	Down
TC11000196.hg.1	− 1.265687	0.004869	Down
TC02004125.hg.1	− 1.231632	0.004898	Down
TC15002307.hg.1	− 1.255684	0.0049	Down
TC03003205.hg.1	− 1.249546	0.004913	Down
TC01002776.hg.1	− 1.316093	0.004939	Down
TC03001745.hg.1	− 1.299476	0.004944	Down
TC06003126.hg.1	− 1.286256	0.004952	Down
TC01002773.hg.1	− 1.218511	0.004965	Down
TC02002927.hg.1	− 1.311688	0.004983	Down
TC01004326.hg.1	− 1.204881	0.004986	Down
TC21000729.hg.1	− 1.200973	0.004988	Down
TC22000110.hg.1	− 1.205007	0.004999	Down
TC09001132.hg.1	− 1.271309	0.005012	Down
TC10000232.hg.1	− 1.248461	0.005017	Down
TC05003302.hg.1	− 1.255306	0.005019	Down
TC20001224.hg.1	− 1.32063	0.005025	Down
TC01005383.hg.1	− 1.256589	0.005027	Down
TC0X001718.hg.1	− 1.226406	0.00504	Down
TC14000707.hg.1	− 1.204295	0.005058	Down
TC14001715.hg.1	− 1.346526	0.005061	Down
TC07002764.hg.1	− 1.200408	0.005079	Down
TC06003509.hg.1	− 1.230654	0.005084	Down
TC05002436.hg.1	− 1.273183	0.005118	Down
TC05000078.hg.1	− 1.23626	0.005121	Down
TC01006128.hg.1	− 1.328935	0.005126	Down
TC06003211.hg.1	− 1.277803	0.005157	Down
TC10000904.hg.1	− 1.247917	0.00516	Down
TC01001668.hg.1	− 1.212246	0.005206	Down
TC21000493.hg.1	− 1.236162	0.005232	Down
TC09000456.hg.1	− 1.266448	0.00524	Down
TC09002485.hg.1	− 1.263839	0.005256	Down
TC20001556.hg.1	− 1.279838	0.005284	Down
TC02002768.hg.1	− 1.253353	0.005287	Down
TC10002113.hg.1	− 1.217268	0.005328	Down
TC12001434.hg.1	− 1.297937	0.005333	Down
TC05001581.hg.1	− 1.260177	0.005344	Down
TC15002099.hg.1	− 1.266836	0.005352	Down
TC10001912.hg.1	− 1.349501	0.005391	Down
TC11002900.hg.1	− 1.284679	0.005393	Down
TC01005460.hg.1	− 1.207368	0.005401	Down
TC02001846.hg.1	− 1.402528	0.005406	Down
TC09000907.hg.1	− 1.24214	0.005409	Down
TC0X001572.hg.1	− 1.33578	0.005411	Down
TC09001363.hg.1	− 1.240359	0.005414	Down
TC10001686.hg.1	− 1.295313	0.005458	Down
TC08000285.hg.1	− 1.288083	0.005468	Down
TC07000767.hg.1	− 1.290819	0.005474	Down
TC13000606.hg.1	− 1.330779	0.005479	Down
TC04001792.hg.1	− 1.242708	0.005481	Down
TC02003857.hg.1	− 1.268582	0.005492	Down
TC01003317.hg.1	− 1.440286	0.005531	Down
TC12000588.hg.1	− 1.213828	0.005541	Down
TC20001554.hg.1	− 1.207139	0.005544	Down
TC07001657.hg.1	− 1.419211	0.005577	Down
TC09002148.hg.1	− 1.279893	0.00559	Down
TC15001221.hg.1	− 1.210747	0.005658	Down
TC11001828.hg.1	− 1.200582	0.00566	Down
TC13000062.hg.1	− 1.283458	0.005697	Down
TC05001353.hg.1	− 1.216892	0.005699	Down
TC01004219.hg.1	− 1.205464	0.005702	Down
TC01005835.hg.1	− 1.269379	0.005725	Down
TC06000930.hg.1	− 1.262508	0.00573	Down
TC03002911.hg.1	− 1.233783	0.005749	Down
TC02000415.hg.1	− 1.219812	0.005759	Down
TC11003125.hg.1	− 1.221046	0.005764	Down
TC01000579.hg.1	− 1.203049	0.005803	Down
TC14001295.hg.1	− 1.214363	0.005808	Down
TC05002238.hg.1	− 1.208419	0.005837	Down
TC04000662.hg.1	− 1.270677	0.005852	Down
TC02002347.hg.1	− 1.22743	0.005858	Down
TC17000164.hg.1	− 1.280195	0.005871	Down
TC08001315.hg.1	− 1.408884	0.005894	Down
TC03000982.hg.1	− 1.239878	0.005896	Down
TC11002959.hg.1	− 1.229904	0.005933	Down
TC14001177.hg.1	− 1.267289	0.005938	Down
TC11003265.hg.1	− 1.234478	0.005943	Down
TC01005831.hg.1	− 1.322234	0.005946	Down
TC09001793.hg.1	− 1.214475	0.005951	Down
TC01003533.hg.1	− 1.45726	0.00598	Down
TC06002968.hg.1	− 1.357034	0.00599	Down
TC0X000285.hg.1	− 1.203534	0.006068	Down
TC03003239.hg.1	− 1.234383	0.006086	Down
TC01004831.hg.1	− 1.238529	0.006107	Down
TC22001104.hg.1	− 1.213074	0.00614	Down
TC20001570.hg.1	− 1.20816	0.006172	Down
TC05000703.hg.1	− 1.311244	0.006174	Down
TC18000785.hg.1	− 1.224874	0.006179	Down
TC09002372.hg.1	− 1.257884	0.006182	Down
TC04000837.hg.1	− 1.230161	0.006195	Down
TC11000065.hg.1	− 1.217834	0.006275	Down
TC21000071.hg.1	− 1.206307	0.00628	Down
TC13001054.hg.1	− 1.273984	0.006291	Down
TC02003343.hg.1	− 1.214008	0.00634	Down
TC12002692.hg.1	− 1.289651	0.006353	Down
TC01005539.hg.1	− 1.21791	0.006371	Down
TC07000299.hg.1	− 1.266678	0.006379	Down
TC12000625.hg.1	− 1.265515	0.006384	Down
TC09002445.hg.1	− 1.334389	0.006413	Down
TC05001346.hg.1	− 1.231796	0.006493	Down
TC12001821.hg.1	− 1.242271	0.006511	Down
TC08000954.hg.1	− 1.26309	0.00655	Down
TC12000683.hg.1	− 1.311043	0.006566	Down
TC17002263.hg.1	− 1.2066	0.006574	Down
TC01001760.hg.1	− 1.277558	0.006592	Down
TC17000557.hg.1	− 1.322598	0.006597	Down
TC20001196.hg.1	− 1.31133	0.0066	Down
TC04001967.hg.1	− 1.290136	0.006618	Down
TC14000795.hg.1	− 1.300583	0.006633	Down
TC12000745.hg.1	− 1.336139	0.006672	Down
TC17001160.hg.1	− 1.232074	0.006688	Down
TC09002037.hg.1	− 1.25914	0.006701	Down
TC07000189.hg.1	− 1.284554	0.006711	Down
TC10002162.hg.1	− 1.331341	0.006729	Down
TC10001758.hg.1	− 1.33558	0.006732	Down
TC6_ssto_hap7000179.hg.1	− 1.266774	0.006776	Down
TC05001609.hg.1	− 1.219982	0.006802	Down
TC09000397.hg.1	− 1.208158	0.006825	Down
TC01000008.hg.1	− 1.260407	0.006838	Down
TC05002179.hg.1	− 1.24681	0.006955	Down
TC06003468.hg.1	− 1.237969	0.007002	Down
TC07000766.hg.1	− 1.246781	0.007038	Down
TC03002074.hg.1	− 1.286913	0.007049	Down
TC06002573.hg.1	− 1.200492	0.007087	Down
TC17002315.hg.1	− 1.224284	0.007119	Down
TC17002681.hg.1	− 1.215864	0.007147	Down
TC08002507.hg.1	− 1.219674	0.007209	Down
TC07001642.hg.1	− 1.262919	0.007238	Down
TC04002802.hg.1	− 1.281658	0.007272	Down
TC07000507.hg.1	− 1.319983	0.007285	Down
TC03000233.hg.1	− 1.267284	0.0073	Down
TC17000344.hg.1	− 1.230796	0.007318	Down
TC10000974.hg.1	− 1.235907	0.007324	Down
TC01002173.hg.1	− 1.204552	0.007339	Down
TC03000641.hg.1	− 1.205138	0.007362	Down
TC01004748.hg.1	− 1.265677	0.007451	Down
TC11001732.hg.1	− 1.382737	0.007461	Down
TC07002298.hg.1	− 1.229053	0.007471	Down
TC02000198.hg.1	− 1.241303	0.007479	Down
TC19000822.hg.1	− 1.291223	0.007487	Down
TC05002943.hg.1	− 1.222683	0.00749	Down
TC05000859.hg.1	− 1.310438	0.007529	Down
TC01006140.hg.1	− 1.291211	0.00756	Down
TC02001517.hg.1	− 1.226001	0.007575	Down
TC02001732.hg.1	− 1.211515	0.007601	Down
TC17002605.hg.1	− 1.203515	0.007619	Down
TC02004353.hg.1	− 1.446996	0.007622	Down

Probe set ID	Fold change	p-value	Gene feature
(C) *Genes up/down-regulated in non-purified lung TCs*^*SV40*^ *compared with non-purified primary lung TCs*			
TC03003114.hg.1	4.734104	2.70E−05	Up
TC17001801.hg.1	− 2.581599	3.00E−05	Down
TC11002894.hg.1	3.769187	3.20E−05	Up
TC04000160.hg.1	− 2.019509	3.50E−05	Down
TC20000952.hg.1	− 2.001725	3.80E−05	Down
TC04002890.hg.1	2.318185	4.00E−05	Up
TC11003109.hg.1	5.927567	4.30E−05	Up
TC02000205.hg.1	− 1.864365	4.50E−05	Down
TC12000558.hg.1	− 1.77743	4.80E−05	Down
TC09000971.hg.1	− 1.924593	5.10E−05	Down
TC0X001292.hg.1	− 3.110776	5.30E−05	Down
TC08000385.hg.1	− 1.758147	5.60E−05	Down
TC02004814.hg.1	− 1.730004	5.80E−05	Down
TC06001978.hg.1	− 2.037057	6.10E−05	Down
TC12001286.hg.1	− 1.823703	6.40E−05	Down
TC08000302.hg.1	− 2.018038	6.60E−05	Down
TC07001784.hg.1	− 1.938506	6.90E−05	Down
TC12000927.hg.1	− 2.156997	7.10E−05	Down
TC15000452.hg.1	− 2.013654	7.40E−05	Down
TC11002382.hg.1	− 3.896927	7.70E−05	Down
TC02001940.hg.1	− 1.982875	7.90E−05	Down
TC09001648.hg.1	− 2.074788	8.20E−05	Down
TC02001602.hg.1	− 1.697784	8.40E−05	Down
TC06000639.hg.1	− 2.003858	8.70E−05	Down
TC04002560.hg.1	2.166732	9.00E−05	Up
TC17001773.hg.1	− 1.582128	9.20E−05	Down
TC13000783.hg.1	− 2.527727	9.50E−05	Down
TC0X001624.hg.1	− 1.937361	9.70E−05	Down
TC05001593.hg.1	− 1.926476	1.00E−04	Down
TC19001567.hg.1	1.692676	0.000103	Up
TC02002456.hg.1	− 1.806913	0.000105	Down
TC08002421.hg.1	− 2.163126	0.000108	Down
TC09001898.hg.1	− 1.436061	0.00011	Down
TC0X000450.hg.1	− 1.484801	0.000113	Down
TC02003744.hg.1	1.585428	0.000116	Up
TC07002463.hg.1	1.777811	0.000118	Up
TC07001655.hg.1	− 1.503467	0.000121	Down
TC22000156.hg.1	1.898289	0.000123	Up
TC16000648.hg.1	− 2.164873	0.000126	Down
TC04002642.hg.1	− 1.587573	0.000129	Down
TC16001289.hg.1	− 1.766618	0.000131	Down
TC06000568.hg.1	− 1.73117	0.000134	Down
TC20000650.hg.1	− 1.835321	0.000136	Down
TC0X001889.hg.1	1.580745	0.000139	Up
TC04001945.hg.1	− 1.643053	0.000142	Down
TC11001010.hg.1	− 1.603866	0.000144	Down
TC03001841.hg.1	− 1.766003	0.000147	Down
TC05003313.hg.1	1.920567	0.000149	Up
TC05001142.hg.1	− 1.371777	0.000152	Down
TC02000396.hg.1	− 1.829949	0.000155	Down
TC12000231.hg.1	− 1.550803	0.000157	Down
TC17000491.hg.1	− 1.592587	0.00016	Down
TC0X001430.hg.1	− 1.820415	0.000162	Down
TC16002030.hg.1	− 1.524415	0.000165	Down
TC02001836.hg.1	− 1.49041	0.000168	Down
TC07002531.hg.1	1.715119	0.00017	Up
TC17002479.hg.1	1.76047	0.000173	Up
TC04001635.hg.1	− 2.303785	0.000175	Down
TC01003308.hg.1	− 1.900128	0.000178	Down
TC08002253.hg.1	− 2.893756	0.000181	Down
TC06000705.hg.1	− 1.731076	0.000183	Down
TC08000801.hg.1	− 1.729942	0.000186	Down
TC15001184.hg.1	− 1.439815	0.000188	Down
TC0X001410.hg.1	− 1.581381	0.000191	Down
TC16001066.hg.1	− 1.31385	0.000194	Down
TC0X001822.hg.1	1.439398	0.000196	Up
TC22001175.hg.1	− 1.542513	0.000199	Down
TC05002512.hg.1	− 1.48484	0.000201	Down
TC12001449.hg.1	− 1.863709	0.000204	Down
TC01001501.hg.1	− 1.466269	0.000207	Down
TC20000373.hg.1	− 1.596942	0.000209	Down
TC01003990.hg.1	− 1.514826	0.000212	Down
TC09000096.hg.1	− 1.937731	0.000214	Down
TC06000824.hg.1	− 1.586137	0.000217	Down
TC10002723.hg.1	− 1.693797	0.00022	Down
TC06004042.hg.1	− 1.463012	0.000222	Down
TC19000767.hg.1	− 1.80746	0.000225	Down
TC15001604.hg.1	− 1.573292	0.000227	Down
TC0X000985.hg.1	− 2.24906	0.00023	Down
TC0X001210.hg.1	− 1.648953	0.000233	Down
TC08002510.hg.1	2.254553	0.000235	Up
TC12000633.hg.1	− 1.764414	0.000238	Down
TC06003416.hg.1	− 1.389758	0.00024	Down
TC09000963.hg.1	− 1.4781	0.000243	Down
TC15001326.hg.1	− 1.537491	0.000246	Down
TC01004763.hg.1	1.400232	0.000248	Up
TC12002858.hg.1	1.534662	0.000251	Up
TC05002279.hg.1	− 1.509417	0.000253	Down
TC0X001136.hg.1	− 1.444487	0.000256	Down
TC08000652.hg.1	− 1.577232	0.000259	Down
TC11002486.hg.1	− 1.651639	0.000261	Down
TC07003059.hg.1	1.417719	0.000264	Up
TC02000955.hg.1	− 1.666162	0.000266	Down
TC14001892.hg.1	− 1.606681	0.000269	Down
TC17001656.hg.1	− 1.472658	0.000272	Down
TC06002227.hg.1	− 1.627176	0.000274	Down
TC05000998.hg.1	− 1.408795	0.000277	Down
TC09001602.hg.1	− 1.887078	0.000279	Down
TC09001534.hg.1	− 1.467576	0.000282	Down
TC02004275.hg.1	− 1.320789	0.000285	Down
TC17000728.hg.1	− 2.065452	0.000287	Down
TC06000725.hg.1	1.440025	0.00029	Up
TC10002919.hg.1	− 1.600614	0.000292	Down
TC12000805.hg.1	− 1.56655	0.000295	Down
TC10000686.hg.1	− 1.469338	0.000298	Down
TC0X001158.hg.1	− 1.926968	3.00E−04	Down
TC09001936.hg.1	− 1.453724	0.000303	Down
TC16001498.hg.1	1.666085	0.000305	Up
TC12002493.hg.1	1.355038	0.000308	Up
TC10000439.hg.1	− 1.562003	0.000311	Down
TC0X001411.hg.1	− 1.587704	0.000313	Down
TC10000866.hg.1	− 1.575569	0.000318	Down
TC05002034.hg.1	− 1.483066	0.000321	Down
TC0Y000064.hg.1	1.420454	0.000325	Up
TC0Y000173.hg.1	1.420454	0.000325	Up
TC08001864.hg.1	2.861562	0.000329	Up
TC19000163.hg.1	− 1.609154	0.000331	Down
TC09000516.hg.1	− 1.559053	0.000334	Down
TC14002059.hg.1	1.551644	0.000337	Up
TC11002784.hg.1	1.820896	0.000339	Up
TC07002052.hg.1	− 1.561394	0.000342	Down
TC03002116.hg.1	− 1.474893	0.000344	Down
TC11002476.hg.1	− 1.57355	0.000347	Down
TC06002303.hg.1	− 1.518545	0.00035	Down
TC14001731.hg.1	1.403751	0.000352	Up
TC04002294.hg.1	− 1.448062	0.000355	Down
TC01003510.hg.1	− 1.493469	0.000357	Down
TC05001562.hg.1	− 1.537085	0.00036	Down
TC01002976.hg.1	− 1.566265	0.000362	Down
TC02001432.hg.1	− 1.55349	0.000365	Down
TC16001854.hg.1	− 1.350821	0.000368	Down
TC21000698.hg.1	− 1.401198	0.00037	Down
TC03000195.hg.1	− 1.616867	0.000373	Down
TC06001651.hg.1	− 1.50443	0.000375	Down
TC06001365.hg.1	− 1.809445	0.000378	Down
TC07001706.hg.1	− 1.618734	0.000382	Down
TC07001709.hg.1	− 1.618734	0.000382	Down
TC15002247.hg.1	1.354504	0.000386	Up
TC21000931.hg.1	− 1.420416	0.000391	Down
TC04002443.hg.1	− 1.551184	0.000394	Down
TC09000586.hg.1	− 1.554678	0.000396	Down
TC14000620.hg.1	− 1.494772	0.000399	Down
TC06002402.hg.1	1.646644	0.000401	Up
TC12000088.hg.1	1.444149	0.000404	Up
TC01002798.hg.1	− 1.895656	0.000407	Down
TC05002818.hg.1	− 1.513562	0.000409	Down
TC03001056.hg.1	− 1.606915	0.000412	Down
TC01002746.hg.1	− 1.453698	0.000414	Down
TC02003041.hg.1	− 1.421703	0.000417	Down
TC04000811.hg.1	− 1.391548	0.00042	Down
TC09002304.hg.1	− 1.275207	0.000422	Down
TC05002800.hg.1	− 1.443218	0.000425	Down
TC03003057.hg.1	1.435368	0.000427	Up
TC11002810.hg.1	1.338827	0.00043	Up
TC08002059.hg.1	1.653874	0.000433	Up
TC0X002006.hg.1	− 1.347272	0.000435	Down
TC04001524.hg.1	− 1.559985	0.000438	Down
TC22001053.hg.1	− 1.465136	0.00044	Down
TC05000688.hg.1	− 1.557631	0.000443	Down
TC08002396.hg.1	− 1.266228	0.000446	Down
TC03001571.hg.1	− 1.575772	0.000448	Down
TC09002074.hg.1	1.663248	0.000451	Up
TC18000864.hg.1	1.491055	0.000453	Up
TC02001429.hg.1	− 1.512663	0.000456	Down
TC22000400.hg.1	− 1.474029	0.000459	Down
TC04001675.hg.1	1.523103	0.000461	Up
TC05000183.hg.1	− 1.591351	0.000466	Down
TC08000450.hg.1	− 1.754444	0.000469	Down
TC22001417.hg.1	− 1.575065	0.000477	Down
TC16001991.hg.1	− 1.30884	0.000479	Down
TC02004784.hg.1	− 1.291479	0.000482	Down
TC12003094.hg.1	− 1.450031	0.000487	Down
TC05002434.hg.1	1.258242	0.00049	Up
TC06002096.hg.1	− 1.459176	0.000492	Down
TC09000471.hg.1	− 1.399173	0.000495	Down
TC08002500.hg.1	− 1.277475	0.000498	Down
TC21000887.hg.1	1.340516	5.00E−04	Up
TC04001473.hg.1	− 1.527189	0.000503	Down
TC01000642.hg.1	− 1.492463	0.000505	Down
TC07001788.hg.1	− 1.686158	0.000508	Down
TC02003970.hg.1	− 1.440374	0.000511	Down
TC07000478.hg.1	− 1.476748	0.000513	Down
TC01005360.hg.1	1.315026	0.000516	Up
TC08002335.hg.1	− 1.716035	0.000518	Down
TC14002181.hg.1	− 1.260915	0.000521	Down
TC01002620.hg.1	− 1.443838	0.000526	Down
TC16001156.hg.1	− 1.403474	0.000531	Down
TC11001288.hg.1	− 1.336498	0.000534	Down
TC12003194.hg.1	− 1.56187	0.000537	Down
TC0X002258.hg.1	− 1.47544	0.000539	Down
TC05001001.hg.1	− 1.403589	0.000542	Down
TC04002346.hg.1	1.340139	0.000544	Up
TC06002606.hg.1	− 1.808218	0.000547	Down
TC14000667.hg.1	1.35603	0.00055	Up
TC03003257.hg.1	− 1.541547	0.000552	Down
TC13001029.hg.1	1.266449	0.000555	Up
TC01006195.hg.1	− 1.266774	0.000557	Down
TC12001995.hg.1	− 1.530295	0.00056	Down
TC12001434.hg.1	− 1.326576	0.000563	Down
TC10000232.hg.1	− 1.308551	0.000565	Down
TC03003056.hg.1	1.356981	0.000568	Up
TC01006074.hg.1	− 1.330658	0.00057	Down
TC11003147.hg.1	− 1.269907	0.000573	Down
TC03002679.hg.1	1.353352	0.000576	Up
TC0X001094.hg.1	− 1.384223	0.000578	Down
TC09002823.hg.1	1.490093	0.000581	Up
TC13001025.hg.1	1.345303	0.000586	Up
TC09000043.hg.1	1.295164	0.000589	Up
TC10001021.hg.1	− 1.339644	0.000591	Down
TC12001052.hg.1	− 1.639168	0.000594	Down
TC04000338.hg.1	− 1.30348	0.000596	Down
TC02001799.hg.1	− 2.28736	0.000602	Down
TC14000739.hg.1	1.501284	0.000604	Up
TC04000508.hg.1	− 1.464186	0.000607	Down
TC09001812.hg.1	− 1.251376	0.000609	Down
TC08001514.hg.1	− 1.447493	0.000612	Down
TC02003364.hg.1	− 1.343783	0.000615	Down
TC0X001068.hg.1	− 1.274558	0.000617	Down
TC09001015.hg.1	− 1.353939	0.000622	Down
TC18000408.hg.1	1.509875	0.000625	Up
TC10001530.hg.1	− 1.256877	0.00063	Down
TC06003710.hg.1	− 1.416403	0.000633	Down
TC12002688.hg.1	− 1.639852	0.000635	Down
TC20000045.hg.1	− 1.459984	0.000638	Down
TC03002103.hg.1	− 1.492607	0.000641	Down
TC0X001762.hg.1	− 1.384395	0.000646	Down
TC01001291.hg.1	1.372502	0.000648	Up
TC09002148.hg.1	− 1.353651	0.000651	Down
TC05002688.hg.1	− 1.312714	0.000654	Down
TC19000423.hg.1	− 1.330548	0.000656	Down
TC10002884.hg.1	1.509065	0.000659	Up
TC04002813.hg.1	1.252444	0.000661	Up
TC18000381.hg.1	− 1.501444	0.000664	Down
TC01004281.hg.1	− 1.351776	0.000667	Down
TC11002492.hg.1	− 1.294008	0.000669	Down
TC21000149.hg.1	− 1.321876	0.000672	Down
TC04002187.hg.1	1.496304	0.000677	Up
TC09002847.hg.1	− 1.577697	0.00068	Down
TC09000642.hg.1	− 1.528996	0.000682	Down
TC12001447.hg.1	− 1.218994	0.000685	Down
TC01004750.hg.1	− 1.405462	0.000687	Down
TC01006068.hg.1	− 1.764285	0.000693	Down
TC15001808.hg.1	1.40097	0.000698	Up
TC01002633.hg.1	− 1.562471	7.00E−04	Down
TC19002149.hg.1	1.387591	0.000703	Up
TC02002581.hg.1	− 1.319171	0.000706	Down
TC15002417.hg.1	− 1.354206	0.000708	Down
TC18000603.hg.1	1.83788	0.000711	Up
TC02003574.hg.1	− 1.264549	0.000713	Down
TC09000458.hg.1	− 1.457816	0.000716	Down
TC02003914.hg.1	− 1.539912	0.000721	Down
TC08000285.hg.1	− 1.530341	0.000724	Down
TC01005268.hg.1	1.791888	0.000726	Up
TC02001366.hg.1	− 1.401387	0.000729	Down
TC04001384.hg.1	1.371085	0.000731	Up
TC15001301.hg.1	1.410236	0.000734	Up
TC16002013.hg.1	− 1.607586	0.000739	Down
TC09000907.hg.1	− 1.304972	0.000742	Down
TC21000483.hg.1	− 1.526928	0.000744	Down
TC16001359.hg.1	1.255541	0.00075	Up
TC02002970.hg.1	− 1.479739	0.000755	Down
TC07000690.hg.1	− 1.482383	0.000757	Down
TC12001768.hg.1	− 1.570223	0.00076	Down
TC01005811.hg.1	1.326118	0.000763	Up
TC03001366.hg.1	− 1.272926	0.000765	Down
TC14000809.hg.1	− 1.304326	0.000768	Down
TC10001432.hg.1	− 1.399969	0.000773	Down
TC08000954.hg.1	− 1.3347	0.000778	Down
TC07002238.hg.1	1.338154	0.000781	Up
TC17001691.hg.1	− 1.285264	0.000783	Down
TC03000629.hg.1	− 1.661071	0.000786	Down
TC01003152.hg.1	1.470629	0.000789	Up
TC05001849.hg.1	− 1.23812	0.000791	Down
TC0X002282.hg.1	− 1.368206	0.000794	Down
TC07001801.hg.1	1.645383	0.000796	Up
TC16000919.hg.1	− 1.28532	0.000799	Down
TC16001996.hg.1	− 1.42464	0.000802	Down
TC15000819.hg.1	− 1.492594	0.000804	Down
TC07001099.hg.1	− 1.282727	0.000807	Down
TC01005416.hg.1	1.335933	0.000809	Up
TC08001082.hg.1	− 1.327711	0.000812	Down
TC09002810.hg.1	− 1.337917	0.000815	Down
TC15000346.hg.1	1.616409	0.00082	Up
TC0X000483.hg.1	1.311803	0.000822	Up
TC0X001733.hg.1	1.453339	0.000825	Up
TC01001142.hg.1	1.560006	0.000828	Up
TC12001702.hg.1	− 1.329841	0.00083	Down
TC01002339.hg.1	− 1.416764	0.000833	Down
TC12001455.hg.1	− 1.47722	0.000835	Down
TC02004623.hg.1	− 1.459571	0.000841	Down
TC08002093.hg.1	− 1.281485	0.000843	Down
TC01003671.hg.1	− 1.388896	0.000846	Down
TC09002037.hg.1	− 1.275106	0.000848	Down
TC15002699.hg.1	1.23712	0.000851	Up
TC09000060.hg.1	− 1.327162	0.000854	Down
TC08000411.hg.1	− 1.220223	0.000859	Down
TC03002290.hg.1	− 1.255338	0.000861	Down
TC02001844.hg.1	1.372199	0.000869	Up
TC15002646.hg.1	1.313486	0.000874	Up
TC12000278.hg.1	− 1.226492	0.000877	Down
TC14001143.hg.1	− 1.298709	0.000882	Down
TC04000543.hg.1	1.515491	0.000885	Up
TC04000223.hg.1	− 1.409422	0.000887	Down
TC20001277.hg.1	1.302102	0.00089	Up
TC0X000283.hg.1	− 1.437052	0.000893	Down
TC17001310.hg.1	− 1.246777	0.000895	Down
TC05002436.hg.1	− 1.405807	9.00E−04	Down
TC21000762.hg.1	1.418955	0.000906	Up
TC01002467.hg.1	− 1.36755	0.000908	Down
TC19002431.hg.1	− 1.272804	0.000913	Down
TC08000259.hg.1	− 1.366957	0.000916	Down
TC04002100.hg.1	− 1.333622	0.000932	Down
TC08000621.hg.1	− 1.330254	0.000937	Down
TC07002774.hg.1	− 1.220957	0.000942	Down
TC11000031.hg.1	− 1.301394	0.000945	Down
TC05001757.hg.1	− 1.61845	0.000947	Down
TC15002177.hg.1	1.216648	0.00095	Up
TC01004614.hg.1	− 1.232646	0.000955	Down
TC11003389.hg.1	− 1.289742	0.000958	Down
TC02002711.hg.1	− 1.307176	0.00096	Down
TC02003138.hg.1	1.406275	0.000963	Up
TC07000147.hg.1	− 1.360339	0.000965	Down
TC21000690.hg.1	− 1.263198	0.000968	Down
TC03003246.hg.1	1.264019	0.000971	Up
TC06002340.hg.1	− 1.442259	0.000976	Down
TC07000879.hg.1	1.459915	0.000978	Up
TC02002957.hg.1	− 1.235958	0.000991	Down
TC09002317.hg.1	− 1.249176	0.000994	Down
TC01004251.hg.1	1.286817	0.000999	Up
TC10000250.hg.1	− 1.295565	0.001002	Down
TC12000578.hg.1	1.229407	0.00101	Up
TC03003258.hg.1	1.233627	0.001012	Up
TC06004031.hg.1	− 1.414967	0.001015	Down
TC06001862.hg.1	− 1.33761	0.001017	Down
TC05001595.hg.1	− 1.371588	0.001023	Down
TC11000282.hg.1	− 1.382875	0.001028	Down
TC08001583.hg.1	− 1.478615	0.00103	Down
TC01004440.hg.1	− 1.297319	0.001033	Down
TC12000238.hg.1	− 1.399591	0.001036	Down
TC07000119.hg.1	− 1.27944	0.001043	Down
TC01006051.hg.1	− 1.256434	0.001046	Down
TC02004706.hg.1	1.310009	0.001056	Up
TC02001302.hg.1	− 1.505454	0.001059	Down
TC07000115.hg.1	− 1.22225	0.001062	Down
TC03002003.hg.1	− 1.334984	0.001067	Down
TC17000637.hg.1	1.465593	0.001072	Up
TC01002594.hg.1	− 1.510462	0.001075	Down
TC12000589.hg.1	− 1.365986	0.001077	Down
TC22001135.hg.1	− 1.234824	0.00108	Down
TC09002305.hg.1	1.258887	0.001082	Up
TC17002700.hg.1	− 1.309071	0.001085	Down
TC10002643.hg.1	1.328739	0.001095	Up
TC01000612.hg.1	− 1.316806	0.001098	Down
TC09002594.hg.1	1.380711	0.0011	Up
TC0Y000302.hg.1	− 1.259031	0.001107	Down
TC0Y000304.hg.1	− 1.259031	0.001107	Down
TC04000571.hg.1	− 1.451125	0.001113	Down
TC02002390.hg.1	− 1.333276	0.001116	Down
TC18000785.hg.1	− 1.282574	0.001119	Down
TC07002321.hg.1	1.255896	0.001121	Up
TC09001461.hg.1	− 1.450346	0.001126	Down
TC07002353.hg.1	− 1.289742	0.001129	Down
TC08000734.hg.1	− 1.225764	0.001132	Down
TC07001869.hg.1	− 1.64831	0.001137	Down
TC03000531.hg.1	1.33812	0.001142	Up
TC01002397.hg.1	− 1.297705	0.001145	Down
TC0Y000124.hg.1	1.235109	0.001147	Up
TC09001504.hg.1	− 1.327742	0.00115	Down
TC07000516.hg.1	1.31773	0.001152	Up
TC22001201.hg.1	− 2.532162	0.00116	Down
TC19000785.hg.1	− 1.597258	0.001163	Down
TC08001887.hg.1	1.247089	0.001165	Up
TC10002136.hg.1	− 1.2106	0.001171	Down
TC02001567.hg.1	− 1.384195	0.001176	Down
TC09002851.hg.1	− 1.340312	0.001178	Down
TC21000511.hg.1	1.251439	0.001181	Up
TC20000751.hg.1	− 1.320938	0.001184	Down
TC21000719.hg.1	− 1.268148	0.001189	Down
TC04000456.hg.1	− 1.320603	0.001191	Down
TC15001493.hg.1	− 1.227503	0.001199	Down
TC12002442.hg.1	− 1.376817	0.001207	Down
TC03002939.hg.1	1.251726	0.001212	Up
TC03001734.hg.1	1.396213	0.001215	Up
TC12000588.hg.1	− 1.333007	0.001223	Down
TC21000447.hg.1	− 1.353397	0.001225	Down
TC07000702.hg.1	− 1.47349	0.001228	Down
TC20000413.hg.1	− 1.2025	0.001233	Down
TC18000406.hg.1	1.310188	0.001236	Up
TC04000366.hg.1	1.460437	0.001241	Up
TC03002319.hg.1	− 1.211798	0.001246	Down
TC07000882.hg.1	− 1.465665	0.001256	Down
TC11001732.hg.1	− 1.430354	0.001262	Down
TC11003052.hg.1	1.216234	0.001264	Up
TC06000255.hg.1	1.246413	0.001267	Up
TC02003743.hg.1	1.3116	0.001272	Up
TC0M000020.hg.1	− 1.462929	0.001275	Down
TC03002654.hg.1	1.460058	0.001282	Up
TC20001679.hg.1	1.583808	0.001285	Up
TC01001972.hg.1	1.328821	0.001288	Up
TC02000005.hg.1	− 1.220133	0.00129	Down
TC17001438.hg.1	− 1.37931	0.001298	Down
TC07001428.hg.1	− 1.277175	0.001301	Down
TC11000655.hg.1	− 1.292662	0.001308	Down
TC09002024.hg.1	1.331597	0.001319	Up
TC01003906.hg.1	− 1.246181	0.001324	Down
TC03001758.hg.1	− 1.335816	0.001327	Down
TC11003065.hg.1	− 1.28471	0.001329	Down
TC22001002.hg.1	− 1.300797	0.001337	Down
TC20000993.hg.1	1.406397	0.001342	Up
TC02003376.hg.1	1.22424	0.001345	Up
TC17001702.hg.1	− 1.355539	0.001347	Down
TC0X001193.hg.1	1.492178	0.001358	Up
TC02002904.hg.1	− 1.387512	0.00136	Down
TC02001431.hg.1	− 1.260897	0.001363	Down
TC05000214.hg.1	− 1.320326	0.001366	Down
TC11003186.hg.1	1.239331	0.001371	Up
TC12003071.hg.1	− 1.273357	0.001379	Down
TC11002465.hg.1	− 1.301587	0.001381	Down
TC02001721.hg.1	− 1.243881	0.001384	Down
TC03001672.hg.1	− 1.312188	0.001386	Down
TC04001562.hg.1	1.448978	0.001397	Up
TC11002930.hg.1	1.32439	0.001399	Up
TC01005621.hg.1	− 1.346925	0.001402	Down
TC14000912.hg.1	− 1.274825	0.001405	Down
TC12000990.hg.1	− 1.323786	0.001415	Down
TC01003976.hg.1	1.256443	0.001418	Up
TC16001253.hg.1	− 1.403981	0.001425	Down
TC20001202.hg.1	1.266515	0.001436	Up
TC16000168.hg.1	− 1.27072	0.001438	Down
TC09002408.hg.1	1.520922	0.001441	Up
TC16000573.hg.1	− 1.259628	0.001451	Down
TC15000227.hg.1	− 1.252319	0.001454	Down
TC10000082.hg.1	− 1.386938	0.001456	Down
TC04000854.hg.1	− 1.36653	0.001459	Down
TC09002413.hg.1	− 1.24335	0.001462	Down
TC17002577.hg.1	− 1.227651	0.001464	Down
TC0M000023.hg.1	− 1.325689	0.001467	Down
TC0X000584.hg.1	1.524739	0.001482	Up
TC17000782.hg.1	− 1.248164	0.001485	Down
TC01000565.hg.1	− 1.252749	0.00149	Down
TC06002722.hg.1	− 1.238586	0.001495	Down
TC05001279.hg.1	− 1.228112	0.001498	Down
TC05002924.hg.1	− 1.252361	0.001501	Down
TC01000711.hg.1	− 1.284644	0.001516	Down
TC02000092.hg.1	− 1.239683	0.001534	Down
TC07000209.hg.1	− 1.234204	0.001537	Down
TC07001848.hg.1	− 1.342223	0.001547	Down
TC13001179.hg.1	− 1.291746	0.00155	Down
TC15002627.hg.1	− 1.241372	0.001553	Down
TC03000245.hg.1	− 1.251682	0.001555	Down
TC11002051.hg.1	− 1.298163	0.001558	Down
TC01005264.hg.1	− 1.432014	0.001568	Down
TC13000269.hg.1	− 1.405203	0.001571	Down
TC05001668.hg.1	− 1.264735	0.001573	Down
TC02000188.hg.1	− 1.278172	0.001592	Down
TC20000564.hg.1	− 1.288599	0.001594	Down
TC02002994.hg.1	− 1.487671	0.001597	Down
TC07002487.hg.1	− 1.275774	0.001607	Down
TC17001587.hg.1	− 1.227915	0.00161	Down
TC14000711.hg.1	− 1.272822	0.00162	Down
TC02003983.hg.1	− 1.242	0.001628	Down
TC05003265.hg.1	− 1.230554	0.001636	Down
TC14000850.hg.1	− 1.216954	0.001649	Down
TC08000062.hg.1	− 1.269982	0.001654	Down
TC02004175.hg.1	− 1.224219	0.001675	Down
TC06001872.hg.1	− 1.257336	0.00168	Down
TC08000176.hg.1	− 1.220994	0.001693	Down
TC15001480.hg.1	− 1.381133	0.001701	Down
TC03000233.hg.1	− 1.272324	0.001722	Down
TC09002445.hg.1	− 1.336146	0.001745	Down
TC08001621.hg.1	− 1.261265	0.001758	Down
TC09001799.hg.1	− 1.266496	0.001763	Down
TC12001128.hg.1	− 1.30415	0.001766	Down
TC01001028.hg.1	− 1.345191	0.001787	Down
TC05000708.hg.1	− 1.476725	0.001797	Down
TC04000307.hg.1	− 1.201551	0.001802	Down
TC04001876.hg.1	− 1.312985	0.001833	Down
TC02004514.hg.1	− 1.297988	0.001838	Down
TC12003114.hg.1	− 1.324803	0.001846	Down
TC12000184.hg.1	− 1.231818	0.001849	Down
TC12002387.hg.1	− 1.258686	0.001859	Down
TC0X000282.hg.1	− 1.45897	0.001862	Down
TC17001193.hg.1	− 1.316251	0.001867	Down
TC03001348.hg.1	− 1.255899	0.00187	Down
TC11002740.hg.1	− 1.257394	0.001872	Down
TC16000982.hg.1	− 1.234762	0.001875	Down
TC01004296.hg.1	− 1.287062	0.001877	Down
TC17002761.hg.1	− 1.320442	0.001883	Down
TC03000215.hg.1	− 1.209242	0.001885	Down
TC21000941.hg.1	− 1.285527	0.00189	Down
TC01005631.hg.1	− 1.230716	0.001903	Down
TC05002180.hg.1	− 1.262406	0.00194	Down
TC02001854.hg.1	− 1.397183	0.001945	Down
TC02001762.hg.1	− 1.306576	0.001953	Down
TC20001199.hg.1	− 1.647289	0.001963	Down
TC09001709.hg.1	− 1.554081	0.001968	Down
TC09001939.hg.1	− 1.294994	0.001987	Down
TC0X000279.hg.1	− 1.345356	0.001989	Down
TC0X000632.hg.1	− 1.266722	0.002023	Down
TC09000488.hg.1	− 1.235635	0.002028	Down
TC16001481.hg.1	− 1.280691	0.002031	Down
TC14000004.hg.1	− 1.297753	0.002036	Down
TC03000982.hg.1	− 1.296044	0.002044	Down
TC17001757.hg.1	− 1.218957	0.002049	Down
TC09002325.hg.1	− 1.326214	0.002062	Down
TC06000943.hg.1	− 1.305433	0.002067	Down
TC01003971.hg.1	− 1.348647	0.002091	Down
TC6_cox_hap2000100.hg.1	− 1.554297	0.002098	Down
TC15001904.hg.1	− 1.252785	0.002106	Down
TC01002757.hg.1	− 1.351323	0.002109	Down
TC09001332.hg.1	− 1.443525	0.002111	Down
TC10002850.hg.1	− 1.234143	0.002124	Down
TC04000005.hg.1	− 1.301943	0.002127	Down
TC01005454.hg.1	− 1.236656	0.00213	Down
TC06000102.hg.1	− 1.270327	0.002137	Down
TC10000369.hg.1	− 1.297064	0.00214	Down
TC05001222.hg.1	− 1.318072	0.002153	Down
TC14002139.hg.1	− 1.315965	0.002192	Down
TC12001777.hg.1	− 1.361163	0.002194	Down
TC0Y000257.hg.1	− 1.300159	0.0022	Down
TC14000798.hg.1	− 1.51072	0.002202	Down
TC10001326.hg.1	− 1.235462	0.002213	Down
TC08001540.hg.1	− 1.313364	0.00222	Down
TC18000718.hg.1	− 1.313197	0.002226	Down
TC17002085.hg.1	− 1.303126	0.002249	Down
TC20001091.hg.1	− 1.214845	0.002262	Down
TC15001671.hg.1	− 1.222416	0.00227	Down
TC01003519.hg.1	− 1.227491	0.00228	Down
TC19001173.hg.1	− 1.504936	0.002293	Down
TC06003422.hg.1	− 1.26218	0.002306	Down
TC14001264.hg.1	− 1.271609	0.002314	Down
TC0X000986.hg.1	− 1.615057	0.002324	Down
TC10002901.hg.1	− 1.237562	0.00233	Down
TC11001264.hg.1	− 1.288498	0.002337	Down
TC14000795.hg.1	− 1.294836	0.002356	Down
TC05002063.hg.1	− 1.291118	0.002374	Down
TC11002064.hg.1	− 1.253136	0.002382	Down
TC17001939.hg.1	− 1.311247	0.002384	Down
TC02000413.hg.1	− 1.221091	0.002395	Down
TC08000956.hg.1	− 1.294681	0.002423	Down
TC05000526.hg.1	− 1.202821	0.002426	Down
TC02004253.hg.1	− 1.236233	0.002428	Down
TC01004790.hg.1	− 1.213559	0.002431	Down
TC02003570.hg.1	− 1.266677	0.002439	Down
TC04000311.hg.1	− 1.231832	0.002444	Down
TC19001288.hg.1	− 1.25999	0.002454	Down
TC14000697.hg.1	− 1.404857	0.002483	Down
TC09001077.hg.1	− 1.253924	0.002496	Down
TC21000650.hg.1	− 1.255534	0.002501	Down
TC07000862.hg.1	− 1.26199	0.002504	Down
TC09001024.hg.1	− 1.234413	0.002509	Down
TC03000889.hg.1	− 1.455333	0.002517	Down
TC04002712.hg.1	− 1.205148	0.002522	Down
TC11000172.hg.1	− 1.3994	0.00253	Down
TC22001095.hg.1	− 1.245068	0.002532	Down
TC17000164.hg.1	− 1.350111	0.002556	Down
TC02003220.hg.1	− 1.27533	0.002561	Down
TC02003240.hg.1	− 1.27617	0.002576	Down
TC01002756.hg.1	− 1.451337	0.002592	Down
TC04000671.hg.1	− 1.288484	0.002597	Down
TC11003125.hg.1	− 1.237544	0.002628	Down
TC08000224.hg.1	− 1.240388	0.002647	Down
TC15002487.hg.1	− 1.375221	0.002649	Down
TC02004203.hg.1	− 1.328396	0.00267	Down
TC07002457.hg.1	− 1.2626	0.002699	Down
TC11000768.hg.1	− 1.278624	0.002704	Down
TC01001663.hg.1	− 1.240612	0.002719	Down
TC01000364.hg.1	− 1.295795	0.002722	Down
TC14001177.hg.1	− 1.215351	0.00273	Down
TC18000510.hg.1	− 1.339711	0.002743	Down
TC15001577.hg.1	− 1.296726	0.002748	Down
TC11002224.hg.1	− 1.418547	0.002761	Down
TC05002475.hg.1	− 1.29094	0.002769	Down
TC04001202.hg.1	− 1.267133	0.002777	Down
TC15001164.hg.1	− 1.256707	0.002784	Down
TC06002310.hg.1	− 1.205867	0.00281	Down
TC12000236.hg.1	− 1.234045	0.002813	Down
TC14000661.hg.1	− 1.277726	0.002826	Down
TC15002579.hg.1	− 1.217318	0.002849	Down
TC09000592.hg.1	− 1.249078	0.002857	Down
TC19000842.hg.1	− 1.275512	0.002873	Down
TC01001511.hg.1	− 1.274328	0.002878	Down
TC11000297.hg.1	− 1.333831	0.002883	Down
TC01005988.hg.1	− 1.399566	0.002899	Down
TC06000972.hg.1	− 1.307552	0.002912	Down
TC12000683.hg.1	− 1.31692	0.002919	Down
TC03000359.hg.1	− 1.2305	0.002938	Down
TC01006043.hg.1	− 1.294354	0.002956	Down
TC06001203.hg.1	− 1.210835	0.002977	Down
TC09002250.hg.1	− 1.30785	0.002979	Down
TC06003965.hg.1	− 1.25692	0.002982	Down
TC06001689.hg.1	− 1.271407	0.002995	Down
TC19000016.hg.1	− 1.280763	0.003005	Down
TC02000420.hg.1	− 1.290683	0.003016	Down
TC12001821.hg.1	− 1.233869	0.003031	Down
TC0X001706.hg.1	− 1.201138	0.003044	Down
TC02000115.hg.1	− 1.210348	0.003057	Down
TC02001877.hg.1	− 1.334891	0.00306	Down
TC21000155.hg.1	− 1.319149	0.00307	Down
TC09002217.hg.1	− 1.202281	0.003075	Down
TC14000698.hg.1	− 1.271845	0.003078	Down
TC12002927.hg.1	− 1.241343	0.003083	Down
TC15002068.hg.1	− 1.295466	0.003091	Down
TC03000673.hg.1	− 1.219704	0.003117	Down
TC21000774.hg.1	− 1.304279	0.003122	Down
TC06002185.hg.1	− 1.21637	0.003138	Down
TC09000159.hg.1	− 1.452667	0.003143	Down
TC08002174.hg.1	− 1.299286	0.003174	Down
TC13000052.hg.1	− 1.220349	0.003226	Down
TC03002972.hg.1	− 1.308676	0.003244	Down
TC06003115.hg.1	− 1.208136	0.003278	Down
TC20000973.hg.1	− 1.239161	0.003296	Down
TC02004167.hg.1	− 1.20315	0.003299	Down
TC01005639.hg.1	− 1.239478	0.003304	Down
TC03003205.hg.1	− 1.263929	0.003317	Down
TC03002458.hg.1	− 1.287128	0.003325	Down
TC21000403.hg.1	− 1.222955	0.003335	Down
TC05002143.hg.1	− 1.436058	0.003346	Down
TC10000872.hg.1	− 1.210735	0.003348	Down
TC02003109.hg.1	− 1.228425	0.003361	Down
TC14000289.hg.1	− 1.307652	0.003374	Down
TC01002426.hg.1	− 1.214	0.003392	Down
TC11002900.hg.1	− 1.365245	0.0034	Down
TC04000600.hg.1	− 1.241726	0.003426	Down
TC02003391.hg.1	− 1.29186	0.003429	Down
TC01003999.hg.1	− 1.489871	0.003439	Down
TC04001552.hg.1	− 1.2639	0.003444	Down
TC12001091.hg.1	− 1.253306	0.003463	Down
TC06000930.hg.1	− 1.300405	0.003502	Down
TC08000464.hg.1	− 1.271156	0.003504	Down
TC05003306.hg.1	− 1.211566	0.003515	Down
TC16001224.hg.1	− 1.334827	0.003546	Down
TC11002505.hg.1	− 1.368315	0.003551	Down
TC03000929.hg.1	− 1.21097	0.003567	Down
TC15001656.hg.1	− 1.256362	0.003592	Down
TC14000427.hg.1	− 1.252462	0.003605	Down
TC06003126.hg.1	− 1.282658	0.003613	Down
TC08001731.hg.1	− 1.210525	0.003616	Down
TC0X000016.hg.1	− 1.222038	0.003618	Down
TC10001940.hg.1	− 1.277388	0.003624	Down
TC11000653.hg.1	− 1.214434	0.003626	Down
TC01001760.hg.1	− 1.317357	0.003631	Down
TC0X000285.hg.1	− 1.237755	0.003634	Down
TC05002272.hg.1	− 1.23028	0.003644	Down
TC13000706.hg.1	− 1.295325	0.003652	Down
TC07002574.hg.1	− 1.288074	0.003655	Down
TC05001287.hg.1	− 1.257783	0.003657	Down
TC02002347.hg.1	− 1.216468	0.003681	Down
TC19000786.hg.1	− 1.238899	0.00372	Down
TC22001164.hg.1	− 1.222475	0.003733	Down
TC03000249.hg.1	− 1.322144	0.003782	Down
TC02001846.hg.1	− 1.290844	0.003798	Down
TC13000062.hg.1	− 1.293684	0.003819	Down
TC11000065.hg.1	− 1.20357	0.003855	Down
TC01005521.hg.1	− 1.290967	0.003878	Down
TC19002432.hg.1	− 1.300036	0.003881	Down
TC01005957.hg.1	− 1.320058	0.003894	Down
TC03000122.hg.1	− 1.236915	0.003915	Down
TC17001999.hg.1	− 1.214198	0.003925	Down
TC12002870.hg.1	− 1.233811	0.003928	Down
TC01002874.hg.1	− 1.282038	0.003933	Down
TC01005965.hg.1	− 1.205432	0.003951	Down
TC09002644.hg.1	− 1.242856	0.003954	Down
TC05000653.hg.1	− 1.236994	0.003956	Down
TC11003292.hg.1	− 1.214328	0.003964	Down
TC17002315.hg.1	− 1.22301	0.00398	Down
TC17000557.hg.1	− 1.378115	0.00399	Down
TC11002959.hg.1	− 1.276354	0.003993	Down
TC19000244.hg.1	− 1.274288	0.003995	Down
TC20000441.hg.1	− 1.2081	0.004026	Down
TC16001268.hg.1	− 1.246905	0.004029	Down
TC13000651.hg.1	− 1.304621	0.004042	Down
TC07003181.hg.1	− 1.236251	0.004045	Down
TC01004593.hg.1	− 1.200072	0.004047	Down
TC19002136.hg.1	− 1.202992	0.004058	Down
TC10000755.hg.1	− 1.320862	0.00406	Down
TC02001259.hg.1	− 1.269599	0.004097	Down
TC07002494.hg.1	− 1.221869	0.004115	Down
TC17001828.hg.1	− 1.355253	0.004128	Down
TC12002637.hg.1	− 1.268176	0.004133	Down
TC03002947.hg.1	− 1.235059	0.004146	Down
TC03003240.hg.1	− 1.243183	0.004149	Down
TC10001764.hg.1	− 1.262831	0.004211	Down
TC11001522.hg.1	− 1.31939	0.004253	Down
TC05002576.hg.1	− 1.280722	0.004273	Down
TC11002966.hg.1	− 1.233463	0.004304	Down
TC0Y000049.hg.1	− 1.326577	0.004307	Down
TC14000660.hg.1	− 1.203246	0.00431	Down
TC07001989.hg.1	− 1.221607	0.004315	Down
TC08001194.hg.1	− 1.343792	0.004317	Down
TC18000216.hg.1	− 1.249015	0.004346	Down
TC14000282.hg.1	− 1.245247	0.004351	Down
TC07003162.hg.1	− 1.203476	0.004375	Down
TC08002317.hg.1	− 1.278531	0.004377	Down
TC09001614.hg.1	− 1.347077	0.00438	Down
TC10002616.hg.1	− 1.303518	0.004388	Down
TC11002989.hg.1	− 1.281997	0.00439	Down
TC03003014.hg.1	− 1.202168	0.004393	Down
TC06003938.hg.1	− 1.266069	0.004445	Down
TC03002962.hg.1	− 1.251379	0.004481	Down
TC12002295.hg.1	− 1.289958	0.004494	Down
TC6_cox_hap2000194.hg.1	− 1.306576	0.004499	Down
TC15002555.hg.1	− 1.204674	0.004502	Down
TC20000160.hg.1	− 1.231493	0.00452	Down
TC04002803.hg.1	− 1.318666	0.004546	Down
TC04000837.hg.1	− 1.219779	0.00459	Down
TC13001207.hg.1	− 1.212648	0.004609	Down
TC15000190.hg.1	− 1.209617	0.004611	Down
TC02001190.hg.1	− 1.260423	0.004629	Down
TC14000251.hg.1	− 1.23846	0.004632	Down
TC01002804.hg.1	− 1.237783	0.004637	Down
TC16000469.hg.1	− 1.212053	0.004666	Down
TC09000173.hg.1	− 1.216822	0.004715	Down
TC07002912.hg.1	− 1.268587	0.004731	Down
TC19002439.hg.1	− 1.281673	0.004759	Down
TC15002362.hg.1	− 1.249402	0.004772	Down
TC07001701.hg.1	− 1.251725	0.00478	Down
TC06003627.hg.1	− 1.250247	0.004783	Down
TC07002791.hg.1	− 1.204493	0.004796	Down
TC17001730.hg.1	− 1.228444	0.004809	Down
TC03001001.hg.1	− 1.228118	0.004897	Down

Probe set ID	Fold change	p-value	Gene feature
(D) *Genes up/down-regulated in purified TCs*^*SV40*^ *compared with non-purified lung TCs*^*SV40*^			
TC11000967.hg.1	− 3.281369	2.70E−05	Down
TC07000780.hg.1	− 1.970289	3.00E−05	Down
TC11002894.hg.1	− 2.295351	3.30E−05	Down
TC05000152.hg.1	− 1.620079	3.50E−05	Down
TC08002510.hg.1	− 1.933489	3.80E−05	Down
TC03002042.hg.1	− 1.619648	4.00E−05	Down
TC11001141.hg.1	− 1.537863	4.30E−05	Down
TC02000336.hg.1	− 1.625021	4.60E−05	Down
TC11003109.hg.1	− 2.701391	4.80E−05	Down
TC12000188.hg.1	− 1.64374	5.10E−05	Down
TC06002006.hg.1	− 1.597063	5.30E− 05	Down
TC08001415.hg.1	− 1.642419	5.60E−05	Down
TC10000962.hg.1	− 1.45532	5.90E−05	Down
TC10000791.hg.1	− 1.651855	6.10E−05	Down
TC01005437.hg.1	− 1.487989	6.40E−05	Down
TC19000951.hg.1	− 1.582882	6.60E−05	Down
TC17002196.hg.1	1.361752	6.90E−05	Up
TC17002479.hg.1	− 1.373139	7.70E−05	Down
TC02004623.hg.1	1.714167	7.90E−05	Up
TC16001576.hg.1	1.424831	8.50E−05	Up
TC06000041.hg.1	− 1.393334	8.70E−05	Down
TC08002421.hg.1	1.950551	9.00E−05	Up
TC08002059.hg.1	− 1.709135	9.20E−05	Down
TC05001595.hg.1	1.323614	9.50E−05	Up
TC02004746.hg.1	1.393606	1.00E−04	Up
TC15002609.hg.1	1.312481	0.000108	Up
TC16000510.hg.1	− 1.498391	0.000111	Down
TC04002890.hg.1	− 1.463155	0.000113	Down
TC09002783.hg.1	− 1.50221	0.000116	Down
TC14000925.hg.1	− 1.412739	0.000124	Down
TC15000651.hg.1	− 1.510536	0.000129	Down
TC0M000021.hg.1	− 1.703812	0.000131	Down
TC05000861.hg.1	− 1.468789	0.000134	Down
TC09000963.hg.1	− 1.357361	0.000152	Down
TC22000098.hg.1	− 1.431959	0.000163	Down
TC14000667.hg.1	− 1.38192	0.000165	Down
TC03002349.hg.1	− 1.491439	0.00017	Down
TC03003114.hg.1	− 1.600835	0.000183	Down
TC09002305.hg.1	− 1.291981	0.000199	Down
TC17000728.hg.1	− 1.727707	0.000204	Down
TC04002772.hg.1	− 1.366681	0.000207	Down
TC05001371.hg.1	− 1.373092	0.000209	Down
TC0X001822.hg.1	− 1.264641	0.000212	Down
TC02001623.hg.1	− 1.416467	0.000215	Down
TC08001239.hg.1	− 1.365024	0.000222	Down
TC03003246.hg.1	− 1.29006	0.000228	Down
TC20000203.hg.1	− 1.357278	0.000235	Down
TC0M000020.hg.1	− 4.071566	0.000259	Down

Probe set ID	Fold change	p-value	Gene feature
(E) *Genes up/down-regulated in purified TCs*^*SV40*^ *compared with purified primary lung TCs*			
TC11002382.hg.1	− 4.090002	3.30E−05	Down
TC17000728.hg.1	− 3.396128	2.70E−05	Down
TC11000967.hg.1	− 3.004215	9.80E−05	Down
TC08002253.hg.1	− 2.443727	0.000155	Down
TC17001801.hg.1	− 2.309379	9.00E−05	Down
TC07000780.hg.1	− 2.304878	3.80E−05	Down
TC06000639.hg.1	− 2.28677	4.60E−05	Down
TC20000045.hg.1	− 2.220467	0.00034	Down
TC04001945.hg.1	− 2.095604	0.000225	Down
TC12000927.hg.1	− 2.039933	4.80E−05	Down
TC13000783.hg.1	− 2.038291	0.000288	Down
TC02001799.hg.1	− 2.015351	0.002366	Down
TC11002142.hg.1	− 1.996031	0.00051	Down
TC16000648.hg.1	− 1.975845	0.000783	Down
TC06001978.hg.1	− 1.959951	3.00E−05	Down
TC09000963.hg.1	− 1.9478	0.000152	Down
TC0X001770.hg.1	− 1.947499	0.000301	Down
TC09001648.hg.1	− 1.947349	6.60E−05	Down
TC04001635.hg.1	− 1.946272	0.000205	Down
TC02001940.hg.1	− 1.933961	9.50E−05	Down
TC15000452.hg.1	− 1.930945	4.00E−05	Down
TC07000702.hg.1	− 1.910595	8.20E−05	Down
TC0X001158.hg.1	− 1.88932	0.000434	Down
TC08000302.hg.1	− 1.854536	5.10E−05	Down
TC20000952.hg.1	− 1.846904	0.00015	Down
TC0X000985.hg.1	− 1.821657	0.000168	Down
TC07001784.hg.1	− 1.818055	4.30E−05	Down
TC09000971.hg.1	− 1.814467	8.50E−05	Down
TC08001415.hg.1	− 1.813106	6.40E−05	Down
TC09000096.hg.1	− 1.808112	0.000392	Down
TC02000205.hg.1	− 1.798757	0.00016	Down
TC12000188.hg.1	− 1.798296	7.40E−05	Down
TC01003308.hg.1	− 1.786611	0.000171	Down
TC19000767.hg.1	− 1.780988	1.00E−04	Down
TC05000152.hg.1	− 1.780756	0.000121	Down
TC02000336.hg.1	− 1.777235	8.70E−05	Down
TC02002456.hg.1	− 1.775443	8.00E− 05	Down
TC02000396.hg.1	− 1.770308	5.30E−05	Down
TC12000633.hg.1	− 1.765148	7.70E−05	Down
TC05001593.hg.1	− 1.752631	7.20E−05	Down
TC19000951.hg.1	− 1.750403	0.000124	Down
TC09001461.hg.1	− 1.742247	0.000111	Down
TC03002042.hg.1	− 1.741147	0.000103	Down
TC12001286.hg.1	− 1.726658	0.000176	Down
TC06002006.hg.1	− 1.707544	0.000132	Down
TC01006068.hg.1	− 1.692979	0.000134	Down
TC05002800.hg.1	− 1.691325	0.000312	Down
TC16000510.hg.1	− 1.682589	0.00027	Down
TC01002594.hg.1	− 1.674638	0.000486	Down
TC16001289.hg.1	− 1.671928	5.90E−05	Down
TC10000791.hg.1	− 1.658964	0.000272	Down
TC01002663.hg.1	− 1.6563	0.002108	Down
TC12002688.hg.1	− 1.654776	0.000166	Down
TC20000650.hg.1	− 1.641248	0.000126	Down
TC11001010.hg.1	− 1.634925	0.000192	Down
TC02004814.hg.1	− 1.627547	0.000257	Down
TC03000874.hg.1	− 1.624355	0.00168	Down
TC20000373.hg.1	− 1.622438	0.000106	Down
TC0X001624.hg.1	− 1.620312	0.000523	Down
TC22000098.hg.1	− 1.615575	0.000246	Down
TC01002798.hg.1	− 1.61537	0.000262	Down
TC0X001292.hg.1	− 1.607909	0.001472	Down
TC09002651.hg.1	− 1.603388	0.003161	Down
TC05000708.hg.1	− 1.59612	0.000254	Down
TC12000244.hg.1	− 1.589748	0.000817	Down
TC01002746.hg.1	− 1.585136	0.000377	Down
TC0X001411.hg.1	− 1.576617	0.000484	Down
TC01002633.hg.1	− 1.57574	0.000447	Down
TC14000925.hg.1	− 1.5746	0.000332	Down
TC06004042.hg.1	− 1.572429	0.000173	Down
TC03001841.hg.1	− 1.571987	0.000129	Down
TC02001302.hg.1	− 1.571147	0.000223	Down
TC09001534.hg.1	− 1.570612	0.000457	Down
TC20001199.hg.1	− 1.567384	0.000431	Down
TC02001432.hg.1	− 1.56216	0.000306	Down
TC04000508.hg.1	− 1.560484	0.000202	Down
TC06001365.hg.1	− 1.559188	0.000689	Down
TC11002077.hg.1	− 1.558571	0.000489	Down
TC02002994.hg.1	− 1.554748	0.000744	Down
TC15001073.hg.1	− 1.553387	0.000645	Down
TC02001602.hg.1	− 1.549962	0.000218	Down
TC0X001618.hg.1	− 1.549945	0.000405	Down
TC15002586.hg.1	− 1.54835	0.000593	Down
TC22001417.hg.1	− 1.548268	0.000382	Down
TC05000861.hg.1	− 1.545398	0.00045	Down
TC19000163.hg.1	− 1.544743	0.000145	Down
TC06000568.hg.1	− 1.542824	0.000343	Down
TC08000801.hg.1	− 1.540416	0.000137	Down
TC08002335.hg.1	− 1.538863	0.003182	Down
TC11002486.hg.1	− 1.538725	0.000293	Down
TC0X000279.hg.1	− 1.534468	0.002392	Down
TC10000866.hg.1	− 1.528931	0.000252	Down
TC10000962.hg.1	− 1.528853	0.000184	Down
TC15000651.hg.1	− 1.527445	0.0024	Down
TC09001602.hg.1	− 1.527117	0.000285	Down
TC14000052.hg.1	− 1.523268	0.000666	Down
TC05000621.hg.1	− 1.521581	0.000945	Down
TC03000195.hg.1	− 1.520243	0.000239	Down
TC17001773.hg.1	− 1.514647	0.000199	Down
TC02001366.hg.1	− 1.514152	0.00033	Down
TC08001315.hg.1	− 1.513587	0.000669	Down
TC11002476.hg.1	− 1.512413	0.000549	Down
TC15001473.hg.1	− 1.511023	0.000439	Down
TC07001655.hg.1	− 1.508092	0.000624	Down
TC09000100.hg.1	− 1.506552	0.001224	Down
TC05002818.hg.1	− 1.505908	0.000374	Down
TC11001141.hg.1	− 1.505044	0.000882	Down
TC0X001410.hg.1	− 1.504643	0.000497	Down
TC22001175.hg.1	− 1.504265	0.000418	Down
TC08000450.hg.1	− 1.497675	0.000442	Down
TC17000491.hg.1	− 1.494822	0.000478	Down
TC19001173.hg.1	− 1.48853	0.003494	Down
TC15001326.hg.1	− 1.486591	0.000113	Down
TC16002013.hg.1	− 1.486347	0.000228	Down
TC15000631.hg.1	− 1.485978	0.000356	Down
TC12001052.hg.1	− 1.485625	0.001544	Down
TC16000853.hg.1	− 1.48534	0.001049	Down
TC01002976.hg.1	− 1.483998	0.000244	Down
TC12001449.hg.1	− 1.481302	0.000358	Down
TC12001995.hg.1	− 1.476015	0.000504	Down
TC0X001136.hg.1	− 1.473457	0.000265	Down
TC06001064.hg.1	− 1.473031	0.001156	Down
TC09000516.hg.1	− 1.466985	0.001977	Down
TC09001936.hg.1	− 1.461819	0.001954	Down
TC13001206.hg.1	− 1.461709	0.002804	Down
TC14000620.hg.1	− 1.461092	0.000197	Down
TC10002439.hg.1	− 1.460412	0.001052	Down
TC07000139.hg.1	− 1.459424	0.000979	Down
TC04002443.hg.1	− 1.457362	0.00063	Down
TC0X000450.hg.1	− 1.456236	0.00052	Down
TC03001571.hg.1	− 1.454824	0.000327	Down
TC06002801.hg.1	− 1.452915	0.000283	Down
TC10002919.hg.1	− 1.452582	0.000259	Down
TC02000955.hg.1	− 1.450519	0.001299	Down
TC05002279.hg.1	− 1.449507	0.000416	Down
TC09002783.hg.1	− 1.445945	0.000452	Down
TC01003510.hg.1	− 1.445715	0.000364	Down
TC0X001762.hg.1	− 1.445315	0.000554	Down
TC22000400.hg.1	− 1.445268	0.000296	Down
TC02001623.hg.1	− 1.444939	0.00089	Down
TC07001706.hg.1	− 1.442104	0.001574	Down
TC07001709.hg.1	− 1.442104	0.001574	Down
TC08000652.hg.1	− 1.441877	0.001378	Down
TC15001604.hg.1	− 1.440453	0.000463	Down
TC04001524.hg.1	− 1.437658	0.000158	Down
TC14001281.hg.1	− 1.433784	0.000471	Down
TC02002768.hg.1	− 1.428308	0.00059	Down
TC05002975.hg.1	− 1.427776	0.000421	Down
TC10001432.hg.1	− 1.427505	7.00E−04	Down
TC06000041.hg.1	− 1.427026	0.000319	Down
TC06000705.hg.1	− 1.426715	0.000984	Down
TC01002620.hg.1	− 1.426413	0.00039	Down
TC06003710.hg.1	− 1.420694	0.003033	Down
TC07000339.hg.1	− 1.419104	0.00094	Down
TC05000526.hg.1	− 1.417126	0.000345	Down
TC02001836.hg.1	− 1.417083	0.000971	Down
TC01003990.hg.1	− 1.4165	0.001091	Down
TC10002673.hg.1	− 1.416168	0.001312	Down
TC21000483.hg.1	− 1.415996	0.002217	Down
TC02003914.hg.1	− 1.413985	0.000627	Down
TC07001267.hg.1	− 1.412486	0.000437	Down
TC10002723.hg.1	− 1.411693	0.002092	Down
TC03000155.hg.1	− 1.410721	0.001591	Down
TC10000793.hg.1	− 1.409945	0.001221	Down
TC03001056.hg.1	− 1.408329	0.000278	Down
TC10000686.hg.1	− 1.407419	0.000215	Down
TC14001892.hg.1	− 1.406093	0.000366	Down
TC21000698.hg.1	− 1.405176	0.0012	Down
TC06001651.hg.1	− 1.404117	0.000139	Down
TC22000272.hg.1	− 1.402122	0.002227	Down
TC14002171.hg.1	− 1.401997	0.000528	Down
TC06002303.hg.1	− 1.401899	0.001466	Down
TC10001189.hg.1	− 1.400004	0.000611	Down
TC18000381.hg.1	− 1.398874	0.001571	Down
TC01002339.hg.1	− 1.398183	0.000408	Down
TC14001090.hg.1	− 1.396639	0.001443	Down
TC02002028.hg.1	− 1.39541	0.001099	Down
TC07001289.hg.1	− 1.395341	0.001604	Down
TC07000690.hg.1	− 1.394396	0.000317	Down
TC11001479.hg.1	− 1.394041	0.001657	Down
TC12003094.hg.1	− 1.391962	0.001722	Down
TC03000823.hg.1	− 1.390073	0.000619	Down
TC05002924.hg.1	− 1.388742	0.001779	Down
TC05000703.hg.1	− 1.388715	0.00369	Down
TC12000231.hg.1	− 1.388427	0.000186	Down
TC03002201.hg.1	− 1.38807	0.001104	Down
TC05000688.hg.1	− 1.387472	0.000776	Down
TC09001410.hg.1	− 1.385276	0.002814	Down
TC01003999.hg.1	− 1.384791	0.001714	Down
TC03000962.hg.1	− 1.384037	0.002342	Down
TC10001912.hg.1	− 1.381688	0.0015	Down
TC01002393.hg.1	− 1.381656	0.00193	Down
TC05000214.hg.1	− 1.378498	0.001206	Down
TC02001248.hg.1	− 1.377049	0.000515	Down
TC0X002258.hg.1	− 1.375302	0.000833	Down
TC16002030.hg.1	− 1.374782	0.000395	Down
TC03000629.hg.1	− 1.37476	0.001255	Down
TC07001657.hg.1	− 1.373948	0.001018	Down
TC0X000283.hg.1	− 1.373835	0.003038	Down
TC10000755.hg.1	− 1.372467	0.003283	Down
TC06000824.hg.1	− 1.371586	0.002728	Down
TC11000089.hg.1	− 1.371419	0.000371	Down
TC18000050.hg.1	− 1.370569	0.001226	Down
TC16001555.hg.1	− 1.369024	0.000387	Down
TC20000340.hg.1	− 1.368516	0.00363	Down
TC01005517.hg.1	− 1.368066	0.002598	Down
TC0X000232.hg.1	− 1.36715	0.001594	Down
TC01004166.hg.1	− 1.365917	0.000976	Down
TC08001640.hg.1	− 1.365564	0.002254	Down
TC05000183.hg.1	− 1.365261	0.001818	Down
TC05000876.hg.1	− 1.364983	0.000961	Down
TC10000439.hg.1	− 1.364697	0.000298	Down
TC09002445.hg.1	− 1.364017	0.002141	Down
TC03002972.hg.1	− 1.36302	0.001763	Down
TC09001709.hg.1	− 1.362426	0.00119	Down
TC09002851.hg.1	− 1.36068	0.000577	Down
TC21000931.hg.1	− 1.360378	0.000596	Down
TC05002802.hg.1	− 1.360064	0.003648	Down
TC05002034.hg.1	− 1.358914	0.001698	Down
TC11000768.hg.1	− 1.358104	0.001164	Down
TC11000808.hg.1	− 1.357692	4.00E−04	Down
TC05002959.hg.1	− 1.356675	0.000739	Down
TC16001991.hg.1	− 1.355237	0.000426	Down
TC03001321.hg.1	− 1.352844	0.000476	Down
TC17000230.hg.1	− 1.352713	0.000935	Down
TC05001562.hg.1	− 1.352471	0.002853	Down
TC06002451.hg.1	− 1.351413	0.003281	Down
TC14000693.hg.1	− 1.351022	0.001693	Down
TC06001125.hg.1	− 1.350964	0.003685	Down
TC12000805.hg.1	− 1.349635	0.001664	Down
TC10000815.hg.1	− 1.349597	0.000682	Down
TC08000752.hg.1	− 1.348369	0.003401	Down
TC01005988.hg.1	− 1.347966	0.001354	Down
TC16001082.hg.1	− 1.347545	0.001967	Down
TC06000972.hg.1	− 1.347335	0.001701	Down
TC08001540.hg.1	− 1.345966	0.001112	Down
TC04000854.hg.1	− 1.345957	0.000384	Down
TC10001536.hg.1	− 1.345921	0.00352	Down
TC05003369.hg.1	− 1.345436	0.000575	Down
TC16000560.hg.1	− 1.344377	0.001315	Down
TC01006043.hg.1	− 1.344027	0.000786	Down
TC04000811.hg.1	− 1.343967	0.000304	Down
TC05000998.hg.1	− 1.343336	0.002569	Down
TC19000170.hg.1	− 1.342442	0.003635	Down
TC17000742.hg.1	− 1.340971	0.002566	Down
TC21000902.hg.1	− 1.340061	0.00187	Down
TC15002522.hg.1	− 1.339697	0.000781	Down
TC12000736.hg.1	− 1.339218	0.003919	Down
TC12003194.hg.1	− 1.339102	0.000544	Down
TC11002900.hg.1	− 1.338332	0.001826	Down
TC04001241.hg.1	− 1.338326	0.000875	Down
TC10001843.hg.1	− 1.337933	0.000309	Down
TC02002711.hg.1	− 1.337676	0.000491	Down
TC07001656.hg.1	− 1.336388	0.001578	Down
TC0X000986.hg.1	− 1.336275	0.001122	Down
TC19000349.hg.1	− 1.336181	0.00149	Down
TC12000238.hg.1	− 1.334203	0.000291	Down
TC09001898.hg.1	− 1.333912	0.001047	Down
TC02001429.hg.1	− 1.332234	0.000635	Down
TC10000827.hg.1	− 1.332045	0.002267	Down
TC13001120.hg.1	− 1.330949	0.001847	Down
TC01006051.hg.1	− 1.330512	0.002522	Down
TC05002063.hg.1	− 1.330054	0.000411	Down
TC13000501.hg.1	− 1.329266	0.001526	Down
TC03001672.hg.1	− 1.328838	0.000468	Down
TC05000595.hg.1	− 1.326941	0.002921	Down
TC0X000282.hg.1	− 1.326422	0.000749	Down
TC16000673.hg.1	− 1.326046	0.001391	Down
TC20000841.hg.1	− 1.325451	0.001234	Down
TC21000149.hg.1	− 1.32514	0.002991	Down
TC14001177.hg.1	− 1.324741	0.002066	Down
TC03000054.hg.1	− 1.324079	0.004008	Down
TC0X001094.hg.1	− 1.323445	0.003145	Down
TC08001239.hg.1	− 1.323048	0.003476	Down
TC01005264.hg.1	− 1.322568	0.000893	Down
TC17002295.hg.1	− 1.322392	0.002183	Down
TC09000452.hg.1	− 1.322086	0.001067	Down
TC08002500.hg.1	− 1.32044	0.000236	Down
TC10002210.hg.1	− 1.316607	0.00393	Down
TC03000845.hg.1	− 1.315832	0.00375	Down
TC06003126.hg.1	− 1.315786	0.00058	Down
TC05000611.hg.1	− 1.31565	0.001065	Down
TC0X000127.hg.1	− 1.314996	0.00143	Down
TC16001996.hg.1	− 1.314795	0.00175	Down
TC08000285.hg.1	− 1.314727	0.001912	Down
TC07002742.hg.1	− 1.312996	0.000481	Down
TC02000971.hg.1	− 1.311939	0.00259	Down
TC0X001939.hg.1	− 1.311233	0.001636	Down
TC10001198.hg.1	− 1.310689	0.001399	Down
TC06001254.hg.1	− 1.310172	0.002178	Down
TC17000164.hg.1	− 1.310162	0.000987	Down
TC01000642.hg.1	− 1.30967	0.001005	Down
TC19000785.hg.1	− 1.309054	0.000687	Down
TC07000478.hg.1	− 1.308947	0.002363	Down
TC09001635.hg.1	− 1.307954	0.000718	Down
TC0X001901.hg.1	− 1.305637	0.000525	Down
TC08001005.hg.1	− 1.305589	0.003865	Down
TC07002764.hg.1	− 1.305001	0.001617	Down
TC01003317.hg.1	− 1.304001	0.000734	Down
TC14002114.hg.1	− 1.303302	0.001654	Down
TC05001142.hg.1	− 1.30304	0.001915	Down
TC20000564.hg.1	− 1.302837	0.000908	Down
TC01005249.hg.1	− 1.30211	0.000911	Down
TC09002102.hg.1	− 1.301834	0.003586	Down
TC10002728.hg.1	− 1.301751	0.001511	Down
TC14000711.hg.1	− 1.301227	0.001088	Down
TC09000942.hg.1	− 1.299524	0.001607	Down
TC22000010.hg.1	− 1.296747	0.00174	Down
TC03002116.hg.1	− 1.296625	0.000773	Down
TC01000843.hg.1	− 1.294777	0.001863	Down
TC12001919.hg.1	− 1.292031	0.002817	Down
TC05001371.hg.1	− 1.291558	0.001479	Down
TC07001870.hg.1	− 1.291055	0.000901	Down
TC01000565.hg.1	− 1.290245	0.000695	Down
TC12002368.hg.1	− 1.290229	0.000606	Down
TC07000399.hg.1	− 1.28954	0.003703	Down
TC19000423.hg.1	− 1.288189	0.002329	Down
TC07002506.hg.1	− 1.28737	0.000997	Down
TC02000092.hg.1	− 1.286776	0.000632	Down
TC13001372.hg.1	− 1.286649	0.001357	Down
TC03003014.hg.1	− 1.286006	0.003111	Down
TC12001455.hg.1	− 1.28555	0.003453	Down
TC0X000233.hg.1	− 1.283645	0.002621	Down
TC19000409.hg.1	− 1.281773	0.001943	Down
TC11000172.hg.1	− 1.280685	0.003312	Down
TC07002026.hg.1	− 1.280371	0.00071	Down
TC07002763.hg.1	− 1.279866	0.000752	Down
TC07002428.hg.1	− 1.279296	0.001135	Down
TC01002397.hg.1	− 1.278713	0.000921	Down
TC13000269.hg.1	− 1.277383	0.002016	Down
TC20000763.hg.1	− 1.276423	0.003633	Down
TC11000705.hg.1	− 1.276067	0.000507	Down
TC06003416.hg.1	− 1.275874	0.000713	Down
TC03000687.hg.1	− 1.275833	0.00199	Down
TC12001821.hg.1	− 1.275762	0.003067	Down
TC01006074.hg.1	− 1.275058	0.002324	Down
TC07000389.hg.1	− 1.274792	0.002663	Down
TC03000673.hg.1	− 1.272024	0.002235	Down
TC10001951.hg.1	− 1.271122	0.000848	Down
TC17001193.hg.1	− 1.270112	0.000804	Down
TC08002312.hg.1	− 1.269922	0.001349	Down
TC04002663.hg.1	− 1.269488	0.002032	Down
TC01004768.hg.1	− 1.269265	0.001148	Down
TC01005463.hg.1	− 1.268925	0.002908	Down
TC16001699.hg.1	− 1.265388	0.00057	Down
TC01005508.hg.1	− 1.265174	0.002194	Down
TC21000491.hg.1	− 1.265062	0.00162	Down
TC03002000.hg.1	− 1.264798	0.001555	Down
TC21000706.hg.1	− 1.264128	0.003956	Down
TC15000396.hg.1	− 1.263935	0.001297	Down
TC20000800.hg.1	− 1.263339	0.001276	Down
TC07002757.hg.1	− 1.263139	0.001002	Down
TC03001494.hg.1	− 1.263001	0.003127	Down
TC10001532.hg.1	− 1.262834	0.002603	Down
TC19000162.hg.1	− 1.262821	0.002081	Down
TC15001184.hg.1	− 1.262007	0.000835	Down
TC15001480.hg.1	− 1.261402	0.001975	Down
TC02001762.hg.1	− 1.259495	0.001305	Down
TC08000460.hg.1	− 1.259301	0.003218	Down
TC08002256.hg.1	− 1.258796	0.00065	Down
TC15001294.hg.1	− 1.257698	0.002676	Down
TC12000589.hg.1	− 1.257295	0.001195	Down
TC03000342.hg.1	− 1.257262	0.004063	Down
TC07002787.hg.1	− 1.25713	0.002561	Down
TC21000925.hg.1	− 1.257035	0.000974	Down
TC03003257.hg.1	− 1.256746	0.000583	Down
TC07002052.hg.1	− 1.256682	0.001404	Down
TC07002196.hg.1	− 1.256575	0.001383	Down
TC17002053.hg.1	− 1.256349	0.001417	Down
TC09000159.hg.1	− 1.255888	0.001346	Down
TC05001001.hg.1	− 1.25565	0.000989	Down
TC05002401.hg.1	− 1.25502	0.001151	Down
TC0X002335.hg.1	− 1.254604	0.002892	Down
TC21000447.hg.1	− 1.25417	0.001539	Down
TC12001854.hg.1	− 1.253701	0.00161	Down
TC02002409.hg.1	− 1.252127	0.00077	Down
TC09002847.hg.1	− 1.251779	0.001474	Down
TC12001777.hg.1	− 1.250828	0.002212	Down
TC20000261.hg.1	− 1.25079	0.002373	Down
TC12000184.hg.1	− 1.249185	0.002347	Down
TC05002180.hg.1	− 1.247857	0.00076	Down
TC08001323.hg.1	− 1.246702	0.00308	Down
TC05002576.hg.1	− 1.246539	0.000807	Down
TC11002740.hg.1	− 1.245999	0.004083	Down
TC04001485.hg.1	− 1.245869	0.00345	Down
TC14001767.hg.1	− 1.245539	0.001281	Down
TC02001858.hg.1	− 1.244836	0.001487	Down
TC02004683.hg.1	− 1.244802	0.001341	Down
TC01000383.hg.1	− 1.244648	0.001641	Down
TC03001231.hg.1	− 1.244214	0.001031	Down
TC05002808.hg.1	− 1.24399	0.00222	Down
TC02003539.hg.1	− 1.243551	0.001615	Down
TC15001493.hg.1	− 1.243527	0.001026	Down
TC16000769.hg.1	− 1.242087	0.003841	Down
TC07003221.hg.1	− 1.241589	0.00302	Down
TC18000715.hg.1	− 1.241272	0.001013	Down
TC17001238.hg.1	− 1.24064	0.001964	Down
TC20000751.hg.1	− 1.24047	0.002572	Down
TC17001774.hg.1	− 1.240464	0.001138	Down
TC01004651.hg.1	− 1.239979	0.003361	Down
TC08000280.hg.1	− 1.239825	0.004026	Down
TC01003671.hg.1	− 1.239653	0.001271	Down
TC06001540.hg.1	− 1.239545	0.003541	Down
TC11002993.hg.1	− 1.239126	0.003369	Down
TC08002317.hg.1	− 1.238759	0.000948	Down
TC12003089.hg.1	− 1.23843	0.000906	Down
TC12001102.hg.1	− 1.237826	0.002626	Down
TC11002750.hg.1	− 1.237261	0.001482	Down
TC12002592.hg.1	− 1.236436	0.003643	Down
TC11001275.hg.1	− 1.235994	0.003359	Down
TC08001580.hg.1	− 1.235099	0.001273	Down
TC17001239.hg.1	− 1.234648	0.00229	Down
TC0Y000302.hg.1	− 1.234355	0.003678	Down
TC0Y000304.hg.1	− 1.234355	0.003678	Down
TC22000194.hg.1	− 1.23392	0.003023	Down
TC02001892.hg.1	− 1.233697	0.002035	Down
TC07001419.hg.1	− 1.233028	0.003205	Down
TC02004514.hg.1	− 1.232891	0.001096	Down
TC11000365.hg.1	− 1.232652	0.003434	Down
TC17001310.hg.1	− 1.232628	0.001917	Down
TC04000223.hg.1	− 1.232273	0.002785	Down
TC02003541.hg.1	− 1.231717	0.002243	Down
TC10002633.hg.1	− 1.231394	0.004024	Down
TC14002045.hg.1	− 1.231387	0.003135	Down
TC14000688.hg.1	− 1.231155	0.001599	Down
TC10000133.hg.1	− 1.231146	0.003936	Down
TC10000136.hg.1	− 1.231146	0.003936	Down
TC01003920.hg.1	− 1.230188	0.002484	Down
TC01003921.hg.1	− 1.230188	0.002484	Down
TC01003922.hg.1	− 1.230188	0.002484	Down
TC01003923.hg.1	− 1.230188	0.002484	Down
TC01003924.hg.1	− 1.230188	0.002484	Down
TC01003925.hg.1	− 1.230188	0.002484	Down
TC01003926.hg.1	− 1.230188	0.002484	Down
TC01003927.hg.1	− 1.230188	0.002484	Down
TC01003929.hg.1	− 1.230188	0.002484	Down
TC01003930.hg.1	− 1.230188	0.002484	Down
TC01003931.hg.1	− 1.230188	0.002484	Down
TC01003932.hg.1	− 1.230188	0.002484	Down
TC01003933.hg.1	− 1.230188	0.002484	Down
TC01003934.hg.1	− 1.230188	0.002484	Down
TC01003935.hg.1	− 1.230188	0.002484	Down
TC01003936.hg.1	− 1.230188	0.002484	Down
TC11000769.hg.1	− 1.229189	0.002433	Down
TC01004020.hg.1	− 1.22836	0.002014	Down
TC22001053.hg.1	− 1.22805	0.003752	Down
TC04001733.hg.1	− 1.227708	0.001127	Down
TC0X000808.hg.1	− 1.22752	0.001289	Down
TC01004433.hg.1	− 1.226181	0.004164	Down
TC21000029.hg.1	− 1.225461	0.001704	Down
TC03002704.hg.1	− 1.224861	0.001445	Down
TC02004897.hg.1	− 1.224747	0.003867	Down
TC09002626.hg.1	− 1.223346	0.001547	Down
TC01000854.hg.1	− 1.223146	0.00242	Down
TC07000408.hg.1	− 1.222936	0.001737	Down
TC01005446.hg.1	− 1.222469	0.002402	Down
TC21000963.hg.1	− 1.219803	0.00291	Down
TC0X001886.hg.1	− 1.219715	0.001036	Down
TC11000031.hg.1	− 1.218532	0.002754	Down
TC07000624.hg.1	− 1.217935	0.002001	Down
TC01006076.hg.1	− 1.217866	0.002136	Down
TC15001040.hg.1	− 1.217766	0.003343	Down
TC06000650.hg.1	− 1.216946	0.001062	Down
TC05001581.hg.1	− 1.216666	0.00138	Down
TC04002772.hg.1	− 1.216379	0.002777	Down
TC02003970.hg.1	− 1.215311	0.001808	Down
TC10002734.hg.1	− 1.21518	0.003732	Down
TC10002413.hg.1	− 1.214979	0.003015	Down
TC0X001785.hg.1	− 1.214397	0.003416	Down
TC18000954.hg.1	− 1.214124	0.003768	Down
TC0X001752.hg.1	− 1.213315	0.002895	Down
TC02003848.hg.1	− 1.213273	0.000843	Down
TC01001745.hg.1	− 1.213018	0.001208	Down
TC03002074.hg.1	− 1.212781	0.001375	Down
TC01002757.hg.1	− 1.211987	0.001696	Down
TC02002040.hg.1	− 1.211577	0.002139	Down
TC21000403.hg.1	− 1.211543	0.002678	Down
TC17002504.hg.1	− 1.211528	0.002074	Down
TC02003570.hg.1	− 1.211373	0.000757	Down
TC01001703.hg.1	− 1.211313	0.002191	Down
TC05002704.hg.1	− 1.211017	0.002259	Down
TC05001346.hg.1	− 1.209244	0.002105	Down
TC07000864.hg.1	− 1.208029	0.002131	Down
TC03002933.hg.1	− 1.208009	0.002345	Down
TC02003270.hg.1	− 1.206919	0.003966	Down
TC17002837.hg.1	− 1.206485	0.003765	Down
TC08002366.hg.1	− 1.205822	0.001688	Down
TC06003509.hg.1	− 1.205272	0.000789	Down
TC07003058.hg.1	− 1.204848	0.003901	Down
TC09000783.hg.1	− 1.204566	0.001995	Down
TC09001972.hg.1	− 1.203884	0.000765	Down
TC02002065.hg.1	− 1.203643	0.002939	Down
TC15001919.hg.1	− 1.203339	0.003106	Down
TC07003162.hg.1	− 1.202549	0.002973	Down
TC15001221.hg.1	− 1.202261	0.003523	Down
TC21000290.hg.1	− 1.201768	0.002413	Down
TC04002818.hg.1	− 1.200872	0.002532	Down
TC06000891.hg.1	− 1.200617	0.002327	Down
TC03000459.hg.1	− 1.200421	0.00082	Down

Probe set ID	Fold change	p-value	Gene feature
(F) *Genes up/down-regulated in purified primary lung TCs compared with non-purified primary lung TCs*			
TC0M000020.hg.1	− 8.322621	2.70E−05	Down
TC0M000022.hg.1	− 2.0304	3.00E−05	Down
TC05002512.hg.1	− 1.586601	3.30E−05	Down
TC04000160.hg.1	− 1.398875	3.50E−05	Down
TC0M000023.hg.1	− 1.833263	3.80E−05	Down
TC03002425.hg.1	− 1.476136	4.60E−05	Down
TC01004110.hg.1	− 1.4988	4.80E−05	Down
TC05003112.hg.1	− 1.883898	5.60E−05	Down
TC07002463.hg.1	− 1.413454	6.10E−05	Down
TC05003313.hg.1	− 1.291542	6.40E−05	Down
TC02004623.hg.1	− 1.595285	7.70E−05	Down
TC12002040.hg.1	− 1.484009	8.50E−05	Down
TC08000385.hg.1	− 1.344602	9.30E−05	Down

**Fig. 5:**
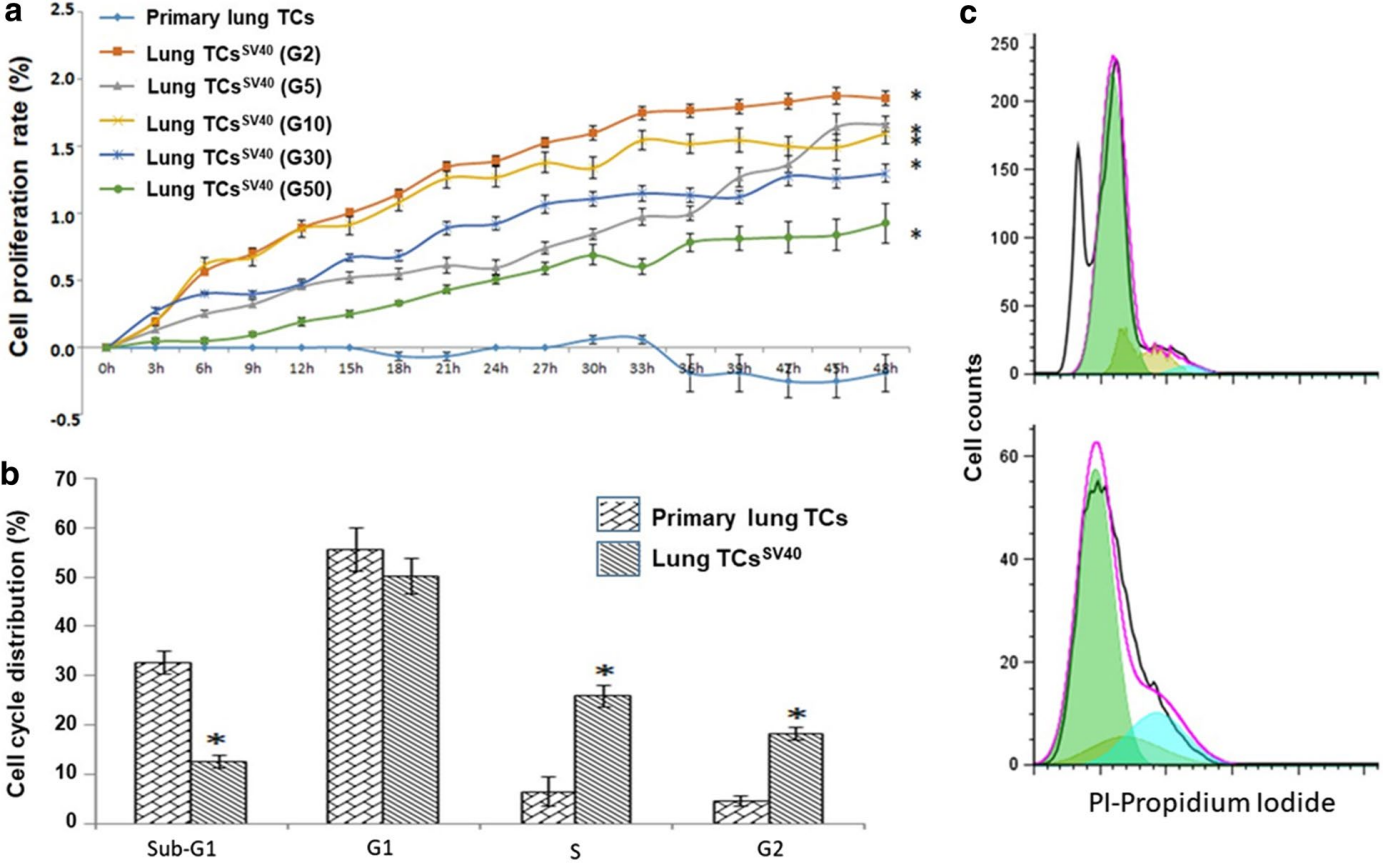
Cell bio-behaviors record and analysis by celliq. **a** The analysis forproliferation rate of primary TCs and TCs^SV40^ within generation 2, 5, 10, 30 and 50. **b** The comparison of sub-G1 phase, G1phase, S phase and G2 phase in primary TCs and TCs^SV40^. **c** Representative pictures of cell cycle phases for primary TCs and TCs^SV40^. *p < 0.05

**Fig. 6:**
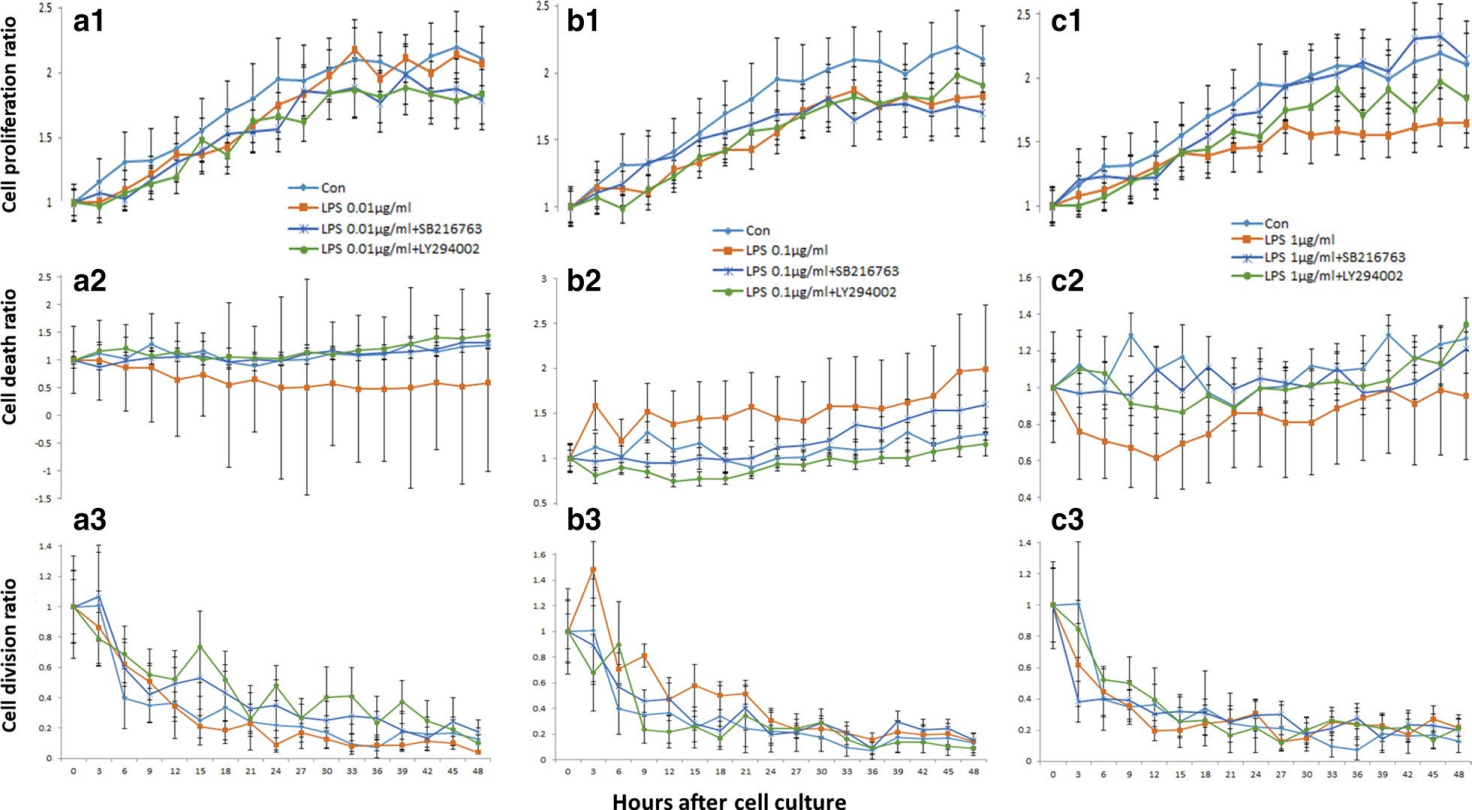
Cell proliferation, cell death and differentiated number analysis of LPS, LY294002 and/or SB216763 stimulated TCs^SV40^ by celliq. **a1**–**c1** Cell proliferation analysis of LPS, LY294002 and/or SB216763 stimulated TCs^SV40^. **a2**–**c2** Cell death analysis of LPS, LY294002 and/or SB216763 stimulated TCs^SV40^. **a3**–**c3** Differentiated number analysis of LPS, LY294002 and/or SB216763 stimulated TCs^SV40^

**Fig. 7:**
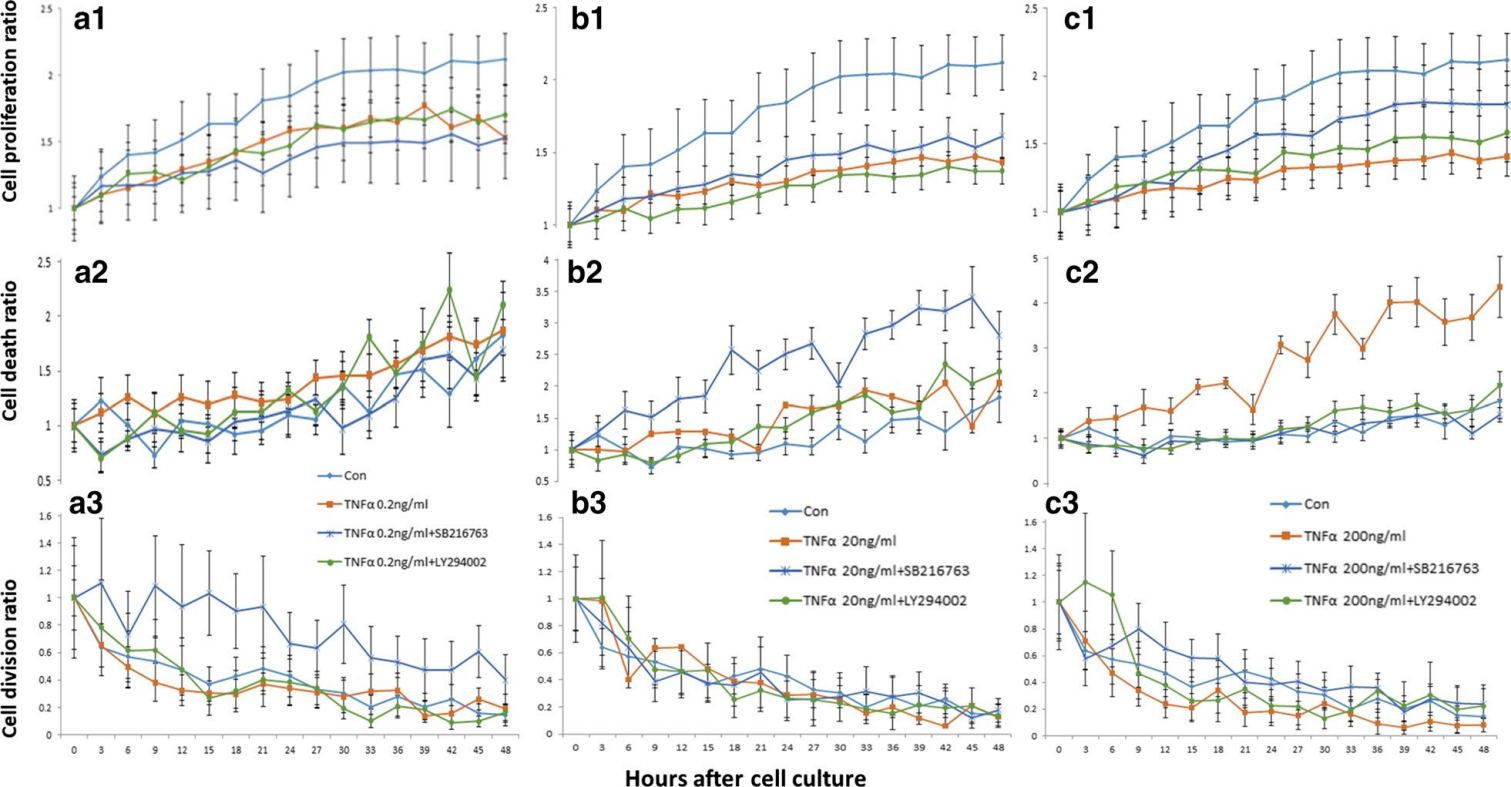
Cell proliferation, cell death and differentiated number analysis of TNF-α, LY294002 and/or SB216763 stimulated TCs^SV40^ by celliq. **a1**–**c1** Cell proliferation analysis of TNF-α, LY294002 and/or SB216763 stimulated TCs^SV40^. **a2**–**c2** Cell death analysis of TNF-α, LY294002 and/or SB216763 stimulated TCs^SV40^. **a3**–**c3** Differentiated number analysis of TNF-α, LY294002 and/or SB216763 stimulated TCs^SV40^

We noticed the proliferation rate of lung TCs^SV40^ at all generations that we detected was significantly higher than that of primary lung TCs^SV40^ (Fig. 5a), of which the highest proliferation rate was observed in lung TCs^SV40^ at generation 2, while the lowest in at generation 50. Lung TCs^SV40^ were mainly located in cell cycle phases of S and G2, while primary lung TCs in sub-G1 phase (Fig. 5b). Furthermore, Fig. 5c demonstrates the alterations of cell cycle phases of Lung TCs^SV40^ compared with primary lung TCs. We found that the number of proliferation (Fig. 6b1–c1) or differentiated TCs^SV40^ (Fig. 6b3) significantly decreased in LPS administration at 1 or 0.1 μg/ml, respectively. LPS at 1 μg/ml caused a significant cell death (Fig. 6b2). Administration with SB216763 significantly inhibited the cell death (Fig. 6b2) or differentiated number (Fig. 6b3) of TCs^SV40^ treated with LPS at doses of 1.0 or 0.1 μg/ml, respectively, while could prevented LPS high dose-decreased proliferating as well as induced cell death (Fig. 6c1, c2). Administration with LY294002 significantly inhibited the cell death (Fig. 6b2) or differentiated number (Fig. 6b3) of TCs^SV40^ treated with LPS at doses of 0.1 μg/ml, respectively, while could prevented LPS high dose-decreased proliferating as well as induced cell death (Fig. 6c1, c2). Figure 7 demonstrates that the ratio of TCs^SV40^ proliferation, death, and dividing after cells were challenged with vehicle or TNFα at different concentrations of 0.2, 20, or 200 μg/ml and treated with vehicle, SB216763 or LY294002. The number of proliferating cells significantly reduced 48 h after administration with TNFα at doses of 200 μg/ml (Fig. 7b1, c1). Cell proliferation, cell death and differentiated number analysis of LY294002 or SB216763 stimulated TCs^SV40^ by celliq were shown in Additional file 3: Figure S2. The representative photos of cell bio-behaviors of TCs^SV40^ stimulated by LPS, LY294003 and SB216763 recorded by celliq were shown in Additional file 4: Figure S3. Additional file 5: Figure S4 showed the representative photos of cell bio-behaviors of TCs^SV40^ stimulated by TNF-α, LY294003 and SB216763 recorded by celliq.

## Discussion

TCs play an important role in the occurrence and progression of acute and chronic lung injury, asthma, and lung cancer [1, 10, 12], responsible for the highest mortality and morbidity of patients. TCs can directly communicate with a large number of cells within organ and contribute to tissue repair and regeneration, as potential alternative of cell therapies [10]. We initially isolated and purified TCs from human lung and airway tissues [16], and defined the special identity and genomic phenomes of lung TCs, different from lung stem cells, fibroblasts, alveolar type II cells, airway basal cells, proximal airway cells, CD^8+^ T cells from bronchial lymph nodes, and CD^8+^ T cells from lungs [4, 5, 17]. Our previous studies have proved the independence and specificity of human lung TCs at genomic levels and proposed TCs as a major source to connect cells, e.g. between TCs per se, or between TCs with other cells. The immunocytochemical markers of TCs include CD34, vimentin, c-kit, CD34/c-kit, CD34/vimentin, or PDGFRα [3, 10, 16, 17]. The specificity of TCs and telocytes-specific biomarkers for the identification are still to be furthermore defined, since there are a large number of telocyte heterogeneities on source, preparation, pathway, duration, and measurable variables. There are obvious differences of TCs among tissues and organs, dependent upon biological functions and characters of TC-connected tissues and organs. TCs in lung have the specificity of connection with air–liquid epithelial cells, tolerance to movement and pressure, and flexibility among barriers. In order to overcome those limits and difficulties, the present study develops a mouse lung telocyte cell-line by gene editing with lentivirus particles containing the anti-aging gene from Simian vacuolating virus 40 (SV40) gene. In the present study, we found morphology, immune biomarkers, and ultrastructure of SV40-positive TCs are similar and coincident with those of primary TCs directly isolated from mouse lungs or cultured for days. Dynamic observations of bio-behaviors demonstrated that TCs^SV40^ proliferation obviously increased, rather than cell movement. The capability of TCs ^SV40^ proliferation declined by increased consecutive passages and became more stable at the 50th passage, which was still significantly higher than the primary TCs. TCs^SV40^ provides a repeatable and stable cell tool for deep investigation of biological roles.

SV40-infection or transformation of lung TCs alters gene expression profiles of cells at certain degrees. SV40 as a polyomavirus with icosahedral capsids of 45 nm and a 5.25 kb-long circular double-stranded DNA can replicate in macaques as its natural host, leading to chronic asymptomatic infections. SV40 small and large T antigen was transduced using lentivirus to immortalize primary cells [18]. SV40 has been strongly considered as a clinical candidate of gene delivery, replacement, or therapy, due to the lack of immunogenicity in humans and capacity to induce immune tolerance to transgene proteins. Toscano et al. [19] used the current SV40 vector genome to generate Vero-based packaging cell vector particles and expresses a large amount of the SV40 large T antigen. It indicates that SV40 delivery can be an approach or alternative of clinical gene therapy. In the present study, mouse lung TCs were transduced and immortalized with a sequence of SV40 small and large T antigen in lentivirus. Excepted for increased capability of cell proliferation, we found that morphology, measured biomarkers, movement, and ultrastructure of TCs^SV40^ are the same as primary TCs directly harvested from lungs. However, we did find a number of gene expression in TCs^SV40^ were different from primary TCs, although the meaning and values of altered gene expression profiles in TCs^SV40^ remain unclear.

There are numbers of questions and considerations on biological behaviors of cells with gene editing, especially about the long-term side-effects and pathophysiological responses of gene-edited cells to challenges. Although the aim of the present study to establish a mouse lung telocyte cell-line for deeply understanding molecular mechanisms of TCs, it should be aware the regulation and translational ethics of preclinical activities for the genome editing [20]. Gene-edited TCs in the present study do not need to perform the large-scale cross-platform comparisons of safety and specificity of those edited TCs as discussed for the potential of clinical applications [21], while we noticed a clear off-target effects of mouse lung TCs on the survival time and passage of TCs. The second passage of TCs^SV40^ had the strongest capacity of cell proliferation, while such capacity declined with the increase of telocyte passage. We found the characterizations of phenomes and functions of TCs^SV40^ were more stable between consecutive passages 5 and 30. Our finding is similar to the report from Taciak et al. [22] that the phenotype and functional stability of the RAW 264.7 cell line were evaluated from the 5th to 50th passage and suggested the RAW 264.7 cell line could remain stable between 10th and 30th passage. Both studies strongly suggest that it should be extremely careful to apply more than the 30th passage of the telocyte cell-line and RAW 264.7 cell line for preclinical research, in order to avoid the questions of data reliability.

TCs^SV40^ showed a dose-dependent response to challenges and therapies which are one of important functional properties of cells. We selected LPS as a stimulus of infection and inflammation and TNFα as an inflammatory mediator at different concentrations treated with or without signal pathway inhibitors and found the proliferative capacity of TCs^SV40^ declined with an increased concentration of LPS or TNFα, similar to responses of primary TCs [23]. Phosphoinositide 3-kinase (PI3K) is a family of related intracellular signal transducer enzymes to phosphorylate the 3-position hydroxyl group of the inositol ring of phosphatidylinositol, perform cellular functions (e.g. cell growth, proliferation, differentiation, and survival), and regulate cell responses to drug therapy [24]. Glycogen synthase kinase 3 (GSK3) is a serine/threonine protein kinase to regulate the addition of phosphate molecules onto serine and threonine amino acid residues, carry out biological function (e.g. cellular proliferation, migration, glucose regulation, and apoptosis), and help cellular reprogramming for clinical cartilage repair [25]. TCs^SV40^ were sensitive to therapeutic effects of both PI3K and GSK3 inhibitors on prevention of LPS or TNFα-reduced proliferation. Of those pathways, GSK3 seems more dominate in telocyte response to challenges. However, more basic and pre-clinical studies are still needed to ensure the safety and efficacy of gene-edited cells before clinical application. Standard and strict application for clinical research ethics must be carried out before the implementation of clinical research. We suggest that lung TCs^SV40^ can be applied for further studies to understand molecular mechanisms by which TCs communicate with other cells.

## Conclusion

Lung TCs play important roles in maintenance of cell–cell communication within the lung and pulmonary function. The present study initially established a mouse lung telocyte cell-line and evaluated the characteristics of TCs phenomes and functions by screening gene expression profiles, ultrastructure, cell biomarkers, and responses to challenges and therapies. We defined the comparability, characteristics and stability in various passages and optimized phenotypic and functional stability of telocyte lines for application. Thus, our data suggest that the newly established lung telocyte cell-line will provide an important tool to understand roles of TCs in maintenance of lung anatomy and functions and develop a new alterative of therapies for lung diseases.

## Additional files

**Additional file 1: Figure S1.** Representative photos of cell bio-behaviors of primary TCs and TCs^SV40^ at 2, 5, 10, 20, 30, or 50 generations recorded for 0 h, 12, 24, 36 h and 48 h captured by celliq.

**Additional file 2: Table S1.** The profiles of transcriptional factor and lncRNA genes (Table S1) between primary lung TCs and TCs^SV40^ were compared and listed Tables 1 and 2, and the hotmap was shown in Fig. 4.

**Additional file 3: Figure S2.** Cell proliferation, cell death and differentiated number analysis of LY294002 or SB216763 stimulated TCs^SV40^ by celliq.

**Additional file 4: Figure S3.** Cell bio-behaviors of TCs^SV40^ stimulated by LPS, LY294003 and SB216763 recorded by celliq.

**Additional file 5: Figure S4.** Cell bio-behaviors of TCs^SV40^ stimulated byTNF-α, LY294003 and SB216763 recorded by celliq.

## Abbreviations

Tps: telocytes
Tps: telopodes
LPS: lipopolysaccharides
SV40: simian vacuolating virus 40 small and large antigen
TEM: transmission electron microscopy
TNF: tumor necrosis factor
SPSS: Statistical Package for the Social Sciences
ANOVA: one-way analysis of variance
PDGFRα: platelet-derived growth factor receptor α
