# Supplementary figures and images for: Influence of gene modification in biological behaviors and responses of mouse lung telocytes to inflammation
Source: J Transl Med. 2019 May 15;17:158. doi: 10.1186/s12967-019-1870-y (PMC6521571; doi:10.1186/s12967-019-1870-y)

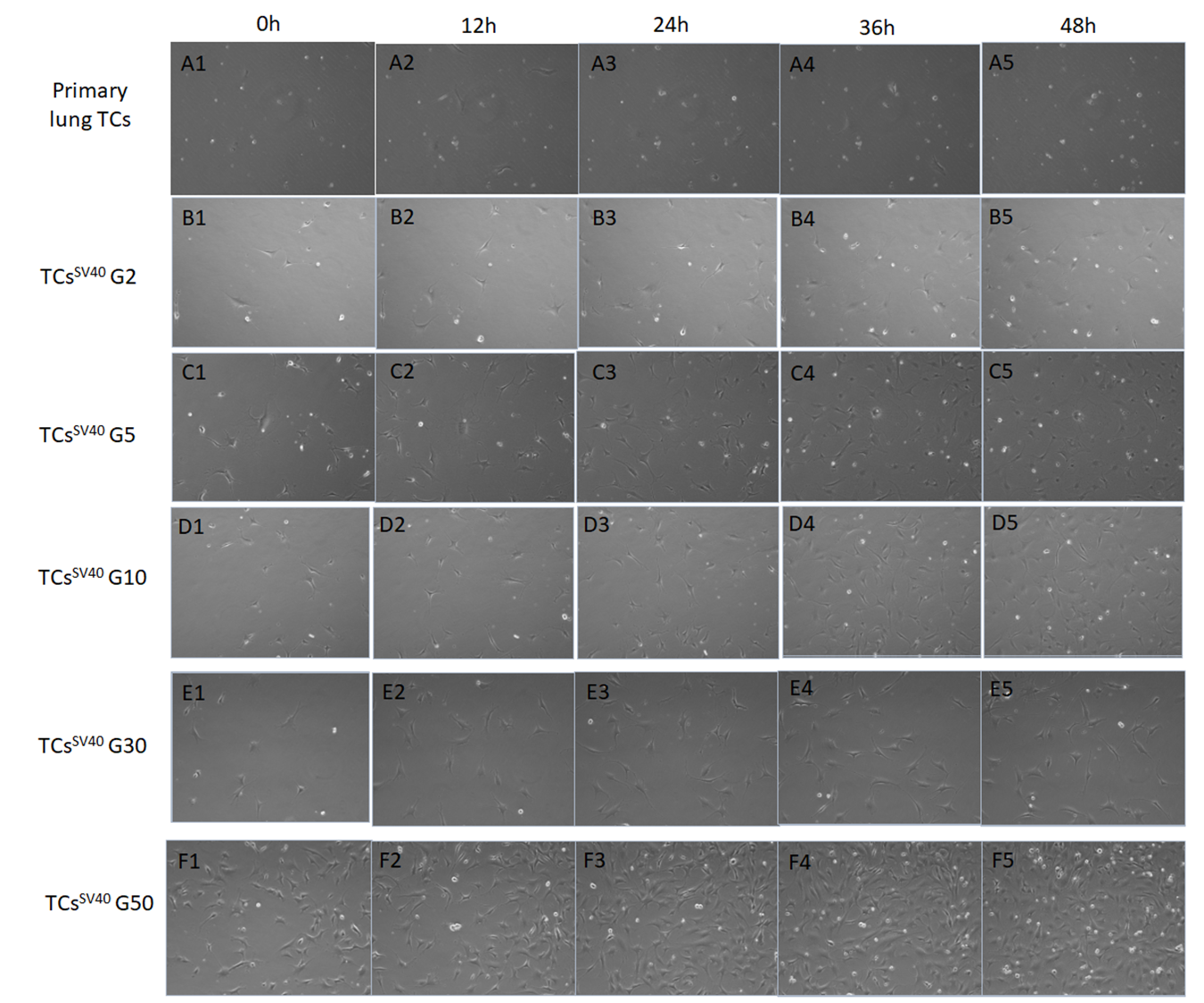

Supplement: Supplementary file 1 — Additional file 1: Figure S1. Representative photos of cell bio-behaviors of primary TCs and TCsSV40 at 2, 5, 10, 20, 30, or 50 generations recorded for 0 h, 12, 24, 36 h and 48 h captured by celliq. [file 12967_2019_1870_MOESM1_ESM.png]

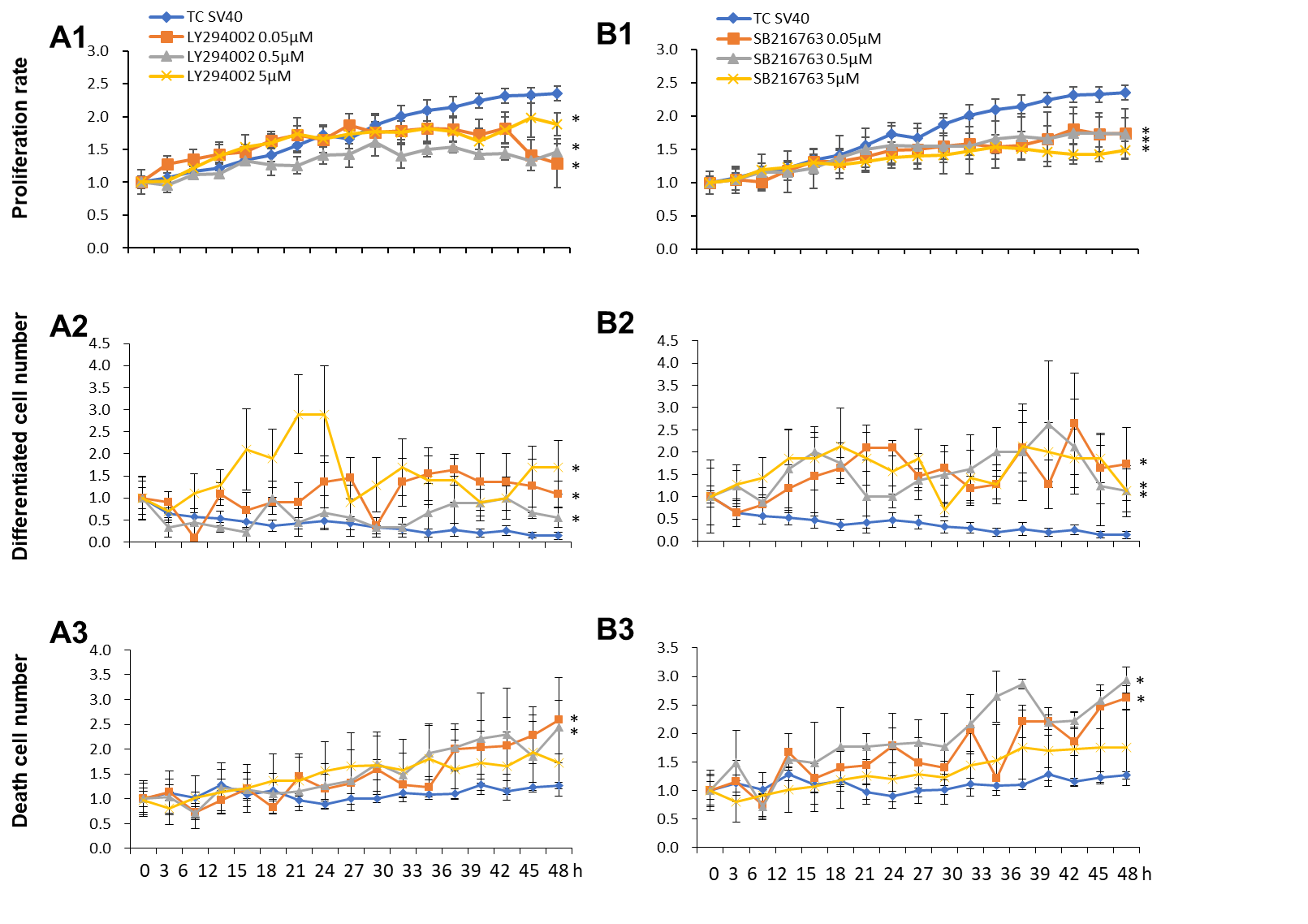

Supplement: Supplementary file 3 — Additional file 3: Figure S2. Cell proliferation, cell death and differentiated number analysis of LY294002 or SB216763 stimulated TCsSV40 by celliq. [file 12967_2019_1870_MOESM3_ESM.png]

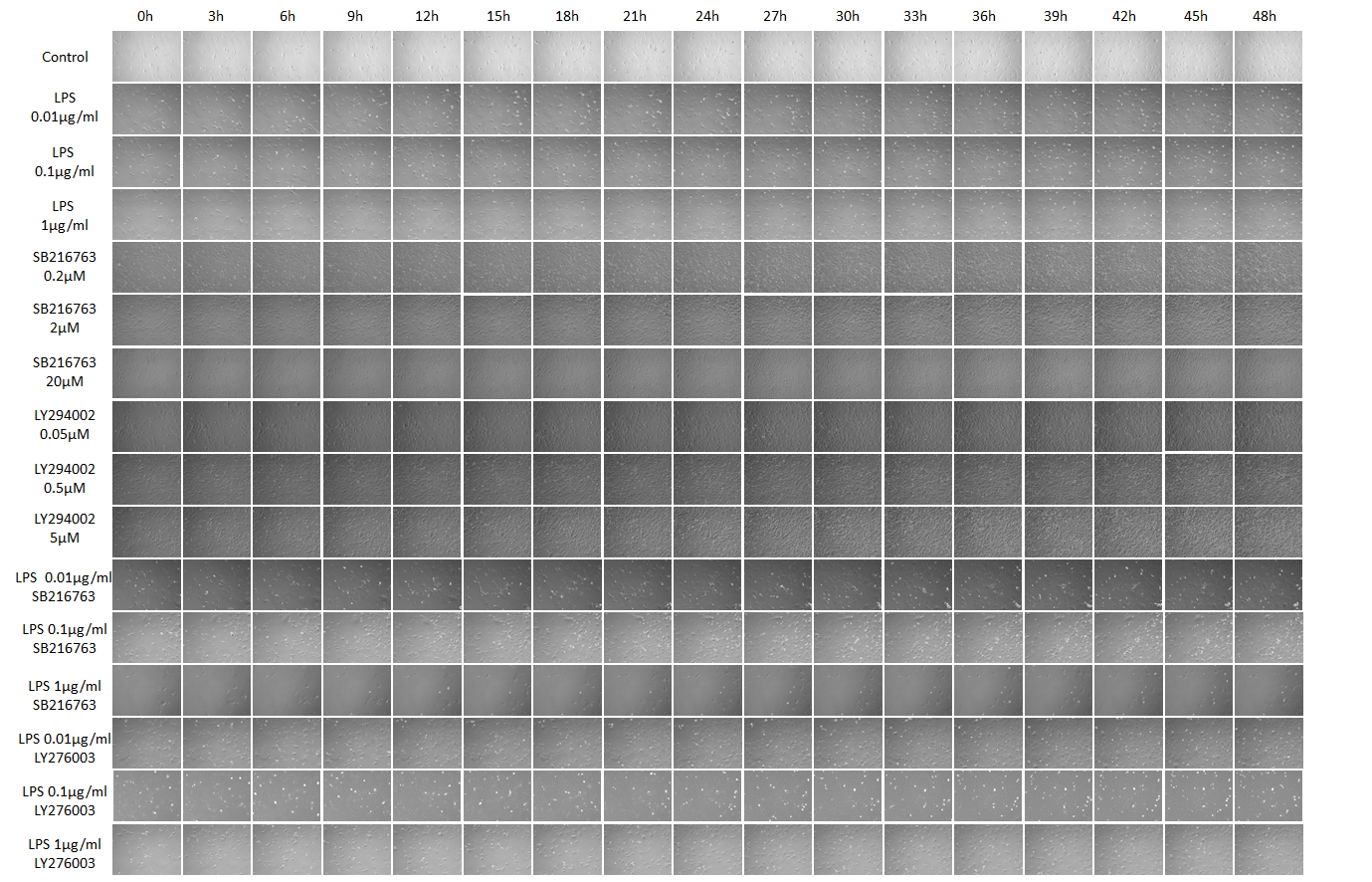

Supplement: Supplementary file 4 — Additional file 4: Figure S3. Cell bio-behaviors of TCsSV40 stimulated by LPS, LY294003 and SB216763 recorded by celliq. [file 12967_2019_1870_MOESM4_ESM.png]

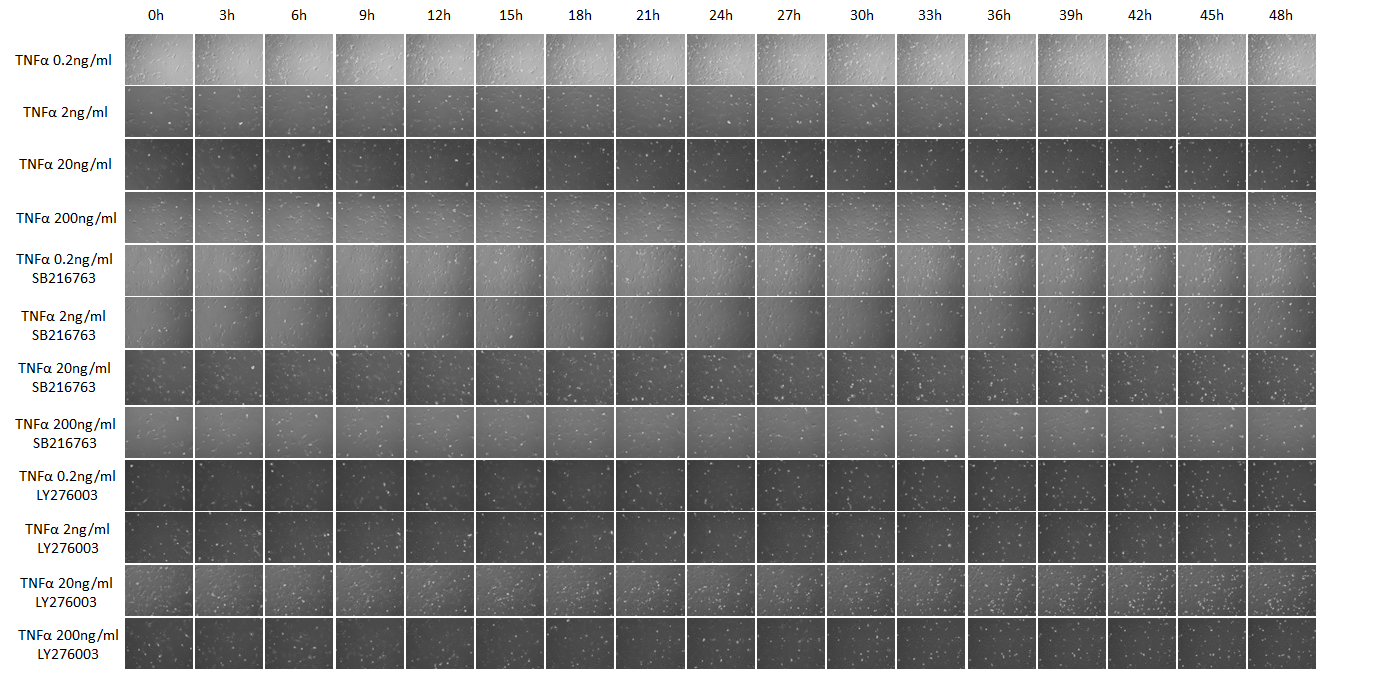

Supplement: Supplementary file 5 — Additional file 5: Figure S4. Cell bio-behaviors of TCsSV40 stimulated byTNF-α, LY294003 and SB216763 recorded by celliq. [file 12967_2019_1870_MOESM5_ESM.png]
